# New York State Climate Impacts Assessment Chapter 06: Energy

**DOI:** 10.1111/nyas.15191

**Published:** 2024-12-09

**Authors:** Sandra Meier, Peter J. Marcotullio, Peter Carney, Susanne DesRoches, Jeff Freedman, Maureen Golan, Justin Gundlach, Jordi Parisian, Peter Sheehan, William V. Slade, Lemir Teron, Ke Wei, Amanda Stevens

**Affiliations:** ^1^ Environmental Energy Alliance of New York Albany New York USA; ^2^ Department of Geography and Environmental Science Hunter College New York New York USA; ^3^ New York Independent System Operator (Ret.) Hague New York USA; ^4^ New York State Energy Research and Development Authority Albany New York USA; ^5^ Atmospheric Sciences Research Center University at Albany Albany New York USA; ^6^ New York Power Authority White Plains New York USA; ^7^ New York State Department of Public Service Albany New York USA; ^8^ Environment, Health and Safety Consolidated Edison Company of New York New York New York USA; ^9^ Department of Earth, Environment and Equity Howard University Washington District of Columbia USA

**Keywords:** adaptation, climate change, demand, energy, impacts, infrastructure, New York State, resilience, supply, vulnerability

## Abstract

Energy plays an integral role in New Yorkers’ lives. It powers the economy, moves people and goods, keeps homes and workplaces at a livable temperature, and runs critical infrastructure that keeps people healthy and safe. Reliable energy systems are easy to take for granted, but many aspects of these systems are vulnerable to weather and climate hazards. This chapter discusses how climate change is affecting and will increasingly affect New York State's energy supply, delivery, and end uses. It provides insights into current and future climate vulnerabilities as New York's energy system transitions to clean energy sources. This assessment also highlights opportunities to adapt current and future energy systems and to build resilience to climate impacts.

## 1 TECHNICAL WORKGROUP KEY FINDINGS

Energy plays an integral role in New Yorkers’ lives. It powers the economy, moves people and goods, keeps homes and workplaces at a livable temperature, and runs critical infrastructure that keeps people healthy and safe. Reliable energy systems are easy to take for granted, but many aspects of these systems are vulnerable to weather and climate hazards. This chapter discusses how climate change is affecting and will increasingly affect New York State's energy supply, delivery, and end uses. It provides insights into current and future climate vulnerabilities as New York's energy system transitions to clean energy sources. This assessment also highlights opportunities to adapt current and future energy systems and to build resilience to climate impacts.

**Key Finding 1: Climate change is already constraining some sources of energy supply and stressing transmission and distribution infrastructure through extreme heat, changes in precipitation, and increasing storm intensity**. Risks to the energy system, and to other sectors that rely on it, will increase as the climate continues to change. Ongoing assessment of climate impacts, responsive investments, and changes to system design and operation will help ensure the continued safe and reliable operation of the energy system.

**Key Finding 2: Patterns of energy demand are shifting due to climate change and are expected to continue evolving over the coming decades**. Altered patterns of energy demand can strain energy supply and delivery (especially during peak periods) and may lead to infrastructure failure and energy price increases. Ensuring a reliable energy supply and delivery will require diverse solutions such as investments in new energy infrastructure, hardening of current energy infrastructure, new business models, and demand‐side management programs.

**Key Finding 3: As New York State's energy system becomes more electrified and more reliant on emission‐free electricity supply sources, new approaches will be needed to adapt to climate change and ensure the system is flexible, safe, resilient, and cost‐effective**. This challenge is heightened by uncertainties on both the demand and supply sides of the electricity system, including renewables’ sensitivity to climate change and the need for new technologies, infrastructure, and operations. Solutions such as demand‐side behavior changes, operational adjustments, investments in system capabilities and capacities, modified business models, and new technologies that are attuned to the changing energy system can moderate the effects of uncertainty due to climate change.

**Key Finding 4: Climate change could result in unequal impacts across communities due to existing inequalities and burdens in New York State's energy system, especially as the system evolves**. People of color and low‐income households are already more likely than other communities to experience challenges in cooling their homes in hot weather and are more vulnerable to power outages during extreme weather events, for example. Responding to climate change gives New York an opportunity to work toward a more just and resilient energy system in which all racial, ethnic, Indigenous, and socioeconomic groups have equitable benefits of clean and resilient energy infrastructure, affordable energy, and associated jobs. Actions such as meaningful community involvement in decision‐making processes can help overcome local barriers and disparities while developing equitable policy choices in the face of a changing climate and an evolving energy system.

BOX 1 **Developments since the 2011 ClimAID assessment**

The energy chapter of ClimAID identified climate change risks that were expected to affect energy infrastructure assets and electricity demand; enumerated adaptation strategies to protect energy supply assets; and presented strategies for reducing energy demand.^1^ ClimAID noted various health and economic losses from power outages, particularly for environmental justice communities. This 2023 assessment evaluates climate impacts on the existing energy sector and on a clean energy future driven by zero‐emission resources. Equity and environmental justice concerns are more fully discussed throughout this chapter, including effects on Indigenous communities. The present chapter also identifies strategies for adaptation and resilience of energy supply, delivery, and demand, as well as areas for further research and investigation.

## 2 INTRODUCTION AND BACKGROUND

Climate change is affecting and will continue to affect both the infrastructure and social aspects of energy supply, delivery, and end uses. The chapter reviews the best available information sources to quantify what is known about impacts observed to date, impacts projected in the decades ahead, and opportunities to adapt and build resilience. It cites and assesses evidence from technical literature as well as from direct engagement with a range of stakeholders, including energy companies operating in different parts of the state, nongovernmental organizations, and community‐based organizations.

This background section provides scope and context for the energy sector in New York State; summarizes key climate hazards and nonclimate factors that affect the energy sector; introduces important considerations around equity, justice, and impacts on Indigenous and other frontline communities; and uses the concept of adaptation as a mechanism for broader positive change. Subsequent sections assess the state of knowledge in more detail as follows:

- **Section** **3** discusses the observed and projected impacts of climate change on three broad parts of the energy system: supply and generation, delivery, and demand. This section reviews impacts on the energy system not just in its current form, but also as it evolves in response to economic and policy factors driving the state's clean energy transition.
- **Section** **4** discusses energy justice and the sources of energy system disparities, recognizing that energy systems disproportionately burden frontline communities, including those that are subjected to increased harm from energy systems and those that do not receive an adequate share of benefits. This section identifies populations and communities at particular risk due to the impacts of climate change and the potential for disparities in adaptation.
- **Section** **5** presents a sampling of adaptation and resilience strategies for the energy sector.
- **Section** **6** examines opportunities for positive change through climate adaptation efforts and identifies emerging topics and research needs. This section also provides a conclusion, summarizing the major findings and recommendations presented in the chapter.
- The **Traceable Accounts** appendix examines each key finding in depth. It provides citations that support each assertion, and it presents the authors’ assessment of confidence in each finding.
- **Case studies** highlight key climate impacts on New York's energy sector, along with adaptation and resilience strategies to protect against these impacts. These case studies are not included in the chapter proper but are available through links provided in the chapter.

### 2.1 Sector scope and context

This section provides an overview of New York State's primary energy supply and end uses, prices, and infrastructure. It also discusses the interdependence of the energy system with other sectors.

#### 2.1.1 Energy supply and use

New York has the nation's third‐largest state economy and is the fourth‐most populous state in the country, yet New Yorkers consume less total energy per capita than the residents of all but two other states—Hawaii and Rhode Island.^2^ New York State has one of the most energy‐efficient economies in the nation, thanks in part to the efficiencies that come with density and the widespread use of mass transportation in the New York City area, as well as the prevalence of sectors (such as finance and professional services) that are not as energy intensive as heavy industry.^2^

As of 2021, New York consumes approximately 3532 trillion British thermal units of primary energy. As shown in Figure 6‐1 (left), 38.5% of that energy is from natural gas, 33.9% is from petroleum products, and 0.21% is from coal, for a total fossil fuel share of about 72.6% (New York phased out coal in electricity generation in early 2020, but a small amount of coal is still used for industrial purposes). Nuclear accounts for 9.2%, hydrogeneration 6.2% (including conventional hydrogeneration and pumped storage hydrogeneration), and 5.9% comes from nonhydro renewable energy sources that includes bioenergy, geothermal, solar, and wind. The state also imports electricity, which accounts for 6.0% of total primary energy.^3^

**FIGURE 6‐1:**
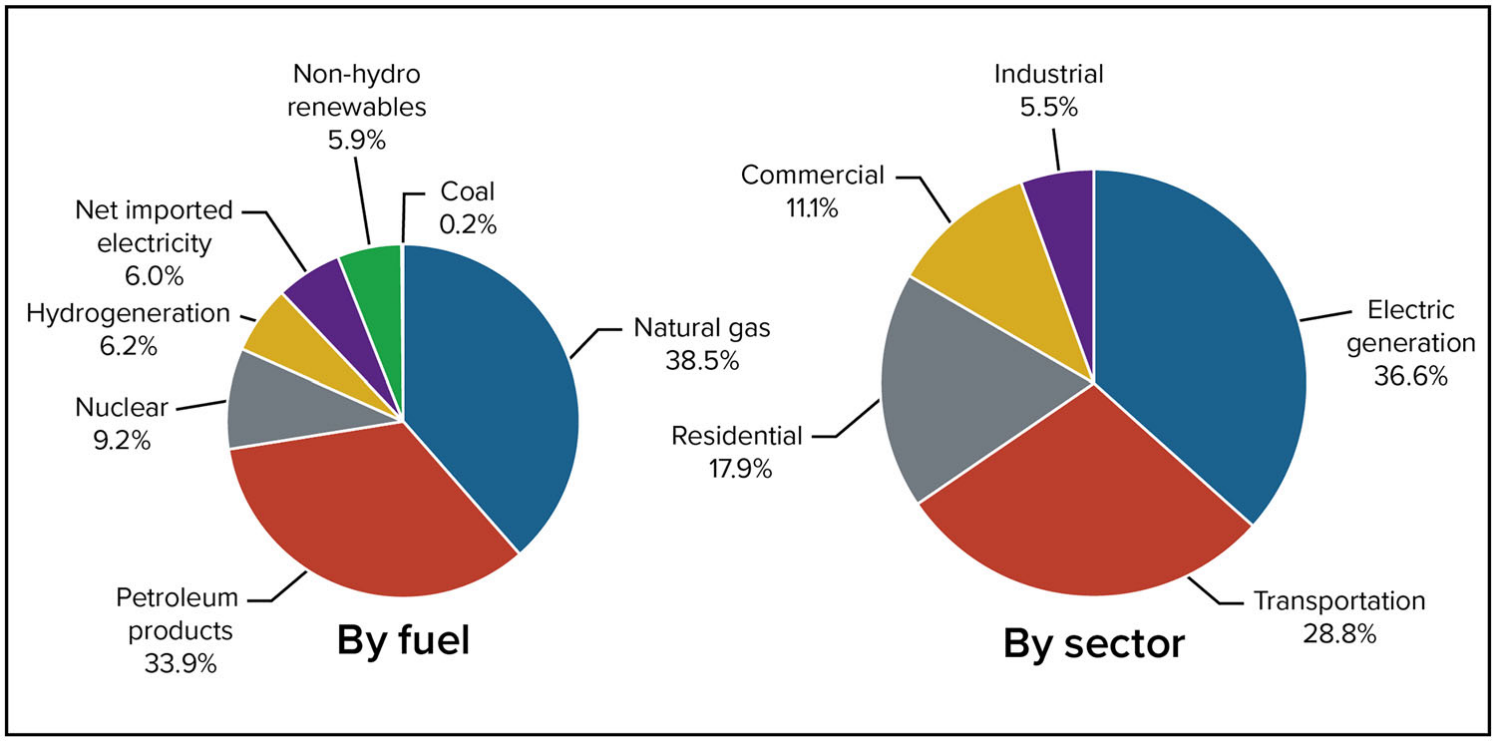
Share of primary energy consumption in New York State in 2021 by fuel (left) and sector (right). Figure from New York State Energy Research and Development Authority (n.d.).^3^

By sector (Figure 6‐1, right), electric generation accounts for 36.6% of total primary energy consumption, followed by transportation (28.8%), residential (17.9%), commercial (11.1%), and industrial (5.5%).^3^

Energy demand varies by season, driven by heating and cooling needs and varying transportation patterns. Electricity use in New York State typically peaks in July and August, when hot weather leads to high demand for air conditioning. Statewide, electricity consumption on peak summer days can be well over 50% higher than daily demand during spring and fall—the seasons with the lowest demand.^4^ The state's power grid must match supply to this fluctuating demand, with enough electric generation resources to meet peak loads. Natural gas also experiences cyclical demand, with peak demand in winter due to its role as New York's dominant heating fuel.

#### 2.1.2 The electricity system

##### 2.1.2.1 System overview

Several organizations work cooperatively to ensure the reliable and safe operation of the power system in New York:

- The Federal Energy Regulatory Commission (FERC) oversees the nation's natural gas and electric power markets, bulk power system operations, and utility system planning.
- The North American Electric Reliability Corporation (NERC) and the Northeast Power Coordinating Council (NPCC) develop, evaluate, and enforce reliability standards that are approved by FERC.
- The New York Independent System Operator (NYISO) is a not‐for‐profit corporation responsible for operating the state's bulk electricity grid, administering New York's competitive wholesale electricity markets, conducting comprehensive long‐term planning for the state's electric power system, and advancing the technological infrastructure of the electric system serving the Empire State.
- The New York State Reliability Council (NYSRC) develops reliability rules specifically for the NYISO and all parties that provide electric transmission, ancillary services, energy, and power transactions on the state's power system.
- The New York State Department of Public Service (NYSDPS) regulates generation and transmission siting as well as electric, gas, steam, telecommunications, and water utilities statewide.
- New York's electric utilities provide electricity to consumers across the state, and seven of those utilities provide electric power service to most customers. There are also two New York power authorities, 49 municipal utilities, and four rural electric cooperatives that provide electric service.^5^

##### 2.1.2.2 Current supply mix

New York State currently relies on a mix of resources for electricity generation—primarily natural gas, hydropower, nuclear, and dual fuel, with the remainder of the supply from wind, solar, biomass, and hydroelectric pumped storage. In 2022, approximately 21% of in‐state electricity generation came from nuclear; 42% from dual‐fuel generators that run primarily on natural gas (but can use other fuels such as oil); 22% from hydropower; and the balance from pumped storage, wind, solar, and biomass (Figure 6‐2).^6^ In addition to the in‐state generation, New York relies on imports to meet its annual electricity requirements. The generation mix varies widely by region (Figure 6‐2), with NYISO load zones grouped into “Upstate” and “Downstate” regions. The two regions generate approximately the same amount of electricity, but Upstate is dominated by nonemitting nuclear and hydroelectric power, while 95% of Downstate generation comes from fossil fuels.

**FIGURE 6‐2:**
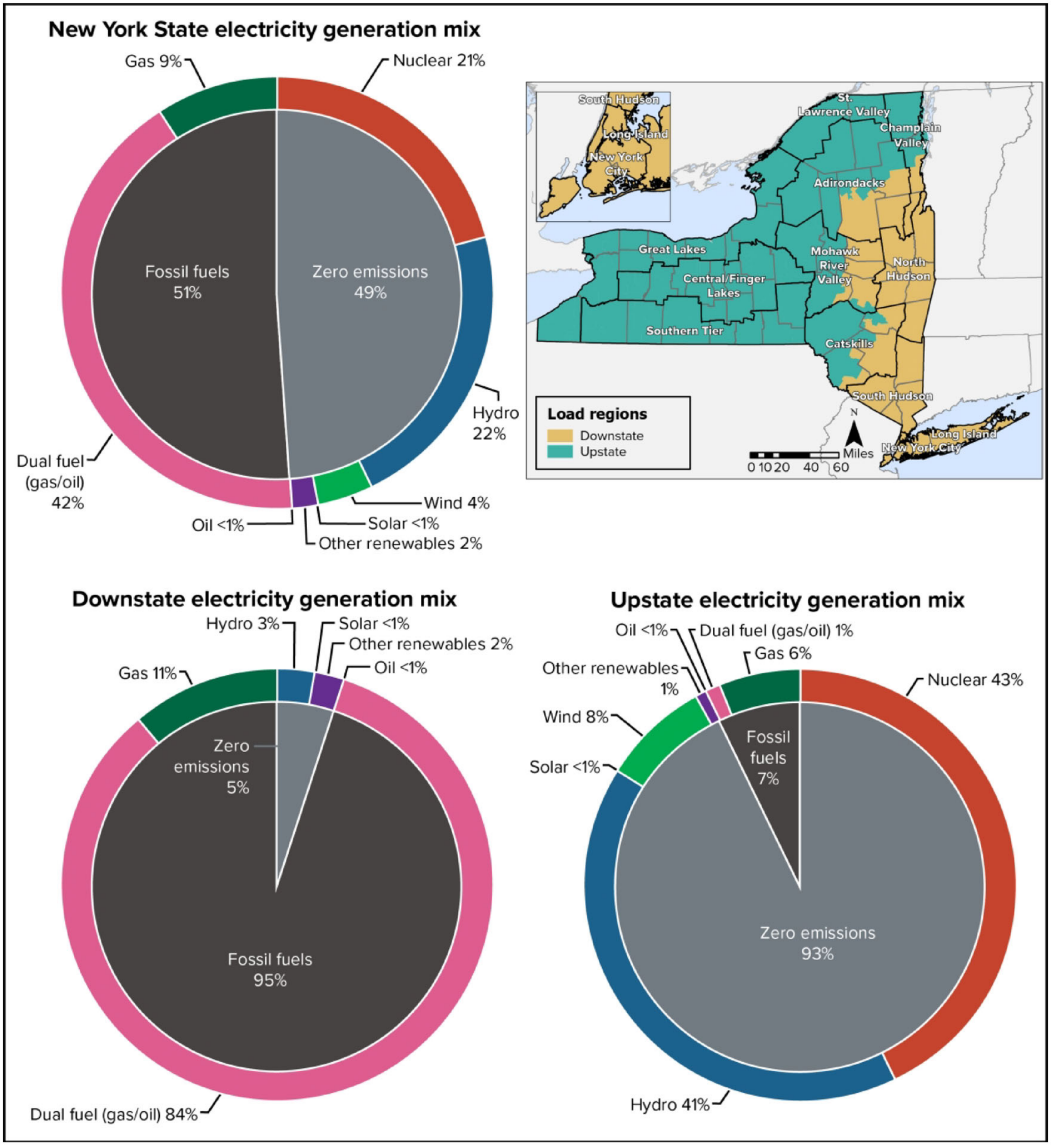
New York electricity generation mix by region. Figure adapted from NYISO (2023).^6^

##### 2.1.2.3 Grid evolution: Renewable supply additions

The electricity supply in New York State is expected to expand through the inclusion of nonemitting resources such as wind and solar, along with battery storage. The 2019 Climate Leadership and Community Protection Act (Climate Act) mandates additions of nonemitting resources to achieve a 70% renewable portfolio standard by 2030 and a 100% zero‐emission electric grid by 2040, as well as specific resource quantities for offshore wind and distributed solar—9000 megawatts of offshore wind by 2035 and 6000 megawatts of distributed solar by 2025.^7^ The achievement of these mandates will shift the supply mix toward a renewable‐dominated energy mix that primarily relies on resources that depend on the weather—solar insolation, wind, and water availability (e.g., precipitation). The concentration of supply in these categories exposes the grid to a new set of weather influences, including high wind, low wind, lack of sun, extreme precipitation, and drought. These factors have little impact on the electric supply of New York today but will have a much greater impact when the state relies more on renewable resources.

##### 2.1.2.4 Grid evolution: Energy storage additions

Current New York State policy requires 3000 megawatts of energy storage by 2030,^8^, ^9^ and in 2022, the Governor directed the storage goal to be increased to 6000 megawatts.^8^, ^9^ Energy storage resources will provide services critical to the grid of the future by balancing loads, providing operating reserves, managing a growing share of intermittent renewables, and reducing congestion. Energy is stored with various technologies that match demands for storage duration, charging and discharging rates, and capacities—each with different vulnerabilities to climate change. Long‐duration storage and emerging clean and dispatchable technologies (also referred to as dispatchable emissions‐free resources, or DEFR), which are yet to be determined, will be needed to balance intermittent resource generation and load requirements. Some of the required storage technologies are not yet commercially available at scale.^10^, ^11^, ^12^, ^13^, ^14^, ^15^

##### 2.1.2.5 Grid evolution: Transmission infrastructure

New York has approximately 18,000 miles of transmission lines, with new lines under construction.^5^ Over the coming years, the transmission system will expand to accommodate the addition of renewable energy and increased electrification (particularly if renewables continue to be distributed geographically unevenly, as in Figure 6‐2), and to address existing constraints that currently affect power flows within the state.^16^ The expansion of transmission investment on local and state levels highlights the importance of transmission infrastructure to ensure adequate supply and flexibility for resilience in the face of climate‐related outages or shortages.

#### 2.1.3 The natural gas system

Eleven major natural gas local distribution companies serve New York State, along with eight small, private, or municipal systems.^17^ As of 2018, the state has 4589 miles of transmission pipelines and 49,307 miles of distribution lines.^18^ There are currently 4.95 million natural gas customers in the state, of which approximately 91% are residential customers, according to annual reports from the NYSDPS and from the U.S. Department of Transportation Pipeline and Hazardous Materials Safety Administration.^19^, ^20^

The natural gas demand cycle is highly weather‐related, while supplies tend to be relatively stable. To ensure sufficient natural gas supplies to meet customer requirements, gas is injected into underground storage facilities during lower‐demand periods, typically April through October, and withdrawn from storage during the higher‐demand winter season. Liquified natural gas (LNG) also plays a role in meeting winter peak day supply for National Grid and Con Edison in New York City. However, with the recent trend toward natural gas‐fired electric generation, demand for natural gas during the summer months is increasing. Natural gas storage, including storage of LNG, also serves as insurance against natural disasters (e.g., hurricanes) and other unforeseen events that could affect gas production and delivery.

Natural gas supplies are expected to remain sufficient to meet demand for New York State.^21^ However, adequate pipeline delivery capacity is critical to ensure that available gas supplies can be provided to the markets. Delivery capacity is especially important during winter system operations in which natural gas competes in the home heating, industrial, and electric generation sectors. Various factors may influence the future of the natural gas system in the state, including demand shifts, the build‐out of renewable resources (including renewable natural gas), and pricing. Long‐term planning activities are currently underway to evaluate scenarios for the future system. Additional details on filed documents, public comments, and interested parties can be found on the NYSDPS website.^22^, ^23^

#### 2.1.4 Other energy commodities

New York State consumes approximately 600,000 barrels, or 25.2 million gallons, of liquid fuels per day for a variety of end uses, such as on‐road vehicles, nonroad engines, marine, aviation, industrial processes, and heating.^24^, ^25^ Power plants that burn petroleum liquids (such as distillate or residual fuel oils) are generally used only for short periods during times of peak electricity demand, such as in New York City during reliability‐related events or during winter when the price of natural gas increases. Otherwise, petroleum‐fired power plants operate at relatively few hours per year because of factors such as the high price of petroleum relative to other fuels, air emissions restrictions, and lower efficiencies of aging generating technology. Dual‐fuel switching from natural gas to fuel oil is more likely during extreme cold weather periods.

All petroleum fuels consumed in New York State are produced in and transported from refineries in other states and countries, including New Jersey, Pennsylvania, Delaware, the Gulf Coast, and abroad.^24^ Most of this supply arrives through ports under the jurisdiction of the Port Authority of New York and New Jersey, which includes one of the world's largest fuel trading hubs and which serves as a regional node for fuels distribution across the Northeast. Supply into the port terminals arrives via major pipelines and marine tankers and barges that bring refined fuel from the Gulf Coast, East Coast, and around the world.

Fuel distribution infrastructure, from pipelines to terminals, is concentrated closer to New York City due to the role of New York Harbor and the high consumption of fuel in this region.^24^ Historically, more than 50% of terminals have been located in Long Island, New York City, and the Lower Hudson region, accounting for roughly 50% of storage capacity.^24^ Fuel is usually stored in bulk storage terminals in and around New York Harbor before it is redistributed to the rest of the state via smaller pipelines and local terminals, and finally by trucks/vehicles to retail sites (e.g., gasoline stations) and end users (e.g., homes, businesses, power plants). Fuels, including propane and biodiesel, are also moved by rail across the state. Key fuel distribution assets will be exposed to different climate risks depending on their location.

The future of liquid petroleum fuels in New York State is uncertain. Research is underway to develop and apply viable low‐carbon alternatives, such as biofuels and renewable diesel. This includes state policy updates that require biofuel blending for home heating oil.^26^, ^27^ New York Harbor remains an important destination for global petroleum product markets, and New York City is an important distribution center for these fuels for the Northeast region. Given these factors, liquid fuels infrastructure and use in the state is likely to remain important into the foreseeable future.

New York State also has more than a dozen district energy systems that centrally generate steam, hot water, or cold water and distribute it to customers via a series of underground pipes. Con Edison in New York City hosts one of the largest district energy systems in the world, with six in‐city plants using natural gas to produce steam that is distributed through closed‐loop piping to approximately 1500 business, residential, and institutional buildings.^28^, ^29^ Smaller district energy systems are located in cities such as Buffalo, Jamestown, Rochester, and Schenectady.^30^, ^31^, ^32^, ^33^

Other fuels used in the state include coal, which is limited but still used in some industries, and hydrogen. While hydrogen use is currently limited, its use and production is undergoing research because of its potential to reduce greenhouse gas emissions.^34^

#### 2.1.5 The cost of energy

Households, businesses, and industries in New York State spent approximately $59.5 billion on energy in 2021.^35^, ^36^ New York ranks the lowest of any state for energy expenditures on a per capita basis.^36^, ^37^ However, the cost of energy can still be burdensome for low‐income New Yorkers; in response, the state administers programs such as the Energy Affordability and Electric and Gas Bill Relief Program to reduce consumer energy burdens. Climate change adaptations that reduce greenhouse gas emissions and proactively incorporate climate and energy justice concerns can reduce these cost burdens.

### 2.2 Key climate hazards

Several key climate hazards affect New York's energy system and are projected to change in the future, such as increasing temperatures; changes in precipitation distribution, including extreme precipitation events; and sea level rise. Changes in wind speed and direction are also important to this sector, although projections are less certain for these variables. Chapter 2, New York State's Changing Climate, provides more detail on these anticipated changes.

- **Increasing temperatures**. Increasing temperatures can lead to a loss of electric generating efficiency in combustion‐based, wind, and solar resources, and could affect demand. Projected increases in the frequency and magnitude of extreme heat and multiday heat waves^38^ could also result in substantial increases in energy demand. Increasing the frequency and magnitude of heat waves could require the derating of transmission and distribution infrastructure, raise component failure rates, and limit maintenance or capital work due to workforce restrictions. Rising water temperatures could also affect the performance of power plants that rely on water for cooling. Cold weather impacts are not a primary focus in this chapter—although cold snaps will continue to occur in New York State, particularly in the near term, extremely cold days are projected to decrease.^38^
- **Changes in precipitation**. Climate change is projected to increase the frequency of extreme precipitation events and the likelihood of short‐term summer drought in New York State.^38^ Fluctuations in precipitation affect river flows and water levels, which in turn can affect hydroelectric generation. Power plants, particularly those in low‐lying areas, are vulnerable to heavy rain and flooding. Heavy precipitation and flooding can adversely affect natural gas supply systems, especially low‐pressure gas distribution systems that deliver gas to residences and small commercial buildings. Damage to liquid fuel infrastructure (e.g., storage tanks, electrical equipment, transport‐delivery infrastructure) can also occur due to heavy precipitation and flooding.
- **Sea level rise**. Sea level rise can affect energy supply facilities in the coastal regions of the state and along tidally influenced upstream waters by decreasing system reliability and damaging buildings and equipment (both high water and the corrosive effects of seawater can cause damage). Sea level rise can also exacerbate the reach and depth of coastal flooding during a storm event. Underground electricity and natural gas delivery infrastructure can be vulnerable to sea level rise, which can lead to outages. Much of the state's fuel infrastructure is in coastal areas, making it particularly vulnerable to the effects of sea level rise. The corrosive effects of seawater and high water can damage infrastructure and cause recurring roadway flooding during high tides. This could prevent workers from safely accessing facilities, which can lead to energy supply disruptions.
- **Changes in wind speed and direction**. Extreme events, such as hurricanes and other storms, affect the energy system in multiple ways. High winds can damage above‐ground electricity transmission lines and utility poles, or cause transmission towers to buckle. High winds can also affect solar electricity generation and damage photovoltaic (PV) panels. A particularly widespread long‐duration event could curtail a large amount of wind generation. Although changes in non‐storm‐related winds are uncertain,^38^ it is worth noting that both lulls in wind speed and very high wind speeds can decrease wind electricity generation, as turbines cannot operate in very high (e.g., more than 25 meters per second) or very low (less than 3.5 meters per second) wind speeds.

Section 3 of this chapter discusses the impacts of each of these climate hazards on New York State's energy system in greater detail.

### 2.3 Nonclimate factors

Many factors unrelated to climate impacts will continue to affect the energy sector, potentially interacting with climatic factors. Of particular significance will be economic and policy factors that change the way New York State produces and consumes energy.

The grid of the future will be driven by the transition to clean energy. Numerous regulations and other policy initiatives aim to decarbonize electricity, buildings, and transportation, mainly through renewable generation and electrification of heat, hot water, and vehicles. This economy‐wide transformation, when combined with climatic changes, has major implications for the resilience and reliability of the energy sector in terms of how end use patterns and the supply‐side mix will change. Various studies^16^, ^39^, ^40^, ^41^ have examined how to achieve both economy‐wide emission reduction targets and clean electricity goals.

The sections that follow briefly describe how these policy mechanisms are changing New York's energy supply and demand, as well as the transmission and distribution systems that connect them. While there are multiple paths to realize a clean energy transition and a net‐zero emissions future, the trends discussed here can help show how the energy sector's evolution will combine with climatic effects to create climate resilience risks and opportunities.

#### 2.3.1 Supply: Low‐carbon energy sources

##### 2.3.1.1 Renewable electric power generation

Electricity generation in New York State will be transformed by the addition of renewable resources. The Climate Act mandates a 70% supply of electric load from renewable resources by 2030 as well as a 100% zero‐emission electric supply by 2040.^7^ These mandates are supported by resource requirements for offshore wind, energy storage, and distributed solar generation.

The transition to a renewable‐driven supply will affect the climate vulnerability of the state's overall energy supply. These clean‐energy targets will result in greater dependence on renewables, storage, and DEFR. The characteristics of renewable resources differ from traditional resources such as combustion‐based thermal power plants, which can be run to match demand. Renewable resources such as solar, onshore wind, and offshore wind generate electricity when the resources are available rather than when they are needed to meet the load. The imbalance between the time that renewable electricity is generated relative to when it is consumed must be managed through energy storage technologies; additional transmission; and, eventually, clean and dispatchable technologies. Renewable energy systems face several challenges such as the intermittency of renewables, uncertainties with weather and load forecasts, and lack of comprehensive control systems.^42^ Renewable power intermittency imposes even larger uncertainties at micro‐scales, as both under‐ and over‐power generation can have a considerable negative impact on reliability.^43^ Moreover, many of the technologies necessary for reliable 100% renewable electrical grids, such as thermal energy storage,^10^ long‐duration energy storage,^11^ and hydrogen storage,^12^ are not commercially available at scale.

The difference between the current and future generation mixes and transmission topologies will require new methods for managing grid operations, including different considerations of statewide reserve margins, local reserve margin requirements, capacity accreditation factors, transmission security modeling approaches, and other reliability and resilience‐based planning and market products. The NYISO studied scenarios^44^ to identify the changes needed to transform the existing electricity generation fleet to meet emissions targets and reliability standards established by the NERC, NPCC, and NYSRC. The metrics used to evaluate reliability are resource adequacy and transmission security. Resource adequacy is the ability of the electric system to supply the demand for electricity while considering scheduled and expected outages of system elements. This is measured against the criterion of one interruption day in 10 years. Transmission security is the ability of the electric system to withstand disturbances without involuntarily disconnecting firm load. This NYISO study is based upon load forecasts that include changes in load patterns due to variations in climate, as well as changes attributable to adaptation through increased electrification. The NYISO concluded that a large portion of the fleet of the future will consist of DEFR, defined by the NYISO as resources that “meet the flexibility and emissions‐free energy needs of the future system but are not yet mature technologies that are commercially available (some examples include hydrogen, renewable natural gas, small modular nuclear reactors).”^44^ The DEFR must satisfy electrical attributes provided today by fossil‐based generation, including sustained on‐demand power (ramping capability as high as 12,000 megawatts per hour).^45^ Twenty‐eight different scenarios were studied by the NYISO, which identified the need for 27–45 gigawatts of DEFRs in 2040.^41^, ^44^, ^46^ The Public Service Commission initiated a proceeding to examine DEFR and other related issues in May 2023.^47^ One of the objectives of this proceeding is to “identify technologies that can close the gap between the capabilities of existing renewable energy technologies and future system reliability needs, and more broadly identify the actions needed to pursue attainment of the Zero Emission by 2040 Target.”

##### 2.3.1.2 Other low‐carbon fuels

Some studies have identified low‐carbon fuels—such as renewable natural gas, hydrogen produced from renewable resources, and renewable liquid fuels—as energy supply options to help decarbonize New York State's energy sector, especially for end uses that are more challenging and costly to shift to a decarbonized electricity sector through electrification of end use demand.^40^, ^41^, ^48^ The role of renewable natural gas and other renewable fuels will vary across sectors, and the climate vulnerabilities of the production, distribution, and consumption of renewable fuels will vary based on the specifics of the sectoral usage patterns.

#### 2.3.2 Demand: Electrification of the transportation and buildings sectors

On the end use side, electrification of vehicles and building heating and hot water are commonly identified measures to help achieve economy‐wide decarbonization. Electrification measures will increase electric demand and change the pattern of demand while offsetting other types of energy commodities (such as natural gas, diesel, gasoline, and steam) as consumers convert from these systems to electric‐based ones. The transition to electrified heating will substantially increase annual energy consumption and peaks, as well as shift peak demand to the winter period. At the same time, warmer winters may decrease heating demand, while extreme heat in the summer may increase cooling demand. The shift in demand patterns has implications for the resilience of system operations, as Section 3.3.1 explores in more detail.

#### 2.3.3 Delivery: Transmission and distribution infrastructure

##### 2.3.3.1 The electric power grid

New York's power grid will see an increase in electric demand as well as a shift toward a winter peak while connecting new generation resources—a combination that the power grid has not had to confront in the past. The location and distribution of renewable resources are based on factors such as land use restrictions, permitting and land ownership, transmission interconnection, and available resources for wind and solar facilities to produce electricity. The distribution of renewable resources will be different from the current distribution of generation plants and will present different resilience and reliability considerations. The addition of offshore wind will cluster a sizable share of the state's generation resources into Atlantic coastal waters, requiring the construction of facilities as well as transmission infrastructure required for the projects to deliver electricity to load centers on land. Offshore technology deployments will face different resilience challenges due to their exposure to conditions unlike those on land.

Major new transmission line projects in New York State will increase the grid's ability to deliver renewable energy for growing demand in Southeastern New York (lower Hudson Valley to Long Island) and reduce the use of fossil fuels for generation in that region. The role of local electricity distribution lines is changing as loads increase with the addition of electric vehicles and electrification of heating and domestic loads. Many distribution circuits will be required to serve as collection systems for distributed solar systems and other distributed resources. Most circuits that serve as collectors will require major rebuilds or upgrades to serve this function.^49^, ^50^

##### 2.3.3.2 Natural gas transmission and electrification of the grid

The U.S. Department of Energy (DOE) estimates that 2−3% of U.S. natural gas is used by oil and gas compressors, including consumption and leakage.^51^ The DOE is investigating technologies and management approaches that could make natural gas transmission more efficient and lead to reduced natural gas use and lower emissions. Alternative technologies include waste heat recovery for electric generation and conversion to electric‐powered main drivers. However, the electrification of natural gas compressor stations may create a new interdependency between the gas transmission system and the electrical grid. The DOE is also investigating the feasibility of hybrid or dual‐fuel compressor technologies that allow compressor stations to run on either electricity or natural gas. The added vulnerability to gas compression created by electrification will need to be considered when the costs and benefits of hybrid compression versus electrical‐only compression are evaluated.

##### 2.3.3.3 Petroleum terminals

Due to the very limited number of petroleum refineries and limited petroleum or renewable fuel plants in New York, the state relies heavily on domestic and foreign imports to meet its fuel needs. Petroleum terminals play a crucial role in receiving, storing, and distributing various petroleum products such as gasoline, diesel fuel, and heating oil, as well as renewable fuels like ethanol and biodiesel. These terminals are essential for ensuring a consistent fuel supply to consumers and businesses in New York. The state is home to around 200 petroleum terminals of different sizes, with a collective storage capacity exceeding 52 million barrels of petroleum. While many of these terminals are smaller “secondary” terminals owned by heating oil distributors, utilities, or local businesses to cater to their specific needs, they are not directly involved in supplying the general public (except for heating oil distributors).^24^

### 2.4 Equity and climate justice

Neither the associated benefits nor the burdens of energy extraction, generation, transmission, and uses are evenly distributed among members of society. Evidence reveals that the benefits and burdens of energy systems, as well as the adverse impacts of climate change, are experienced differently across communities, and that people of color and low‐income households experience a greater share of environmental burdens than other communities while benefiting less than other groups.^52^, ^53^, ^54^, ^55^, ^56^, ^57^

The concept of energy justice addresses these disparities by ensuring that both the social and economic components of energy systems are equity‐minded, and that policy actions focus on addressing the social, economic, and health burdens of communities and populations that have been historically harmed by energy systems.^58^ This is especially important given the energy transitions and climatic effects anticipated over the coming decades. Energy justice necessitates that frontline communities be central players in energy adaptation strategies. It prioritizes safety, accessibility, and affordability while democratizing decision‐making so that all communities can contribute meaningfully to policy.

Climate justice applies these notions to the distribution of climate change burdens and the responsibilities and impacts of society's response.^59^ Energy and climate justice intersect with and are exacerbated by underlying and systemic social and economic stressors and fragilities. Low‐income communities, Indigenous Peoples, and communities of color are more vulnerable to climate impacts due to legacies of displacement, historical and ongoing racial and ethnic discrimination, lack of access to resources, and greater exposure to environmental pollutants.

Two important concepts that frame energy and climate justice—distributive and procedural ethics—are discussed below. These concepts intersect with the related concepts of equity and climate justice in the Assessment Introduction and are discussed further, along with specific vulnerabilities, in Section 4.

The clean energy transition presents an opportunity to address injustices and advance equity. However, as critical end uses become increasingly dependent on electricity service, the need to ensure a resilient and reliable electric system—and equitable access to it—becomes increasingly important.

#### 2.4.1 Distributive justice

Injustices can be identified through inequitable distributions of benefits and burdens across populations. Systematically inequitable burdens fall on specific communities.^55^ In the United States, some communities, including communities of color and low‐income communities, experience more environmental burdens than others.^52^ Disproportionate energy system burdens manifest themselves in frontline communities through higher relative energy costs, greater energy insecurity, greater exposure to pollutants and adverse health impacts, and poorer energy infrastructure. Without responsive policy choices, these disproportionate burdens could worsen over the coming decades as New York's energy system transitions to cleaner energy supplies and adapts to climate impacts.

Additional distributional inequities result from remoteness to energy system benefits. For example, energy injustices often manifest in low access to clean energy jobs. One study suggested that the “transition to a clean energy economy could help address economic inclusion challenges from the national to the local level. However, the current roster of workers in related occupations is far from inclusive—suggesting the existence of distinct barriers to access that require additional attention and action.”^60^ This study finds that “the clean energy economy workforce is older, dominated by male workers, and lacks racial diversity when compared to all occupations nationally.”^60^ The U.S. Bureau of Labor Statistics projects that solar PV installers and wind turbine service technicians will rank among the fastest‐growing occupations in the United States over the next decade.^61^ In New York, these and other jobs in the clean energy sector provide more job opportunities than fossil fuel industries^62^ but are often unavailable to certain communities.

#### 2.4.2 Procedural justice

Procedural justice principles focus on who is included in energy and climate decision‐making processes. The goal of these principles is to provide fair, equitable, and inclusive participation in energy decisions.^63^ Decision‐making procedures involving energy governance do not always include communities that host the new infrastructure (e.g., community solar installations, energy storage projects, transmission and distribution sites).^64^ As New York's energy system adapts to a changing climate, community involvement will be important to ensure that all communities share in the benefits and burdens of the changing energy system. It will also be important to address inequities in the leadership and planning of energy infrastructure decisions and climate adaptation strategies.^60^

### 2.5 Indigenous communities

According to the 2020 Census, New York State is home to almost 400,000 residents with American Indian or Alaska Native heritage, representing 2% of the state's population.^65^ Over one‐third of this population identifies as being solely American Indian or Alaska Native.^65^ Additionally, more than 40,000 residents have Native Hawaiian or Other Pacific Islander heritage.^65^ Of the Indigenous population residing in New York State, about 11,000 people, or fewer than 3%, live within Tribal reservations.^66^

Indigenous communities in New York State have experienced forceable land dispossession because of energy projects. For example, the Kinzua Dam on the Allegheny River near Salamanca in 1965 displaced more than 600 Seneca citizens and submerged 10,000 acres—almost a third of the Seneca territory. This action broke the 1794 Canandaigua Treaty signed by President George Washington, resulted in the loss of fertile soils, and created a deep resentment in the Indigenous community.^67^ In 1960, the construction of the Niagara Falls Power project in what is now Lewiston resulted in the loss of 550 acres of the Tuscarora reservation through eminent domain to form a reservoir. As a result of this project, 37 homes were destroyed and 175 citizens of the Tuscarora Nation were displaced.^68^ Fishing on the reserve, which had played an important role in providing food to Native families, became almost nonexistent.^69^

These and other actions destroyed livelihoods, disrupted connections between social and ecological systems, and created conditions of energy burden and insecurity. For example, electricity access is generally lower on Tribal reservations than elsewhere.^70^ No major power plants are located on Tribal lands in New York,^71^ and many reservations have homes scattered over large areas, far from a utility grid.^72^ These factors, along with the lack of backup generation for power outages, poor access to broadband, lack of financing, and lack of infrastructure training in renewable energy careers, contribute to the energy burden experienced in many Indigenous communities.

Households on reservations are disproportionately without electricity. A DOE analysis determined that 14.2% of households on reservations in the United States had no access to electricity, compared with 1.4% of all U.S. households.^70^ Native Americans also experience disproportionately high energy burdens nationally, regionally, and in metro areas.^73^ In New York State, several Tribes have created climate change adaptation plans to address these issues, with solutions that include using renewable energy.^74^, ^75^, ^76^, ^77^, ^78^

### 2.6 Opportunities for positive change

Adapting to climate change offers opportunities to create positive change, particularly if adaptation is carried out in a way that acknowledges historical injustices and disparities and seeks to close these gaps. Section 5.4 discusses several types of policies and programs that could be targeted toward consumers who are presently the most burdened by energy costs and often among the least resilient to climate‐related impacts.

Policies such as the Climate Act and Subdivision 29 of the Public Service Law 66 are examples of actions that New York State is taking to drive equity and adaptation to climate change, respectively. On July 18, 2019, the Climate Act was signed into law. The Climate Act charged the Climate Justice Working Group with developing criteria to identify disadvantaged communities (note that “disadvantaged” is the term codified in the Climate Act). The working group identified 35% of census tracts in the state as disadvantaged communities, based on criteria that were adopted on March 27, 2023.^79^, ^80^

On February 24, 2022, New York State signed into law an act that added Subdivision 29 to Public Service Law 66.^81^ It required combined gas and electric corporations in the state to conduct a climate change vulnerability study and develop a climate change resilience plan.^82^ The act requires resilience plans to include equity and increased reliability as part of the benefits realized by the proposed adaptation measures. The goal is to build systems that are capable of withstanding and recovering quickly from the exposure to climate hazards, while also providing reliable service during normal conditions.

The state is adapting its energy systems to a changing climate while working to address the root causes of climate change. As noted in Section 2.3, this transition includes greater reliance on renewable resources and distributed resources while also incorporating greater consideration of climate impacts into decisions about energy system design and operations. This compound transition has already begun to yield cobenefits and is expected to yield more. These cobenefits relate to environmental health (e.g., air quality improvements as renewable sources of electricity displace fossil fuels), energy costs, physical comfort, and social justice. Section 6.1 describes these cobenefits in more detail.

## 3 OBSERVED AND PROJECTED IMPACTS ON ENERGY SYSTEMS

### 3.1 Observed and projected impacts on energy supply and generation

This section identifies the potential vulnerabilities of energy supply (e.g., electric power generation and natural gas, oil, and biofuel supplies) to the wide range of climate hazards discussed in Chapter 2, New York State's Changing Climate.

#### 3.1.1 Electricity supply

New York State's supply of electricity can be affected by changes in weather and climate, including temperature (e.g., increasing long‐term mean temperatures; heat waves that are more frequent, longer in duration, and more intense; and less frequent but still intense cold periods that can lead to icing conditions); sea level rise; flooding caused by changes in the amount, timing, and intensity of precipitation; flooding from coastal storm surge events; changes in cloud cover; and changes in wind speed. Each of these climate conditions can affect the electricity supply through direct impacts on the physical infrastructure underpinning generation and through impacts on the mechanical and electrical processes that generate electricity. The ways in which each of these climate conditions affect electrical systems are described in the following subsections.

##### 3.1.1.1 Changes in temperature

Increasing temperatures can adversely affect combustion‐based electric generation in at least two ways: loss of generation efficiency at higher temperatures and loss of cooling capacity as water temperature rises with increasing temperature.^83^ Increases in water temperature can also cause a loss of cooling capacity for nuclear generation. Specifically:

- Increasing air temperatures can reduce the electrical output of turbines that drive electric generators. This problem is exacerbated by increased demand for air conditioning in hotter weather, which adds to the load even as the turbines become less efficient.^84^ Common approaches to dealing with this loss of efficiency and capacity at higher temperatures include intake air chillers and intake air misting systems.
- Research focused on the Northeast has examined the potential impact of increasing water temperature on electricity generation; this research suggests that climate conditions reduce river water available for efficient power plant operations and rivers’ capacity to absorb waste heat, causing a loss of regional thermal generation.^85^ Due to interest in reducing riverine biological impacts, there has been a trend away from wet cooling toward dry cooling for power plants in New York. This increase in dry cooling reduces the likelihood that increasing water temperatures will affect power output systemwide; however, air‐cooled condensers are also subject to efficiency losses (resulting in reduced electric generation) due to increasing temperatures.^86^, ^87^, ^88^
- A study examining various generation plants across the United States found a significant correlation between generator failures and extreme temperatures (both cold and hot).^89^

While generation capacity losses in thermal units due to increasing air and water temperatures may be reduced by the adoption of noncombustion renewables, the issue could remain relevant if power plants that combust hydrogen or renewable natural gas are adopted to supply peak power demands.

Increases in temperature may affect the generation output of PV panels. The output has a direct relationship with the temperature of the panel circuits. A recent New York State Energy Research and Development Authority (NYSERDA) study^90^ found that solar output is reduced by 0.22% per 1°F increase in temperature, which is consistent with other studies,^91^, ^92^, ^93^ although changes in weather variables such as wind, humidity, and cloud‐cover are not accounted for in these results.

For wind technologies, increasing air temperature (and, to a lesser extent, atmospheric moisture) will lead to a slight decline in air density, decreasing power output.^94^, ^95^ Extremely high (or low) temperatures may damage or reduce the life span of rotating components of turbines and blades at wind farms.^94^

As energy storage becomes increasingly important for the reliability and resilience of New York's future power grid, it will also be increasingly important to consider the influence of temperature on the performance of energy storage technologies. Battery‐storage technologies are sensitive to operating temperatures.^96^ Warmer temperatures can reduce battery capacity, efficiency, and life span,^97^ and extreme heat associated with increasing heat waves could require energy storage systems to have internal cooling to prevent increased risks of fires and associated outages at large‐scale battery facilities. Flywheels are storage technologies with characteristics most closely aligned with quick‐response regulation service and are less affected than chemical batteries by changing climate conditions. Flywheels are commonly enclosed in vacuum‐sealed chambers and release energy through the rotation of internal components.^98^ The isolation from exposure to ambient conditions makes this technology resilient to climate change.

Thermal storage for heating and cooling is an established technology for peak shaving and load shifting. The use of off‐peak electricity to meet on‐peak heating demand is economically efficient and with little loss of energy.^99^ An example of thermal storage is using tanks to store chilled water produced with off‐peak electricity to provide cooling for the following day or for longer time periods. The production of chilled water through compression cycles has inherent inefficiencies (e.g., efficiency loss of transforming mechanical work into heat dissipation) that will increase because of rising ambient temperatures associated with climate change. For example, there will be a need to cool water from a warmer starting point, and cooling assets will be exposed to warmer ambient temperatures.

##### 3.1.1.2 Changes in precipitation and flooding

Heavy precipitation and associated flooding can disable supply assets, damage key control and operations equipment through scour and corrosion, and affect ground instability.^83^, ^86^ Transmission line access roads could become inaccessible or washed out, and fixing downed substations and distribution lines could pose access challenges for utility crews. Heavy precipitation and flooding can also cause erosion and landslides, which can affect infrastructure directly through asset damage or indirectly through impeded access. Low‐lying generation facilities could be especially vulnerable to heavy precipitation and subsequent runoff. Extreme events (intense rainfall and short‐term drought) may affect infrastructure and operations and maintenance for smaller hydroelectric generation facilities.

Increases and decreases in precipitation affect river flows and water levels that power hydroelectric generation.^100^, ^101^ Pumped storage could become increasingly valuable during periods when renewable resources are limited by lack of sun or wind, or when peak demand is high. Although smaller run‐of‐river hydrogeneration plants are more susceptible to fluctuations in precipitation and snowmelt than pumped storage, periods of low precipitation can lead to reduced pumped storage capacity.

Seasonal precipitation, runoff, and streamflow are projected to shift in timing and amount as the climate changes.^102^ This variability affects river flow and could affect smaller run‐of‐river hydroelectric plants. A large portion of New York State's clean energy comes from two hydroelectric plants, the Niagara and St. Lawrence Power Projects, which are situated in the Great Lakes watershed. These two power projects are less likely to be affected by the shifts in seasonal precipitation, runoff, and streamflow projected within the state. Rather, the flows at their intakes are dictated by the complex hydrology found in the Great Lakes (e.g., evaporation, precipitation, temperature, soil moisture, snowpack) as well as the international treaties that govern river flow. Climate change is expected to increase the variability of Great Lakes water levels, but there is no scientific consensus as to whether lake levels will increase or decrease overall.^38^, ^103^

The largest reservoir system in New York is the Sacandaga system, which has the current capacity to store 38 billion cubic feet of water.^104^, ^105^ The system feeds Hudson River hydroelectric stations that have a capacity of 340 megawatts. Currently, the primary use of the Great Sacandaga Lake (formerly known as Sacandaga Reservoir) is for flood control as regulated by the Hudson River–Black River Regulating District. In the future, storing water for electricity generation could become a desirable attribute to balance against the recreation and flood control benefits.

As extreme precipitation increases in frequency and intensity due to climate change,^38^ power generation and transmission facilities may need to become more resilient to flash flooding to prevent outages. Widespread flooding affected many areas of the state following Hurricane Irene and Tropical Storm Lee in 2011, causing utility power outages and gas main failures.^106^ Power plants located in low‐lying areas are particularly vulnerable to supply disruptions due to intense rain events.

Evidence on changes in the overall frequency or intensity of freezing rain and ice storms is limited and uncertain, but these events could have impacts on New York's energy system in the future. Ice accumulation could affect generation infrastructure by exceeding its structural design capacity or reducing power output. Wind turbines are particularly sensitive to reduced power output or total failure due to the weight of ice on the blades and other rotating components.^107^, ^108^ Electrical equipment can freeze, leading to short circuits and component and system outages. Key equipment, such as air intakes for compressors, can be subject to icing and difficult to access during icy conditions.^83^ Transmission and distribution lines are also especially vulnerable to ice loading, and design standards may need to be updated as icing projections become more certain.

##### 3.1.1.3 Sea level rise

Sea level is projected to rise by up to 1 foot by the 2030s, about 2–3 feet by the 2080s, and more than 4 feet by the year 2150.^38^ Sea level rise could affect energy supply facilities located in the coastal regions of the state (e.g., New York City, Long Island) and along the tidally influenced portions of the Hudson River (north to Troy) and its tributaries. Sea level rise leads to increased erosion; wave energy reaching farther inland during storm surge; and increased likelihood, intensity, and frequency of flooding of critical energy supply facilities.^109^ Storm surge, nuisance flooding, and long‐term inundation can damage equipment (e.g., through corrosion) and limit facility access. For energy infrastructure on the coast, sea level rise can undermine system reliability and adversely affect routine operations through more frequent flooding of critical facilities such as cooling water intake structures, fuel transfer facilities, and electrical substations.^110^ Saltwater inundation can lead to corrosion and degradation of electrical equipment.^86^ The combination of increased storm winds and wave loads could damage offshore turbine foundations.^95^, ^101^

Sea level rise also increases the potential depth of storm surge events. With increased storm surge depths, larger debris can be carried by floodwaters and collide with structures. The design of existing or planned flood mitigation systems and new generation structures should account for these changes in potential impact forces. Current flood protection for equipment may not be adequate under future sea level rise scenarios.

##### 3.1.1.4 Changes in wind speed

Utility‐scale (e.g., greater than 1 megawatt) turbines generally do not operate in very low (less than 3.5 meters per second) or very high (greater than 25–30 meters per second) winds. There is uncertainty, however, about the degree to which wind speeds will change in New York State and the effects of any changes on wind generation. The Intergovernmental Panel on Climate Change's Sixth Assessment Report indicates, on a global basis, that “observed mean wind speed is decreasing over most land areas where observational coverage is high.”^111^ However, the same report indicates elsewhere that “mean wind speed and wind power potential are projected to decrease in western North America (high confidence) with differences between global and regional models lending low confidence elsewhere.”^111^ Other sources^112^ indicate that the average available wind energy will decrease in the Northeast by less than 15%. Other studies suggest little change in hub height wind speeds (plus or minus 0.2 meters per second) or power production (plus or minus 5% in gross capacity factor) across New York State and adjacent offshore waters.^113^ In a study with different methodologies and assumptions, the NYISO examined the effects of climate disruptions under climate projections to the year 2040 (e.g., severe storms and temperature waves) and the loss of renewable generation due to wind lulls, and found the electric system experienced loss‐of‐load events under stress from these disruptions, even with large amounts of DEFR.^45^

Changes in surface wind velocity could also affect PV energy production.^114^ The flow of wind helps cool down PV circuits to maintain efficiency and capacity; wind lulls would have the opposite effect.^101^ Wind gusts could also cause material damage from debris and create a greater need for cleaning.^115^

#### 3.1.2 Natural gas supply

##### 3.1.2.1 Changes in temperature

Extremely cold temperatures can affect the natural gas supply system by freezing fluid handling equipment, transmitters, sensing lines, valves, and inlet air systems,^116^ but major natural gas supply disruptions in New York State due to extreme cold temperatures have been infrequent. While cold snaps will continue to occur in the state, particularly in the near term, extremely cold days are projected to decrease.^38^ Even under current conditions, standards governing natural gas utilities in the state require measures to protect natural gas infrastructure from freezing, including standards for insulating piping that transports natural gas from wells to storage. If current systems have performed well during historical extreme cold events, their performance is not likely to be affected under projection changes in temperature.

##### 3.1.2.2 Changes in precipitation, flooding, and sea level

The most damaging observed climate impact on the natural gas supply within the state is flooding associated with heavy precipitation,^83^, ^117^ which is occurring more frequently and with greater intensity^38^; flooding events are projected to continue to increase.^38^ Water in underground gas pipelines and tunnels may damage or infiltrate into pipes and cause service interruptions.^118^ As sea levels rise, groundwater levels may also rise in coastal areas,^119^ increasing the exposure of underground infrastructure to groundwater and leading to corrosion. After Superstorm Sandy, Con Edison determined that regulator stations would need to be updated for protection against the increasing impacts of sea level rise.^117^

The 2011 ClimAID assessment^1^ highlighted the damage to New York State's gas supply as a result of major hurricanes in the Gulf of Mexico. Since that time, the amount of gas supplied to the state from natural gas wells in the Gulf of Mexico has decreased dramatically, with much more gas coming from suppliers in states closer to New York.^2^ The vulnerability of these suppliers to hurricanes should be studied, as the Northeast's exposure to this hazard could increase.

#### 3.1.3 Supply of other energy commodities

The upstream production of liquid fuels, including oil, biofuels, and hydrogen, is expected to be affected by climate change—specifically by changes in temperature, precipitation, and flooding. Because less than 1% of power in New York is generated with oil,^18^ this section will focus on oil supply for residences, businesses, and transportation.

##### 3.1.3.1 Changes in temperature

Like natural gas, liquid fuels are more susceptible to cold than to heat. These fuel systems will continue to experience cold temperatures in the foreseeable future, although this exposure will decrease the more the climate warms.

##### 3.1.3.2 Changes in precipitation and flooding

Water from heavy precipitation and flooding events can severely damage certain types of oil tanks.^120^ Over time, small amounts of water from condensation, poor‐quality oil, and ill‐fitting caps can result in corrosion from the inside. Left unchecked, the corrosion will cause the tank to leak. With future increases in heavy precipitation events and flooding, oil storage resources could be negatively affected^110^ for both residential and commercial users, as well as for any storage units on site at electric generating stations.

Some storage tank owners have found that biofuels have caused corrosion in their tanks and/or tank equipment and components. Biofuels with high ethanol concentrations absorb water,^121^ and as fuel absorbs water, layers can form in the stored fuel. This phenomenon also occurs in ultra‐low‐sulfur diesel fuels. According to the Steel Tank Institute, the fuel itself is not causing corrosion; instead, corrosion is caused by the water that the fuel draws in from the environment.

### 3.2 Observed and projected impacts to energy delivery

Climate hazards affect the networks that deliver energy to New York State consumers. These networks include the electric power grid—long‐distance transmission, local distribution, and equipment (e.g., switchgear and transformers) that regulates delivery—as well as transmission and distribution pipes for natural gas and the various systems that deliver liquid fuels to end users (e.g., main pipes and service lines, compressor stations, odorants, and reservoirs).

#### 3.2.1 Electricity delivery

Adverse weather and falling trees are the leading causes of power outages in the state.^18^ Weather‐related damage to the transmission system, which carries electricity long distances from power generation to the distribution system, often leads to more high‐impact and widespread power losses.^122^ Climate hazards of concern include temperature, precipitation extremes, sea level rise, and wind.

##### 3.2.1.1 Changes in temperature

Electrical transmission and distribution systems are sensitive to rising ambient air temperatures.^1^ Transformers, a critical component in distribution or transmission substations, are sensitive to temperatures above their design standard.^123^ High ambient temperatures could lead to planned power interruptions that protect the equipment to prevent damage that would cause longer outages.

Extreme heat can cause substantial increases in load that may exceed the existing installed distribution capacity, require the derating of infrastructure (i.e., diminish its ability to transmit power), increase component failure rates, and limit maintenance or capital work due to lost workdays. Increases in average and maximum temperatures will decrease the delivery capacity of both the overhead conductors of transmission systems and the transformers at utility substations. Increases in ambient air temperature are known to decrease the efficiency and carrying capacity of transmission lines^86^, ^124^ and could cause sagging, which can cause lines to become permanently deformed.^83^ This can present safety and reliability concerns in areas with limited clearance (the distance between the wire or conductor and a grounded object such as vegetation or roads). Safety standards require specific minimum distances between energized conductors and grounded objects to prevent short circuits and loss of service. To avoid damage and outages, a transmission line could be derated at higher temperatures, which could pose risks to the energy supply in areas served by the derated transmission line.

A similar effect occurs in substation transformers at higher average temperatures to prevent damage to the winding insulation within the transformers. Transformers are assigned a reference temperature, above which the capacity of the transformer must be reduced to avoid damage and subsequent outages. The capacity of a transformer decreases when the mean daily temperature exceeds the transformer's reference temperature. The decrease in capacity is approximately 0.38% per 1°F above the reference temperature for substation power transformers.^125^ In New York State, rating specifications for transmission line and substation reference temperatures are currently based on the *New York Power Pool Tie‐Line Ratings Task Force Final Report*.^126^ This report relies on weather station data from 1983 to 1992, which is a short historical time period and does not include recent data. As temperatures rise, ratings based on an outdated temperature reference could result in transmission lines and substations not being able to deliver on their rated capacities, causing mismatches between planning processes and the ability of infrastructure to deliver its expected capacity. The higher the increase in temperature, the larger the mismatch. To account for temperature increases, planning processes would need to rely on updated infrastructure ratings that account for the projected increases in temperature in determining capacity ratings of transmission lines and substation infrastructure.

The loss of conductivity with increasing temperatures is more pronounced in transmission lines than in distribution voltage lines, but the phenomenon is similar.^127^ The resistance of the lines increases linearly with absolute temperature. When coupled with transmission line derates and sagging, these impacts on distribution lines can contribute to local power outages. Climate models project that average temperatures across New York State will increase by 3.8–6.7°F by the 2050s, relative to a 1981–2010 baseline, with periods of extreme heat also increasing in frequency and intensity.^38^ This increase in temperatures may warrant consideration in the design of conductor capacity.

Heat waves could affect the reliability of underground networks at both transmission and distribution voltages by accelerating corrosion and common causes of component failure (e.g., thermomechanical bending or movement).^86,117^ The cables associated with distribution networks heat up during daylight hours due to increased loads, but during a heat wave, they are unable to cool during the overnight period. During extended heat waves, cables can reach critical temperatures, at which point they are either taken out of service, derated, or fail. Transmission cables could also be affected by heat waves and may be derated if the efficiency of the force‐cooling equipment that cools them diminishes. As mean and peak temperatures increase, these derates and load restrictions will also increase if adaptive measures are not taken.

Temperature and heat wave increases are exacerbated by the urban heat island effect in cities, including New York City and other cities across the state. As a result of the urban heat island effect, New York City's urban areas can be hotter than surrounding rural areas, which amplifies heat wave intensity.^128^ The urban heat island effect drives both amplified reductions in transformer capacity and increases in peak load in particularly hot areas of the service territory for Con Edison, the utility that provides electricity to New York City and most of Westchester County. Con Edison^117^ determined that transformer capacity reductions at New York City area substations could be 10% larger relative to expected heat increases in Central Park, which is cooler relative to other areas of New York City because it is covered by vegetation. Similarly, some network centers could experience an increase in peak load nearly 5% larger than in Central Park. As a result of the urban heat island effect, load increases at network centers hotter than in Central Park sum to a total of approximately 350 megawatts.^129^ These spatial heterogeneities represent important factors that could guide long‐term investment planning in heat‐resistant infrastructure and system components. Changes in the intensity and duration of heat waves would have the greatest impact in urban environments, with load increases highest due to concentrated cooling demand and supply limitations exacerbated due to the inability to cool down delivery infrastructure. Incorporating local context and adjusting planning metrics, such as local reserve margins for different regions of New York State's electric grid, may improve adaptation to increasing temperatures and heat waves.

The state's energy systems could also be vulnerable to sudden cold snaps during warmer winters. Although a low‐risk event, frost quakes, also known as “cryoseisms,” could increase in frequency in the future. A frost quake is defined as the unexpected, fast‐action cracking at the surface of water‐ or ice‐saturated groundcover following a rapid temperature drop to subfreezing conditions. Such preconditions to a freeze are more likely to occur as average winter temperatures rise.^130^ The current frequency of such events is low enough to not be accounted for by design codes and standards; however, frost quakes could fracture or shift buried transmission facilities, resulting in forced outages and lost customers.

Increasing temperatures also have the potential to increase lightning, although this result is uncertain.^38^ In its 2021 vulnerability study, National Grid stated that lightning could cause moderate physical damage to generation assets, overhead transmission, towers, and poles.^83^

##### 3.2.1.2 Changes in precipitation and flooding

Heavy precipitation in urban areas can flood electrical equipment manholes and cause outages. The biggest concerns in the future will be flooding and washout of transmission and distribution facilities (underground and overhead), including access roads, and icing on overhead lines. For underground transmission and distribution assets, the primary areas of concern are the open‐air terminations at ground level and the manhole components.^131^

##### 3.2.1.3 Sea level rise

The energy delivery systems in coastal areas of the state (i.e., New York City, Long Island, and the Hudson River estuary) are vulnerable to sea level rise. While overhead facilities are not as vulnerable, underground systems and substations can face substantial risk.^117^ Saltwater intrusion into manholes and vaults can damage underground cables and switchgear, leading to outages. In addition, flooding of street drainage and stormwater systems can impede the operation of automatic sump pumps to remove floodwater from energy facilities. Since Superstorm Sandy, Con Edison has been installing submersible network equipment to restore the system more quickly following a flooding event.

##### 3.2.1.4 Extreme wind events

As discussed in Section 3.1, changes in wind due to climate change are highly uncertain. Hazards associated with tropical and extra tropical cyclones are projected to increase over New York State in the 21st century, while the frequency of such storms remains difficult to project.^38^ High winds from storms can damage overhead lines^86^ when debris and trees are blown against the lines, utility poles are blown over, and transmission towers buckle. High winds from hurricanes will adversely affect energy delivery infrastructure in coastal areas of New York. The remainder of the state, however, is not immune to the impacts of high winds and extreme events. For example, nor'easters can cause extensive damage to overhead lines at both distribution and transmission voltages, particularly if they are accompanied by heavy snow and ice accumulation.^86^ Some of the more widespread power outage events are the result of strong pressure gradients that follow a storm event and produce long‐duration high winds. The increase in storm intensity and frequency because of climate change raises the likelihood of transmission and distribution system damage throughout the state.

#### 3.2.2 Natural gas delivery

Like the underground electrical systems and steam systems discussed above, natural gas delivery will be affected by temperature extremes, heavy precipitation, and increases in flooding.

##### 3.2.2.1 Changes in temperature

A report by National Fuel^132^ indicates that impacts to natural gas delivery systems (i.e., production, midstream, and distribution) in northern Pennsylvania and Western New York are likely to be minimal under two temperature scenarios modeled out to 2030 and 2050. Using two scenarios, one assuming a global temperature change below 4°C by 2100 and one below 2°C by 2100, the company concluded: “Chronic and acute hazards projected under the 4‐degree Celsius warming scenario (SSP3‐RCP7.0) to 2030 and 2050 across the Company's region of operation are not expected to have significant financial impact from physical damage to assets or disruptions to operations.” Overall, the magnitude of direct financial damages to assets represents less than 1% of the average facility value annually, and only 1 day or less of annual business interruption per facility in 2050 under the SSP3‐RCP7.0 scenario—a medium‐to‐high global emissions scenario that is midway between the RCP4.5 and RCP8.5 scenarios modeled for this assessment.^38^, ^132^

Increased variability in winter temperatures^133^ could pose additional risks to the natural gas delivery system. Freeze‐thaw cycles can result in frost heaves, potentially causing pipeline movement or undermining, which could increase pipe stress and fracture.^133^ Warming winters could increase the likelihood of multiple freeze‐thaw cycles in a season. The risk of frost heave is driven by various factors beyond temperature, including the soil type, the buried depth of pipe (e.g., above or below the freeze line), and pipeline materials (e.g., increased risk with older cast iron and bare steel pipe).

Extreme temperature swings can also stress delivery equipment and pipes. Extreme heat could result in greater compressor station cooling needs, although gas demand is substantially lower in hotter months.^134^

##### 3.2.2.2 Changes in precipitation and flooding

The portion of the natural gas delivery system most vulnerable to increased precipitation events is the low‐pressure gas distribution system, which is typically the last part of the system that delivers gas to residences and small commercial buildings. Street flooding from extreme precipitation events and coastal storms can lead to water infiltration in low‐pressure gas mains. Infiltration is especially a concern with older (i.e., pre‐1970s) gas mains made from cast iron or uncoated/bare steel pipes that can be prone to leaks.^135^ Water infiltration can affect the movement of natural gas to residences and commercial buildings.^117^, ^136^ In addition, when the ground becomes saturated, moisture can enter low‐pressure pipes through joints, particularly in portions of the system served by older cast iron pipes. Once water enters the piping system, it can cause reduced pressure or gas outages. The effort by gas utilities in the state to replace leak‐prone pipes lowers the number of pipelines made from cast iron or uncoated steel and reduces the risks associated with water infiltration.

While coastal flooding poses a risk, natural gas distribution infrastructure beyond the immediate coastline is also vulnerable to flooding and high‐intensity hurricane events. For example, in 2011, Hurricane Irene tracked in an atypical course for an Atlantic hurricane and caused major flooding throughout the Catskill and Adirondack mountains and in Vermont. Approximately 1300 customers lost gas service as a result of washouts and flooding events associated with this storm.^137^

The National Fuel report described above noted that other climate‐related hazards projected for other parts of the United States, such as landslides caused by excess precipitation and wildfires from increased temperatures and drought, are not likely to adversely affect their New York operations.^132^

#### 3.2.3 Delivery of other energy commodities

Temperature extremes, precipitation extremes, flooding, and sea level rise pose risks to the delivery of other forms of energy to New Yorkers, including petroleum products (e.g., gasoline, diesel, heating oil), biofuels, and district thermal energy.

##### 3.2.3.1 Changes in temperature

Like other fuels discussed above, fuel oil pipelines can be vulnerable to icing and frost heave.

##### 3.2.3.2 Changes in precipitation and flooding

Flooding due to heavy rains can damage key terminal assets, from electric equipment and wiring to storage systems and related transport‐delivery infrastructure. Similarly, heavy flooding can result in soil erosion, which can undermine pipeline systems and fuel tank foundations, making both more vulnerable to structural impacts.

##### 3.2.3.3 Sea level rise and storm surge

Much of the major fuel infrastructure in New York State is sited in coastal areas and along waterways. These terminals and associated distribution infrastructure are, therefore, vulnerable to flooding from more frequent and severe coastal storms, and from the flooding and physical impacts of sea level rise. Fuel distribution also depends heavily on marine transport, including barges and tankers, which can be affected by coastal flood events. Flooding can inhibit safe navigation to shore, damage onshore facilities, and damage ships via debris in the floodwaters.

Fuel terminals on the coast are vulnerable to damage from storm surge and from the corrosive effects of saltwater exposure. Terminals generally consist of at least five major assets:

- Storage tanks and related containment systems
- Fuel receiving systems (e.g., via marine docks, pipeline interconnection, and/or rail)
- Piping and pump systems (to pump fuel into and out of the tanks)
- Fuel loading systems (e.g., truck loading racks)
- Electrical, environmental, and fire systems

All these assets are potentially vulnerable to the effects of coastal storms and flooding. Superstorm Sandy, for example, severely constrained fuel distribution for much of New York.

Tanks, pumps, loading stations, and docks can all be damaged by storm surge, while saltwater corrosion damages electrical infrastructure and electric‐dependent equipment. Fuel terminals are highly dependent on electricity to run pumps, system communications, and the systems that control compliance with environmental and regulatory requirements (e.g., vapor control). Many terminals do not have backup power generation systems, or their backup systems are not sufficient to meet the full terminal load; this is especially a risk for smaller single fuel‐type terminals.^24^ These terminals are vulnerable to local utility outages.

Even if a terminal can operate under flood conditions, coastal flood events can limit the ability of utility staff to safely access the site, which may in turn disrupt deliveries to end users or retail sites such as gasoline stations. Local and regional damage to road, rail, or waterway infrastructure may further disrupt deliveries and access.

Sea level rise and increased frequency and extent of tidal flooding pose a long‐term risk to marine docks and infrastructure. Coastal flooding can also cause soil erosion, which can lead to pipeline exposure and rupture.

New York State has more than a dozen district energy systems that centrally generate steam, hot water, or cold water and distribute it to customers via a series of underground pipes. District energy systems in coastal areas—including the nation's largest such system in New York City—will be subject to a higher risk of flooding from sea level rise and storm surge. Flooding of steam system manholes and vaults can cause condensation with steam mains, increasing the risk of pressure surges (known as “water hammer”) that can lead to extensive outages.^138^, ^139^

### 3.3 Observed and projected impacts on energy demand

Projected changes in mean and extreme temperatures will have a direct impact on demand for all forms of energy used for heating or cooling. Average, maximum, and minimum temperatures are projected to increase in all regions of New York State under all climate change scenarios through the 21st century. This will increase cooling degree days and decrease heating degree days.^38^ Historically, the “degree‐days” methodology has been used to estimate the heating and cooling energy demand of buildings.^140^

#### 3.3.1 Electricity demand

Several studies indicate that climate change will increase electricity demand. One metastudy^112^ reviewed studies focused on the potential impact of climate change on the bulk power system and found that an increase in electric demand is expected through the end of this century. Other research^141^, ^142^ identifies increased energy demand associated with increasing temperatures. One study^143^ predicted that between 2004 and 2089, the average annual demand from residential and commercial buildings would increase by 17% and the average peak demand from residential and commercial buildings would increase by 42%. Increases in electric demand due to climate change are primarily driven by the impact of rising temperatures on heating and cooling demand, with higher temperatures increasing cooling loads in the summer while potentially decreasing heating loads in the winter. With higher temperatures, more air conditioning appliances will be installed, and the demand from those appliances will rise as more energy is required to achieve the same cooling impact. More frequent, intense, and prolonged heat waves are expected to increase nighttime low temperatures as well, which heavily influences how long air conditioners run and thus the associated electricity demand. Even today, air conditioning can account for one‐third of total electricity demand on a hot summer day in New York State. The NYISO projects that, by 2050, “increasing temperatures will potentially add between 1600 to 3800 megawatts, or 10% to 23% of summer peak cooling requirements.”^144^, ^145^

A NYSERDA study^90^ on the impact of climate change on the energy system, using different methodologies from the NYISO report, also found that higher temperatures will increase cooling demand and decrease heating demand over time. In a scenario with less energy efficiency and fewer heat pumps, aggregate heating and cooling electric load increased from 23 to 31 terawatt hours in 2050 in the more extreme climate case due to increased summer cooling needs. In a Climate Act‐compliant case with greater heat pump and efficiency adoption, increasing temperatures led to overall lower heating and cooling electric load (from 39 to 35 terawatt hours) due to reduced electric demand in the winter months that offset the more limited increase in cooling demand in summer. The study found that warmer extreme temperatures would also affect peaks. In 2050, system peaks in the first scenario, observed in the summer, rose from 34 to 38 gigawatts due to increased cooling demand; in the Climate Act‐compliant scenario, system peaks in 2050, observed in the winter, reduced from 47 to 40 gigawatts under more extreme climate change.

In addition to changes in electric demand driven by climate change, electric demand is projected to grow due to the impacts of electrification in the transportation and buildings sectors. Baseline electricity energy demand is projected to increase by up to 31% between 2021 and 2050.^146^ In its baseline forecast, the NYISO projects that electrification for space heating and transportation may increase electric demand by over 20 gigawatts in 2050, with the shift of heating loads to the electric sector changing the state's peak electric load from summer to winter in the mid‐2030s.^6^ Twenty gigawatts is a large percentage of the current NYISO peak of 32 gigawatts, highlighting the considerable impact of space heating and transportation electrification. Despite an almost two‐thirds increase in loads from building and electric vehicle demand, energy efficiency in the same forecast is expected to moderate peak increases of up to 9 gigawatts.^147^ In addition, based on analysis conducted for New York's Scoping Plan,^148^ end use flexibility mechanisms (e.g., smart devices that can shift energy use when the grid is stressed) can moderate peak increases by over 3 gigawatts in 2050. This finding highlights the value of innovative peak management approaches in managing increasing loads and supporting grid resilience through increased flexibility.^149^

On an annual and seasonal basis, Con Edison, which provides both gas and electricity to its service territory, attempted to determine how changes in heating degree days and cooling degree days would affect the energy needed for its customers in 2050, regardless of whether that energy was supplied as gas or electricity. Their analysis indicated that an increase in summertime cooling degree days in 2050 could result in energy delivery increasing by between 1.4% and 5.4%. The projected increase in electric cooling load over the summer months due to climate change is larger than the estimated decrease in electric heating load due to climate change.^129^

Heat waves increase electricity demand. This increase is driven primarily by cooling needs for building residents, occupants, and critical equipment. In the future, heat waves are projected to become longer, more frequent, and more intense across New York State.^38^ In dense urban environments, the urban heat island effect can exacerbate heat waves. Air conditioning releases hot air into the outdoor environment, which contributes to the urban heat island effect. One study, focused on Arizona, found that this impact is more apparent at night, when heat emitted from air conditioning systems increased the mean air temperature by more than 1.8°F (1°C) for urban locations.^150^ As heat waves continue over consecutive days, the retention of heat and thermal inertia can result in higher cooling needs and associated electricity demand in the latter part of the heat wave. This effect, not uncommon in New York City and parts of Westchester County, will occur even if temperature and humidity are constant during the heat wave. Con Edison uses a rolling multiday weighted average of temperature and humidity, otherwise known as a “temperature variable,” to more accurately predict summer electricity demand within their service territory.^128^

While climate projections for the state show a general warming trend that will lead to increasing cooling degree days and decreasing heating degree days annually, uncertainty remains regarding the impact of climate change on severe winter weather and peak day demand, especially in the near term. Cold days will still occur, and some winters will be colder than others.^151^, ^152^ However, if more people are reliant on electricity during the winter, disruptions to supply from winter weather could have a greater effect than they do today. Nonclimate factors such as electrification will also affect demand. To the extent that the state sees widespread electrification of heating, there could be increasing sensitivity of electric demand to heating degree days, with electric demand potentially spiking during periods of extreme cold. Given state policies aimed at increasing energy efficiency penetration combined with the broader trends that point to widespread electrification of heating and transportation end uses, there is considerable uncertainty regarding the specific trajectory of seasonal electricity growth over time, including both in the summer and winter seasons.

Figure 6‐3 depicts one modeled scenario of the net result of these changes in seasonal electric power demand, with daily load shapes reflecting actual 2022 demand patterns and NYISO's forecasted patterns for 2043.

**FIGURE 6‐3:**
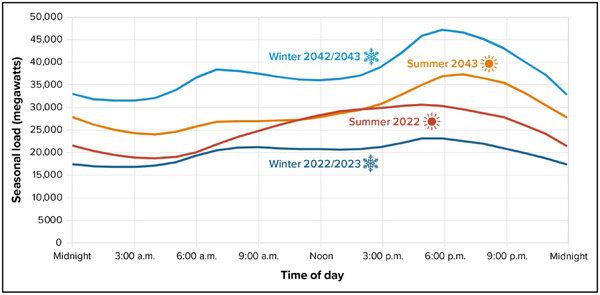
New York Control Area current and projected seasonal load shapes, as modeled by NYISO. Different assumptions of electrification, climate projections, and other inputs may produce different results. Figure adapted from NYISO (2023).^6^

#### 3.3.2 Natural gas demand

Con Edison has projected that its natural gas and steam sectors could both experience sizable decreases in winter energy sales for heating degree days: a 33% decrease by 2050 and a 49% decrease by 2080; the same study also suggests a potential increase in natural gas and steam use for cooling in the summer.^117^ These figures are driven solely by climate change, not by any other changes in demand due to electrification or other factors. Nonetheless, the impact of climate change on gas demand over the course of the 21st century statewide will require additional research and monitoring. It is currently necessary to make certain assumptions about the composition of the energy system in the years ahead to determine the impact of climate change on the demand for natural gas. If the use of natural gas diminishes over time, both in space heating and in power generation, the need for gas supplies from out of state will also diminish, and any potential impacts of climate change on gas supply infrastructure will be reduced.

If the natural gas supply system remains similar to its current construct, it is unclear whether the reduction in heating degree days will offset the increase in cooling degree days such that New York State's current gas system will be adequate to meet future demand in a changed climate.

#### 3.3.3 Demand for other energy commodities

A decline in heating degree days will reduce heating fuel demand.^153^ This reduction will apply to fuel oil and any other fuels used for heating, and it will also apply to district steam systems. A NYSERDA study^90^ on the impact of climate change on the energy system found that warming temperatures would reduce the demand for fuels for heating over time. With electrification of heating and a lower remaining reliance on fuels, fuel cost savings through 2100 was estimated in the range of $12−$27 billion, depending on the scenarios modeled. As noted above, Con Edison has projected that combined winter energy sales from its natural gas and steam sectors could decrease 33% by 2050 and 49% by 2080, with a potential increase in sales during the summer for cooling.^117^

Demand for transportation fuels is not as directly driven by temperature, but could still be affected by climate‐related extreme events and temperature changes that affect traffic patterns.

Increases in the duration and intensity of heat waves could lead to increased reliability and resilience challenges in the electricity sector, which in turn could result in the increased consumption of liquid fuels (e.g., diesel) that are often used as a backup fuel for both power plants and individual backup generators. For the same reason, other climate risks that increase the vulnerability of the electric system could also result in an increase in fuel consumption.

### 3.4 Multisector and cascading impacts

Because all sectors of New York State's economy rely on energy, the energy sector's climate vulnerabilities can result in disruptions throughout the state's economy.^154^, ^155^ Several effects of the extended power failure caused by Superstorm Sandy in 2012 illustrate these cascading impacts.^156^ For example, equipment was disabled or damaged in tunnels where pumps failed and in buildings where electrically powered devices would have protected against inundation. Gasoline rationing and long lines at filling stations ensued, because gasoline could not be dispensed after power failures disabled pumps and other equipment at filling stations.^25^ The Superstorm Sandy's Impact on Public Housing Residents in Red Hook case study explores these impacts in more depth. Vulnerabilities can flow in the other direction as well—climate‐driven disruptions to transportation, communications, and water infrastructure can affect fuel delivery and access to electricity.^157^

Ongoing and expected changes will affect the nature and extent of these interdependencies. Digitalization and reliance on the internet for myriad functions within and beyond the energy sector have already altered the potential for energy sector disruptions—climate‐driven and otherwise—to affect other sectors.^158^ The burgeoning electrification of the buildings and transportation sectors is likely to make historically discrete systems more interdependent, altering how energy sector hazard exposure can translate into vulnerabilities in other sectors.^159^, ^160^ This alteration will be complex and will take a variety of forms, some of which could reduce exposure and vulnerability to climate change, and others of which could increase them.^159^, ^160^

The electrification of sectors such as buildings and transportation, coupled with the increased dependence on digitalization and the internet, will place even greater importance on maintaining the resilience and reliability of the electric system. The expected increase in variable resources such as wind and solar will expose the electric supply system more to weather patterns, but at the same time will feature flexible supply options such as battery storage and nonemitting combustion‐based generation (e.g., power plants fueled by hydrogen). With an expected increase in electrification, demand will also be more affected by climate, as a larger portion of the electric demand in the state will be driven by temperature‐based demands such as heating and cooling.

The agriculture sector will also experience increased electricity demand, as food production under controlled environment agriculture methods (e.g., hydroponic greenhouses) expand in the state and as farm equipment is electrified.^161^

Two back‐to‐back storms in 2011 illustrate how climate impacts on the energy system led to cascading losses across sectors within exposed communities. In late August 2011, Hurricane Irene made landfall in North Carolina as a Category 3 hurricane but was downgraded to a tropical storm by the time it hit New York State. Tropical Storm Lee affected the state a week later, between September 5 and September 10.^137^ Lee compounded the damage caused by Irene, especially in areas where the soil was already saturated and river levels were high. Total rainfall amounts between 8 and 12 inches fell throughout the region.^137^ For New York State Electric & Gas (NYSEG), which serves a sizable portion of the state (ranging from part of Westchester County to the Champlain Valley and the Southern Tier), nearly 199,000 customers lost power over a week in late August.^137^ NYSEG transmission and distribution infrastructure sustained considerable damage, reporting 4959 downed lines.^137^ The utility monitored the rising floodwaters and preemptively shut off gas service and took six substations offline within the predicted flood zone to protect customers and infrastructure.^137^ Road closures impeded utility workers, causing unavoidable delays in service restoration efforts. Crews were able to assess damage and enter affected areas for recovery of the system only after the floodwaters receded. The storm affected 337 NYSEG customers dependent on life support equipment; 13,000 older, blind, and disabled customers; and 31 critical care facilities.^137^

In the NYSEG case described above, telephone landline and cellular service outages made it difficult to reach some affected customers. New York State's telecommunications system, in many ways interdependent with the energy system, is also vulnerable to the impacts of climate change. Telecommunications infrastructure includes:

- The public switched telephone network.
- Cable television networks.
- Wireless/cellular networks; dedicated broadband networks.
- Satellite systems.
- Point‐to‐point microwave networks.
- Broadcast radio and television networks.
- Public/emergency communication networks (e.g., government, first responders, special data transmissions).

Within each network are many points of vulnerability to climate hazards and weather events, which vary based on several factors, including the network elements deployed (e.g., copper, fiber, coaxial cable, radio equipment); the dependency of each network element on commercial power; and whether the infrastructure is deployed aerially or underground. Many of these networks connect with each other, so outages that affect one network can have a cascading effect on other networks and across many voice, video, and data services. For example, in the last 30 years, the transition from copper‐based technologies with analog switching and transmission protocols to digital technologies with hybrid fiber‐coaxial/copper networks has led to increased dependence on commercial power. In this way, the resilience and reliability of modern telecommunications networks mirrors that of the electric grid. These networks also provide the primary backbone transmission support to cellular and internet networks. As with the electric grid, many of the network elements that make up the interconnected telephone network (especially outside of densely populated urban areas) have critical electronic equipment and transmission cables installed on utility poles or otherwise exposed to weather and other hazards. Like the electric utilities sector, the telecommunications sector is addressing climate change, with synergies and cross‐cutting factors through programs such as the Electric Power Research Institute's Climate READi. New York utilities such as the New York Power Authority and the Long Island Power Authority are members of Climate READi and are actively engaged in identifying risks to electric infrastructure and the infrastructure underpinning the electric power grid to improve resilience to extreme weather.^162^

## 4 VULNERABLE POPULATIONS AND COMMUNITIES

While all New Yorkers are vulnerable to the impacts of climate change on the energy system, some are more vulnerable than others—for example, those who experience energy system disparities or work outdoors. This section identifies these populations and communities, the circumstances that increase their vulnerability, and the disproportionate impacts they experience. It first examines the concept of energy justice and sources of energy system disparities. It then discusses populations and communities that are particularly vulnerable to the impacts of climate change on the energy system, and the potential risks they face.

### 4.1 Energy justice and sources of energy system disparities

Energy justice is concerned with the provision, access, affordability, and safety of energy systems so that no community experiences disproportionate energy‐related burdens or is prohibited from enjoying system benefits. Given previous harms, energy justice has a strong focus on frontline communities.^58^, ^163^ Furthermore, it stresses that neither energy system adaptation to climate change nor the renewable energy transition is inherently just or democratizing in terms of the distribution of technologies and benefits. Therefore, energy justice should be explicitly considered and integrated into the design of policies and programs that drive the transition. This includes ensuring procedural energy justice (i.e., the fair, equitable, and inclusive participation in energy decisions). In addition, specific policies that support marginalized communities and vulnerable populations can be put in place to alleviate concerns over energy injustices.^164^

Energy justice addresses disparities within energy systems and focuses on the concepts of energy burden and energy insecurity. Climate change will affect New York's energy systems against a backdrop of many existing disparities. Reviews suggest that these disparities and the resulting energy burdens arise from five main sources: location and geography, housing characteristics, socioeconomic characteristics, energy prices and policies, and behavioral factors.^54^, ^165^, ^166^ Table 6‐1 lists several contributing factors within each of these source categories.

**TABLE 6‐1:** Sources of disparities that contribute to high energy burdens and climate vulnerabilities.

Source category	Contributing factors
Location and geography	Climate zone Ruralness Historically redlined areas Tribal land
Housing characteristics	Thermal integrity Type, age, and size Owner or renter Appliances (age and type) Internet, communications, and information technologies infrastructure
Socioeconomic characteristics	Income Race and ethnicity Immigrant versus native born Age Language isolation Disability status Crowdedness Education Employment
Energy prices and policies	Energy prices Energy policy design Energy mix and access to natural gas Availability and effectiveness of low‐income energy programs and appliances Access to new energy technologies Access to energy industry employment
Behavioral factors	Lack of knowledge Misplaced incentives or principal‐agent problems Lifestyles and cultural factors Lack of control over energy bills High transaction costs

*Note*: Table adapted from Brown et al.^54^ and Hernández.^167^

New York State designates households as having a high energy burden if they spend more than 6% of their annual income on home energy.^168^ In New York City, 18% of families were considered energy burdened in 2017.^168^ Data from weekly Household Pulse Surveys conducted by the U.S. Census Bureau indicate that 10−20% of households statewide kept their homes at an unsafe or unhealthy temperature at least part of the time during 2022 (the approximate range reflects relatively small sample size and week‐to‐week variability in results).^169^

Low‐income, Black, Hispanic, multifamily, and renter households are disproportionately affected by high energy burdens.^55^ Both urban and rural low‐income households spend roughly three times as much of their income on energy compared to high‐income households.^55^, ^170^ Generally, households in Indigenous communities have an average energy burden that is 45% higher than non‐Hispanic white households nationwide.^73^, ^171^ The distribution of energy burden by income status in the state is presented in Table 6‐2.

**TABLE 6‐2:** Annual consumer expenditures in New York State, 2019–2020.

Quintile of income	Residential energy burden (percent of income)
All consumers	4.27
Lowest 20%	16.31
Second 20%	8.97
Third 20%	6.04
Fourth 20%	4.48
Highest 20%	2.48

*Note*: Energy burden is the percent of income before taxes spent on residential energy. Data from U.S. Bureau of Labor Statistics (2022).^172^

In New York State, high energy burden areas are found in both urban and suburban/exurban counties (Figure 6‐4). However, approximately 8% of the state's Black or African American population lives in these census tracts, while 3.5% of the state's white population lives in these census tracts.^173^

**FIGURE 6‐4:**
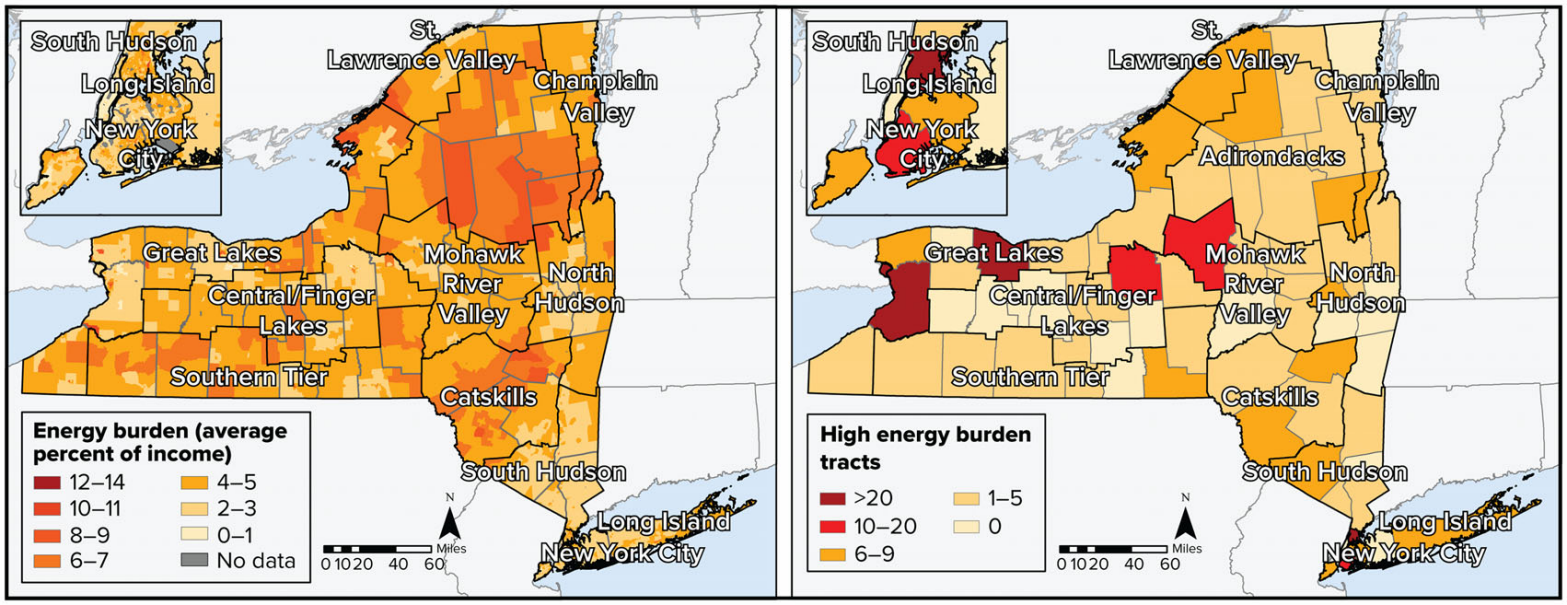
Census tracts with energy burdens as average percent of income (left), and number of census tracts in each county that had an average energy burden (percent of income) greater than or equal to six (right). Data from U.S. Department of Energy (2019)^173^ and Ma et al.^174^

Energy insecurity occurs when households cannot meet their energy needs.^167^ It has also been defined as the uncertainty that a household faces in being able to make utility bill payments.^175^ High energy burden can force a household to forego basic necessities (e.g., food, medicine), and can lead to restrictive unhealthy behaviors and other actions that reduce well‐being.^176^, ^177^ In New York State, 26.7% of households forwent basic necessities in order to pay an energy bill and 17.3% were unable to pay their energy bill at least once between October 2022 and October 2023.^178^ Those facing energy insecurity may be homeowners unable to invest in efficiency upgrades, or they may be renters living in housing units where landlords do not pay for the utilities and consequently have little incentive to create more energy efficient units.^179^ U.S. Energy Information Administration Residential Energy Consumption Survey data for 2015 suggest that 31% of U.S. households experienced some form of energy insecurity.^180^ That year, nearly 7 million households had their access to heat interrupted at least once, and 6 million lost access to air conditioning at least once.^181^

### 4.2 Populations and communities at risk due to climate impacts on the energy system

Households, populations, and communities already suffering from energy burdens and energy insecurity may be disproportionately at risk of further harm due to climate‐related disruptions. These vulnerabilities can stem from increased exposure to climate hazards, increased sensitivity to climate hazards, and/or diminished adaptive capacity.^182^

Populations facing heightened risks due to climate impacts on the energy system are discussed below.

#### 4.2.1 Low‐income populations, people of color, and Indigenous Peoples

Research has found that low‐income households and those facing energy insecurity often face acute vulnerability to climate impacts within the energy sector. These vulnerabilities include economic challenges posed by the expense of heating and cooling homes during temperature extremes, which can result in increased morbidity and mortality as well as food insecurity^183^ and heightened vulnerability during and after extreme weather events.^184^ Low‐income households and communities of color are more vulnerable to power outages than their neighbors in New York City during the warm months of the year^185^; such power outages could become more common in a changing climate. These communities also face risks of utility disconnections and disaster‐related discontinuation of energy services.^176^ Preliminary evidence from a New York City‐based study shows that power outages pose substantial threats to communities that are located in urban heat islands and disproportionately low‐income, especially communities of color, and that air conditioning equity issues could worsen with the advance of climate change.^186^ Income constraints, lack of private transportation, and the lack of capital (and credit cards) limit temporary residency options, such as hotels, in the aftermath of extreme weather events.^184^ Research has shown that power is slower to be restored to rural and Black communities in the aftermath of extreme weather.^187^ Indigenous peoples experience disproportionately high energy burdens nationally, regionally, and in metropolitan areas,^73^ which can make them more vulnerable to energy‐related climate impacts.

Consequences of energy insecurity can also include extreme home temperatures, hazardous heating alternatives, and the threat of utility shutoffs because of nonpayment of utility bills. Evidence also suggests that the stress from this insecurity can adversely impact health.^188^ This problem is especially acute for low‐income residents, such as single parents, older adults, people with disabilities, and others with low or fixed incomes.^189^, ^190^

#### 4.2.2 People with underlying health conditions, older adults, and younger people

Those with underlying health conditions, older adults, and younger people are very susceptible to health impacts from extreme temperatures,^191^ so power outages caused by extreme events or increased temperatures can be a major concern for these populations. In addition, these populations may rely on electricity‐dependent medical supplies. Households with low incomes and high energy burdens are more likely to use high‐emission heating and cooking methods that lead to poor indoor air quality that is particularly harmful to children and to adults with pre‐existing health conditions, such as respiratory disease and asthma.^192^ Energy issues that exacerbate these health conditions could make these populations even more at risk of heat‐related morbidity and mortality.

High concentrations of pollution combined with high temperatures, the lack of green spaces, lack of cooling equipment, lack of resources and cooling centers, and lack of health care create difficult circumstances for communities residing in urban heat islands, which also tend to be overburdened communities. For people who are already sick due to their living environment, especially children and older adults, these injustices continue to accumulate over time, taking a toll on their well‐being and quality of life.

#### 4.2.3 Outdoor energy workers

Like all outdoor workers, workers in the energy sector face increased risks to their health, safety, and productivity from rising temperatures and extreme weather from climate change. High temperatures can create unsafe work conditions through heat stress and poor air quality. These conditions can lead to a loss in productivity or workdays, which can place strains on the energy system and threaten its reliability.^193^ Extreme climate events such as storms or heat waves can cause power outages. During these events, energy workers become frontline responders and face expanded risks as they work to restore power. The Human Health and Safety chapter includes additional information about health risks and adaptation options for outdoor workers.

## 5 ADAPTATION AND RESILIENCE STRATEGIES

Adapting the energy system to climate change will require responses to the many pressures described in Section 3. This section provides examples of adaptation strategies specific to energy supply (Section 5.1), delivery (Section 5.2), and demand (Section 5.3), along with an assessment of what is known about these strategies’ efficacy. Section 5.4 follows with a discussion of overarching adaptation considerations, including the need to ensure that adaptation measures reduce rather than exacerbate disparities. In this report, resilience is defined as a system's ability to anticipate, prepare for, respond to, recover from, and adapt to a disruption, such as an extreme climate hazard (e.g., a hurricane), with minimum damage to social well‐being, public health, the economy, and the environment. Reliability refers to the system's ability to function consistently during normal conditions. In many cases, adaptation strategies that improve resilience also have the benefit of improving reliability.^194^

### 5.1 Strategies for resilient energy supply

Utilities, the NYISO, FERC, and other agencies charged with safeguarding energy generation and supply systems are engaged in planning and policy‐setting activities that take climate hazards into account. For example, the NYISO has incorporated climate projections into all planning and electric system demand activities. The New York State Public Service Commission required the state's major electric utilities to perform climate change vulnerability studies and develop resilience plans in 2023.^81^ Responding to recent climate‐related events in Texas and the south‐central United States, FERC released a series of 28 recommendations, including nine reliability standards changes.^116^ Of the 28 total recommendations, a technical conference determined that 18 were applicable to NYISO operations (e.g., protecting cold‐weather‐critical components, improving understanding of natural gas fuel supply and reliability risks). The NYISO has implemented, or is implementing, each recommendation applicable in New York State.^195^

Recent changes to electric grid operation and consumer demand have led the NYISO to develop new wholesale market products and examine options for updating existing approaches to valuing resources and performance. NYISO wholesale market products currently include dynamic reserves; capacity accreditation; distributive energy resources participation model; internal controllable lines; transmission constraint modeling; quarterly, 4‐, 10‐, and 20‐year system reliability reviews; balancing intermittency; demand curve reset; and fuel and energy security reports. While these approaches have not been developed for the express purpose of improving grid resilience to climate change impacts, assigning greater value to flexibility and responsiveness—particularly in load pockets—is expected to make the bulk power system more resilient to climate hazards.

Other resilience approaches for energy supply and generation in a changing climate include:

- **Incorporating weather forecasts into planning for energy system operation and emergency response by utilities**. Access to weather data could lead to better forecasting of near‐term events and assist utilities in anticipating possible outages.^196^ Short‐term weather data are available from observational networks within New York State, such as the New York State Mesonet.^197^ The NYSRC recommends that the NYISO use enhanced historical weather data in developing an extreme weather resilience operating plan.^198^ As the grid transitions, models based on realistic weather‐driven simulations will be critical to ensuring resource adequacy and minimizing loss of load.^199^
- **Training and mobilization** of logistics, supply chains, and personnel in advance of and after an event are critical responses to avoid losses and learn from events.
- **Dual and backup fuel requirements**. NYSRC rules require that fossil‐fueled generators in New York City be dual‐fuel, which allows them to burn both natural gas and fuel oil in case supply or cost issues arise with one of these fuels.^200^ On‐site backup fuel storage, including supplies for multiple days of run time, is another resilience strategy adopted by power plants.
- **Redundancy for natural gas compression**. Combustion‐driven compression can use gas provided by the pipeline to continue the circulation of natural gas in the event that electric‐powered technologies are not available due to an outage.^201^
- **Storage capacity** allows grid flexibility so that peak demands can be satisfied. One study explored the adaptive benefits of solar plus storage on grid flexibility, noting that the addition of storage lets the system respond flexibly and provides sufficient reserve power for an operator to maintain the frequency stability of the system, particularly when solar PV generation is lower than forecast.^202^ For natural gas, for example, cold weather in 2014 related to the polar vortex pushed demand to record levels; utilities met the demand by pulling natural gas from storage.^201^

Climate hazard‐specific adaptation measures for the energy supply and generation system are discussed below.

#### 5.1.1 Extreme temperatures

Extreme heat can affect energy supply and generation in several ways. Power generation facilities relying on water cooling can experience a reduction in efficiency, and, therefore, capacity, as air and water temperatures rise. Given that electricity demand tends to increase as temperatures increase, a simultaneous reduction in capacity could lead to a shortfall of supply.^203^ Consideration of both the decrease in efficiency, specific to generation technology, and the increase in load related to changes in temperature can be incorporated into the design of future energy generation capacity in the state.

In extreme cases, elevated water temperatures can force power plants to shut down to avoid violating water discharge temperature limits.^204^ Alternative water supplies for water‐cooled power plants can help alleviate this risk.

Although extremely cold temperatures are expected to continue to occur in the near term, these events will decrease over the course of the century.^38^ Freeze protection and winterization plans for critical components (e.g., wind turbine blades, transmitters, sensing lines, and instrumentation) of energy generation and supply systems will be critical to avoid freeze‐related outages and maintain equipment function during extreme cold.^116^

For natural gas, extreme low temperature causes decreased natural gas production, low pipeline pressure, and other issues. Firm delivery contracts could ensure continued gas delivery during peak demand periods, whereas interruptible supply contracts run the risk of deprioritization and service interruption during these times. Cold events are not new to the New York State system, but systems will need to withstand a larger range of temperatures as the state continues to experience both extreme cold and more extreme heat.

#### 5.1.2 Precipitation

Wind turbines are vulnerable to blade icing because of freezing precipitation. This vulnerability was evident in Texas in 2021 when wind generation was unavailable due to both icing conditions and low wind speeds.^116^ Additionally, turbines can have control systems that automatically shut down in icing conditions. FERC‐recommended winter season reliability assessments should provide clear quantification of risks by assessing and modeling scenarios where low wind and freezing precipitation could occur simultaneously.^116^ Use of hydrophobic coatings on wind turbine blades could minimize the buildup of ice that would threaten operations.

Solar panels are commonly installed in environments exposed to heavy snow and hail. Therefore, the safety rating of PV modules should be observed, including UL 1703, UL 61730–1, and UL 61730–2. Panels that meet these test standards are resilient to water intrusion and hail impacts.^205^

Hydroelectric power generation can also be affected by changes in precipitation. Smaller hydroelectric plants in New York could see a reduction in generation due to seasonal changes in precipitation patterns, including short‐term drought. Technologies such as energy storage can help maintain a consistent power supply.^206^

#### 5.1.3 Sea level rise

Adaptation strategies for electric generation systems located in current and future floodplains include elevating critical equipment (e.g., controls, supervisory control and data acquisition systems) and building temporary or permanent flood barriers. Following Superstorm Sandy, for example, the New York Power Authority implemented infrastructure hardening to provide diversified and redundant communications networks among its sites and ensure connectivity during severe weather events and other emergencies.^207^

Wind turbine designers could incorporate higher elevations into future fixed‐foundation plans in coastal areas to account for the anticipated rise in sea level. When floating foundation designs become technically and economically viable, they will provide another adaptation strategy.

#### 5.1.4 Changes in wind speed

Section 3.1 discusses the possibility that average wind speeds could decrease in the future, though such a change is uncertain. It also notes the potential for more extreme storms. These and any other changes in wind patterns could affect wind turbine operation. Design options such as passive and active aerodynamic “lift modification” devices can support wind turbine operations during periods of extreme high winds and rapidly changing wind conditions. These devices can also improve wind turbine performance in conditions with low wind speeds or reduce loads in conditions with extremely high wind speeds.^208^ Blades are a key design aspect of offshore wind turbines that engineers will need to reassess. One possible solution involves optimizing wind turbine blade design to handle a larger range of incoming wind angles. Light detection and ranging can allow a wind turbine to “look” upwind and “see” what wind loading is coming toward the turbine rotor, allowing it to adjust dynamically to optimize performance. Other design possibilities for inconsistent winds include improving and strengthening pitch and yaw systems to take on higher wind loads, as well as improving wind turbine maintenance efforts.^208^

### 5.2 Strategies for resilient energy delivery

Utilities, regulatory agencies, policymakers, and advocacy organizations recognize that the electricity grid and its system components will have to become more adaptable and resilient as the climate changes. Adapting the grid will require new techniques, including asset mapping and processing of physical events data, creation of vulnerability curves and risk heat maps, evaluation of detailed grid‐impact models, identification of resilience measures and investment optimization, and implementation of emissions reduction interventions.^209^ The NYSRC recognizes that the impact of weather on the power grid has grown with the number and magnitude of extreme weather events, and the organization is studying extreme weather transmission planning criteria.^198^ Additionally, New York State's electric utilities are now required to prepare climate change vulnerability studies to evaluate infrastructure, design specifications, and operational processes that are at risk from climate change^210^, ^211^, ^212^ (refer to the Con Edison Resilience Planning case study for an example). Utilities’ climate resilience plans will be subject to approval by the New York State Public Service Commission and must include measures to improve system hardness, as well as reduce outage time and restoration costs over 5‐, 10‐, and 20‐year time horizons. FERC has initiated a rulemaking for similar studies to be conducted nationwide by all transmission operators.^213^ Other organizations have developed tools to help transmission system utilities evaluate their resilience plans. For example, the North American Transmission Forum offers a Transmission Resiliency Maturity Model that is designed to help transmission system owners and operators identify gaps and prioritize actions to improve the resilience of their systems.^214^

Newer technologies can be deployed to enhance the resilience of the energy delivery system. For example, several technologies were identified in a workshop held in response to Superstorm Sandy to improve grid resilience and reliability. These include energy and distribution management systems, control and data acquisition systems for power lines, advanced meter infrastructure, line sensors and smart relays, outage management systems, and enhanced automated mobile work management systems.^196^ Additional examples of resilience‐enhancing technologies include:

- **Microgrids,** which can offer emergency power options when the primary source of energy is down (refer to the Marcus Garvey Village Microgrid case study).
- **Hybrid renewable (solar and wind) stations with energy storage**. With these systems, wind turbines pick up generation when solar conditions are not ideal, and vice versa. The associated large‐scale grid energy storage offers additional resilience.
- **Redundant communication systems and smart meters** have been employed to improve the speed of grid recovery.^122^
- **Autonomous energy grids,** currently under development, are being designed with control and optimization tools to integrate demand response resources based on machine learning (i.e., without human operators).^215^ Autonomous grids would be particularly useful to send power from the transmission to the distribution level. During an outage when the grid is de‐energized, distributed energy resources would provide power to physically connected local distribution lines and supply electricity until the larger service area's power is restored.

#### 5.2.1 Extreme temperature

The New York City Mayor's Office of Resiliency has published a set of Climate Resiliency Design Guidelines, including resilient design guidance for heat‐resistant infrastructure to reduce the heat island effect.^216^

Vegetation management for transmission lines and rights‐of‐way will require changes that address at least two major issues. First, longer periods of warm weather will lead to insect range expansion, which could lead to disease in danger trees (those with the potential to fall from outside the right‐of‐way into an energized line, causing an outage) and wood poles. The second issue is changing vegetation management within the right‐of‐way, which could include the use of low‐growing cover crops.

Beyond physical assets, operation and maintenance processes may also need to be updated to acknowledge adjustments to standard procedures during heat waves for the safety of employees. Incorporating climate forecasts, monitoring temperature and humidity levels, and providing proper health and safety equipment to field staff is an example of maintaining resilient energy delivery.

#### 5.2.2 Precipitation and flooding

The NYS2100 Commission was tasked with evaluating vulnerabilities to the state's infrastructure system following Superstorm Sandy, Hurricane Irene, and Tropical Storm Lee. The Commission recommended many improvements to energy infrastructure, including strengthening substations against flood damage, elevating distribution transformers, replacing critical distribution wood poles with steel, upgrading existing poles with guy wires, installing excess flow control valves on the natural gas system, and strengthening steam tunnels.^217^ Addressing the Con Edison steam system specifically, in 2013, the Commission recommended flood protection mechanisms such as waterproofing tunnels, improving pump‐out capabilities, constructing higher flood walls around steam generating stations, moving critical equipment to higher elevation, and installing flood pumps.^217^ Con Edison released more detailed measures responding to flooding of the steam system: if more than three‐quarters of an inch of rain is forecasted to fall within 3 h, Con Edison will begin to proactively monitor and address flooding before it can cause a water hammer event. However, if water is pumped from manholes to the city sewer system, the sewer system must be capable of handling the additional volume of water. Isolation valves were installed in strategic locations to reduce the number of customers affected by flooding from future climate events.^129^ Con Edison also addressed the need to provide continuous service to hospitals and responded by installing a new steam main.

In addition to coastal flooding, as described above, electrical transmission infrastructure (including substations, poles, and towers) may be located near riverine floodways. As precipitation increases, floodways may extend onto these assets, leading to water intrusion that causes direct damage, erosion that undermines foundations, or impacts from debris floating in the stream. Relocating assets away from floodways, building redundancy in the electrical system topology, stocking spare components, and building or reinforcing to higher grades are examples of resilience measures that protect against flood hazards.

#### 5.2.3 Sea level rise

New design and upgrade requirements for bulk electric and power systems were established in the wake of Superstorm Sandy. These include installing seawall protection and drainage, as well as constructing bulk electric system and bulk power system equipment at higher elevations to withstand similar storm events and surges.^218^

Utilities and municipalities in New York State's coastal region are also working to establish broad standards that will protect critical infrastructure and energy supply networks from sea level rise. For example, the New York City Climate Resiliency Design Guidelines described above provide specific guidance on flood protection standards for critical infrastructure. The City's guidelines recommend that any structures with useful lives beyond 2040 use the Federal Emergency Management Agency's flood maps to assess the site to determine the height at which structures should be built above the floodplain.^216^

#### 5.2.4 Wind

Design standards for transmission lines and structures will need to be updated to account for higher wind speeds during more severe storms. An example of a response by National Grid to tornado and hurricane damage compounded by impacts from falling trees was the installation of 69‐kilovolt spacer cables and other storm‐resilient equipment, including lightning arresters and polymer insulators.^219^ Similarly, Con Edison redesigned its transmission network to reduce the number of customers losing power when a line is damaged, and reduced feeder segment size.^220^ Poles in storm‐prone areas in Con Edison service territory are required to tolerate wind speeds of up to 110 miles per hour.^220^

### 5.3 Strategies for resilient energy demand

Traditional hardening and adaptation of energy infrastructure (e.g., increasing the security posture of components such as hardware devices, software, network services, and facilities) is used to manage demand during periods of high grid‐stress events and provide backup power in the event of a larger grid or energy system outage. In addition, the emergence of increasingly cost‐effective distributed energy solutions can help manage demand during these periods. For example, distributed solar can reduce the overall need for electricity from the grid, which in turn supports system reliability. When combined with storage and when designed appropriately, distributed energy systems can support the resilience of the energy system by providing backup or reserve power to support individual building operations or to support grid operations. For example, behind‐the‐meter (BTM) PV systems equipped with storage or inverter technology will be capable of supporting customers when there is a power loss. BTM is also a cost‐effective option for storing energy to use during more expensive high‐demand hours.^215^ BTM PV with storage across a number of geographic locations and for different building types is generally capable of providing backup for critical loads (not including heating or cooling), and may offset unavailable grid‐based energy.^221^

Incorporating best‐available climate models into load forecasting models is another strategy that improves energy demand resilience. As established earlier, the increased dependence on electricity coupled with the changing climate affects demand for both electricity and gas. Integrating climate models could enable utilities to proactively prepare for peak demands that may only occur during low‐likelihood climate events by building additional system capacity, redundant systems, or energy storage.

Other approaches to managing and shifting energy demand generally concern the building or user level.^222^, ^223^ These approaches are briefly discussed below and explored further in the Buildings chapter.

### 5.4 Overarching adaptation considerations

#### 5.4.1 The need for coordination

Designing for system resilience to changing societal demands and environmental conditions requires robust diagnosis of conditions and an articulation of interdependencies among key system components at multiple scales.^224^ Figure 6‐5 illustrates these interdependencies for the electric power grid. Coordination, planning, information sharing, and training among energy and telecommunication providers, emergency responders, and local governments will also strengthen the ability to respond speedily and effectively to energy losses from weather‐related and climate‐related events. Details of the planning and operational responses by electric utilities following a power loss are summarized by the National Academies^225^ and the NERC.^226^

**FIGURE 6‐5:**
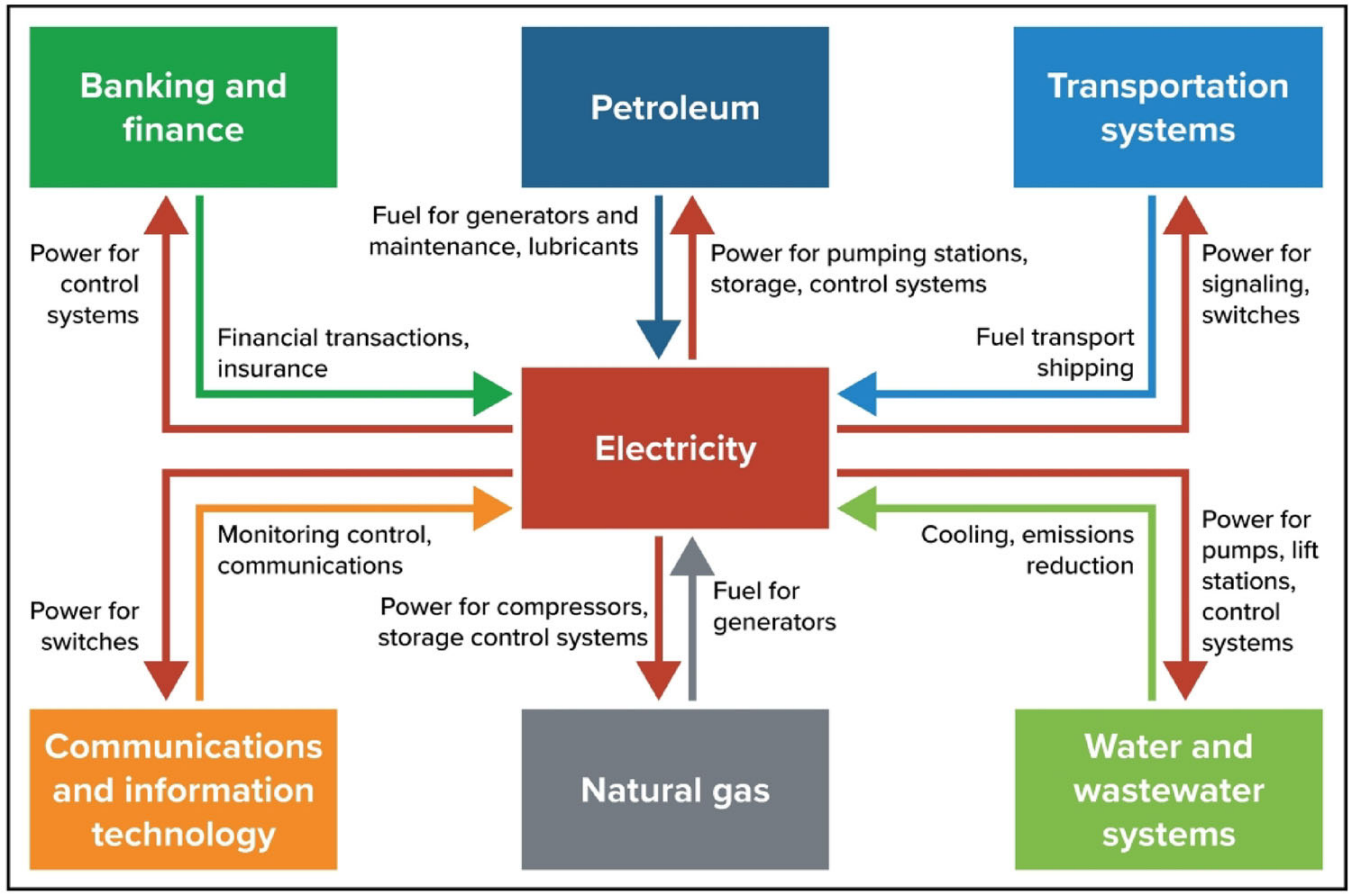
Examples of critical infrastructure interdependence. Figure from U.S. Department of Energy (2017).^122^

#### 5.4.2 Addressing energy injustices

Continuing energy injustice creates greater vulnerability to climate change. There is considerable opportunity for New York State's energy justice efforts to operate in concert with federal government policy and efforts focused on addressing environmental and energy inequality. Much of the federal government's recent attention in this area relates to the Justice40 Initiative (which itself borrowed language from New York's Climate Act) and its assignment of 40% of the benefits from projects related to climate change, renewable energy and energy efficiency, and other areas involving environmental inequality to frontline communities. To advance the initiative and energy justice goals in New York State, support tools for framing the geography of such communities can be employed, including the DOE's Energy Justice Mapping Tool; the White House Council on Environmental Quality's Climate and Economic Justice Screening Tool; and the U.S. Environmental Protection Agency's comprehensive environmental inequality mapping tool, EJScreen. These geographic information system tools can be valuable in aiding researchers, policymakers, governments, and justice‐focused organizations in identifying communities at heightened risk of energy and environmental inequality.

Energy injustices can be addressed through workforce and economic diversification programs, energy assistance and weatherization, expansion of energy technology access, collective action initiatives, and new business development.^227^ However, it is important that these policies be accompanied by a broader set of policies, given that the energy is so fundamental to societal functions and the coming transition may exacerbate pre‐existing disparities, such as energy insecurity. Rather than being prescriptive, the policies suggested are put forth as relevant examples to encourage planning and decision‐making that involve all actors in energy systems.

The potential for job creation associated with the renewable energy sector offers substantial promise for New York State. Workforce and economic diversification programs include workforce training, job development, and regional economic transition for communities that have historically relied on the fossil fuel industry as a main source of employment. Economic diversification programs may create special economic zones that provide incentives for new businesses. As the number of related jobs increases, the potential for unionized labor—and accorded worker protections and benefits—is substantial. Climate Jobs NY, a coalition of labor unions representing more than 2 million members across the state, has a dual goal to combat threats associated with climate change while attacking pervasive income inequality. A just energy transition will also require effort to reduce the demographic disparities in energy sector employment. Ensuring opportunity for Black and Hispanic workers could, among other things, require effort to dissolve historic discriminatory practices prevalent within labor unions.^228^

Energy assistance programs improve the affordability of energy services through subsidies and support for those facing cost challenges. Energy efficiency and weatherization programs, including home audits, provide support for reducing customer demand and cost.

Expansion of technology access programs can build energy‐efficient and renewable energy infrastructure, particularly in marginalized communities. For example, there are considerable untapped renewable energy resources on Tribal lands throughout the United States, which the government and the private sector could partner with Tribal Nations to develop. There are cost challenges, however. For instance, many communities cannot afford solar combined with a storage system. This challenge is especially notable in rural communities, which are more vulnerable to long‐duration power disruptions. Policy support for the adoption of solar‐plus‐storage by households in such circumstances could, therefore, be a highly efficient means of improving the energy security of low‐income rural energy users.

Collective action programs provide community education and awareness about energy issues and the local impacts to engage members of the community in decision‐making processes.

#### 5.4.3 Integration of building retrofits

In addition to adaptation efforts to make energy infrastructure systems more resilient to climate change, end users of energy (particularly buildings) can take adaptation actions to support the resilience of energy services. (The New Paltz Firehouse Adaptations case study describes some of these types of adaptation actions.) For example, energy efficiency is a foundational component of building‐level energy resilience because it reduces the amount of energy that needs to be delivered, results in a smaller overall load for backup energy services, and helps maintain habitable indoor conditions for longer periods of time during power outages. Lawrence Berkley National Laboratory^229^ examined two analyses that looked at how energy efficiency improved reliability and resilience in the California electricity shortages in the early 2000s and the extreme cold snaps in Texas in 2021. Texas's building stock was not designed for extreme cold and has little to no insulation and a high reliance on electric resistance heating. Extreme cold in 2021 increased the Electric Reliability Council of Texas's electricity demand for heating to historic levels. The authors concluded that if homes had efficient building envelopes, the electricity demand could have been reduced by 15 gigawatts—enough to offset the loss of most generators that failed during the event. In California, researchers found that “reliability focused energy efficiency programs implemented for the summer of 2001 (i.e., energy efficiency programs that were specifically designed, modified, or ramped up to address electric system reliability concerns)” saved an estimated 700 megawatt and 1700 gigawatt hours in 2001. Further discussions of building adaptation strategies can be found in the Buildings chapter.

The ability to reduce building‐level demand when needed through actions such as voluntary load reductions and emergency conservation is another strategy used by grid operators to prevent broader energy system outages.^230^

#### 5.4.4 Responses to the urban heat island effect

Academic researchers and city governments in New York State and elsewhere have explored a variety of responses to the urban heat island effect; most of these responses relate to the energy sector.^231^, ^232^, ^233^ Some responses aim to reduce vulnerability or improve resilience in the short term, while others focus on long‐term hardening of the electric grid to extreme heat and changing the fabric of the cityscape to reduce the intensity of the urban heat island effect (e.g., through increased greenspaces or public parks). (As an example of one approach to reduce disproportionate energy burdens, refer to the Urban Heat Islands and Energy Justice case study.) Near‐term responses involve little if any capital investment or infrastructure, and generally entail outreach that identifies vulnerable individuals and provides them with timely access to cooling and other forms of support as needed. Medium‐term responses include setting up cooling centers where energy costs are covered by a public authority and publicizing their availability to nearby residents or providing free air‐conditioning units or heat pumps to low‐income households. Long‐term responses include changes at the level of buildings and infrastructure, including decreasing demand from buildings. The Human Health and Safety chapter provides further discussion of measures to adapt to extreme heat events in urban settings from the perspective of avoiding heat‐related illnesses and deaths.

#### 5.4.5 Strategies for large energy user facilities

For certain large energy users, there are opportunities to generate energy (e.g., heating and electricity) on site^234^ and thereby reduce or minimize dependence on a larger energy system or grid. Wastewater treatment plants are a notable example because they naturally generate large quantities of biogas from the anaerobic digestion process that is used to treat and process sewage. This biogas can be purified and turned into renewable natural gas, which can be used on site as a fuel source in equipment such as a combined heat and power system or in a fuel cell or combined with natural gas in pipelines to help decarbonize the gas system. If the biogas is not used beneficially in this way, it might otherwise be flared. This type of on‐site fuel production and use allows a large industrial site such as a wastewater treatment plant to continue to run even if there is a large grid or gas outage. It also allows these facilities to reduce their demand on the grid (e.g., shift their load to the on‐site power source, rather than continue to draw energy from the grid) to help maintain overall grid reliability and resilience.^234^

National Grid, in collaboration with the New York City Department of Environmental Protection, is building an anaerobic digester gas‐conditioning system that uses biogas from wastewater and food scraps to produce pipeline‐quality biogenic renewable natural gas. Concurrently, the New York City Department of Environmental Protection is undertaking efforts to increase biogas production^40^ in part by diverting food scraps from landfills toward beneficial codigestion at its Newtown Creek wastewater treatment facility. Combined, these projects have the potential to produce enough renewable natural gas to heat more than 5000 homes in New York City.^40^

## 6 LOOKING AHEAD

### 6.1 Opportunities for positive change

As described in Section 3, projected climate impacts during the 21st century will bring warmer temperatures during all seasons and in all regions of New York State. Warming will strain supply, delivery, and demand during increased periods of extreme heat, and warmer temperatures will add to cooling burdens, predominantly in summer. Conversely, warming in winter will reduce heating degree days and, therefore, decrease demand for heating fuel. Space heating is the largest energy expense in the average New York State home^235^; heating costs are, therefore, responsible for the largest share of the energy burden faced by many residents. If climate change reduces heating costs, some residents of the state could benefit. Energy supply and delivery challenges associated with extreme cold temperatures (e.g., demand spikes; infrastructure damage; inhibited distribution of fuel oil via barge, road, or rail due to freezing) could also become less common over time.

Nonetheless, this chapter acknowledges that many energy challenges are likely to worsen with rising temperatures and the increasing frequency and severity of extreme weather events. In terms of dollars alone, all counties in the state are expected to experience an increase in total energy expenditures by the end of the 21st century as a result of climate change (RCP 8.5 scenario)—some by up to 10%.^236^

Coordinating efforts to make the state's energy system cleaner and more resilient (i.e., adapting to climate impacts while also reducing the greenhouse gas emissions that cause climate change) presents many additional opportunities for benefits and cobenefits. Some of the direct resilience benefits of the state's greenhouse gas reduction commitments include:

- **Enhanced building resilience**. Many building envelope improvements that increase energy efficiency (and thus reduce energy consumption) also enhance the ability of the structure to maintain comfortable and safe conditions for inhabitants during extreme weather conditions and power outages. For example, increasing insulation or adding a green roof increases resilience to extreme hot and cold conditions.^237^
- **Availability of backup power in storm events**. An increase in the use of distributed energy resources can increase resilience during extreme events by reducing the number of people who experience outages. In addition, electric vehicles have the potential to serve as a backup battery source for residents.

Additional cobenefits of the state's energy transition include:

- **Job opportunities**. Building a cleaner and more resilient energy system will create new jobs. Workforce development efforts could help make this economic opportunity accessible to all state residents, including rural communities, communities that have been disproportionately harmed by climate change and energy injustice, and demographic groups that are currently underrepresented in clean energy occupations. Implementing these measures will help ensure that the transformation of New York State's energy system is a just transition.
- **Environmental health and air quality improvements**. Development of both centralized and distributed renewable and energy storage resources for resilience and greenhouse gas reduction improves air quality. As clean electricity generation displaces fossil fuel power, greenhouse gas emissions and other pollutants are reduced, yielding air quality benefits to communities and ecosystems located downwind of the emitting resources. The transition away from fossil‐fueled heating and water‐heating equipment in residential and commercial buildings is improving both indoor and outdoor air quality. Fossil‐fueled furnaces, boilers, water heaters, stoves, clothes dryers, and other equipment that combust fuels within buildings impair indoor and outdoor air quality by emitting nitrogen oxides, fine particulates, and other pollutants as well as greenhouse gases. Improving buildings’ energy efficiency and replacing fossil‐fueled equipment with more energy‐efficient electric alternatives could result in meaningful air quality improvements for affected individuals and communities—even if the electricity consumed by the new equipment is still generated by burning fossil fuels in a power plant.
- **Energy costs**. Fossil fuel prices are increasingly volatile, largely because they are traded on global markets. In contrast, a power sector composed of large volumes of renewable resources that have no fuel costs could lead to less volatile energy bills due to the elimination of this driver of variability in energy costs. The presence of distributed resources amplifies this effect. Whether the costs of a clean power sector are lower than, comparable to, or higher than the status quo, they will be more predictable and less likely to create indirect costs that arise from unexpected price changes. Cobenefits from the transition away from fossil‐fueled heating and water‐heating equipment in residential and commercial buildings include lower energy costs to maintain indoor air temperatures and humidity levels.
- **Social justice**. Changes in the energy sector promise a departure from reliance on fossil fuels, which has long been a source of social injustice. The siting and operation of fossil fuel‐based energy infrastructure and facilities have long imposed greater health, physical comfort, and safety burdens on some communities than others. The transition away from reliance on fossil fuels, coupled with a greater awareness of climate‐induced environmental health effects, can help alleviate environmental health burdens and equalize effects across areas. Likely outcomes of such a shift would include replacing emitting power generating facilities near overburdened communities with cleaner facilities; recognizing the impact of energy access and affordability on physical safety and energy demand during extreme heat events; understanding the importance of local land use decisions (e.g., tree cover and access to parks and waterfronts); and improving participation in planning and decision‐making about the energy system.

### 6.2 Emerging topics and research needs

Researchers will continue to study the effects of climate change on energy in New York State. This assessment revealed gaps in knowledge that could be valuable to fill in the years ahead:

- The impact of climate change on natural gas demand will require additional research and monitoring.
- DEFR must be developed and deployed throughout New York State. While not yet commercially available, DEFR will be needed to provide on‐demand power and system stability and reliability.^13^ Research is needed to characterize the gap that is expected to emerge as fossil‐fired generation resources are shuttered, and to identify the most efficient and cost‐effective DEFR technologies that can shrink or fill that gap. The Public Service Commission initiated a proceeding to examine these and other related issues in May 2023 (Order Initiating Process Regarding Zero Emissions Target, Docket 15‐E‐0302, issued May 18, 2023).^47^
- Collaborative work by the electric sector is ongoing to identify data requirements, risk mitigation, and adaptation strategies to build a shared, consistent, and informed approach to maintain system reliability and resilience in a changing climate.^162^
- More research should be conducted on battery storage, including safety/emergency response, performance under various conditions, electric system reliability and resilience, development of long‐duration batteries, sustainability of the battery supply chain, and end‐of‐life disposal.^238^, ^239^
- Additional research is needed to explore the effectiveness of hydrogen production, storage (including long‐duration energy storage technologies), transportation, and end uses. This research includes market research to examine the potential hydrogen economic development opportunities and research into the reduction of nitrogen oxide emissions from hydrogen combustion.
- The impact of climate change on winter weather conditions as they pertain to New York's energy systems will require additional research, modeling, and monitoring. For example, research on the effects of less snowfall and earlier snowmelt on hydropower generation, as well as the impact of warmer weather on residential energy use, is needed.
- Further research is needed on climate hazards with higher uncertainty, such as changes in wind conditions, storm intensity, and changes in precipitation, along with the resultant impacts on the energy system.
- Research is needed to identify actions and policies that reduce the disproportionate distribution of energy system burdens and benefits and facilitate meaningful participation in energy system decisions.

### 6.3 Conclusions

New York State's energy system is vast and complex. It faces considerable challenges from the shifting energy demand that will come with the clean energy transition and the impacts of climate change. Climate change is already affecting energy supply and distribution and is expected to affect renewable energy sources as the state transitions to a carbon‐free energy system. This transition requires continuing efforts to reduce emissions and adapt to climate change; however, there is evidence for optimism (along with uncertainty). As the state's energy system becomes more reliant on renewables and distributed resources, new technologies and grid build‐out will be needed to ensure the system is reliable, safe, resilient, and affordable.

The safe and reliable operation of the energy system will require responsive investments, changes to system design and operations, and continuous assessment of climate impacts and their effects on other sectors that depend on energy resources. The challenge is heightened by uncertainties about both the demand and supply sides of the energy system, including renewables’ sensitivity to the changing climate, and a need for new technologies that are not currently commercially demonstrated to deliver energy when renewable sources are not available.

The energy sector transition must center on energy and environmental justice. Historically overburdened communities suffer disproportionate burdens from the energy system and receive fewer benefits compared to other communities.^240^ Climate change will continue to exacerbate these vulnerabilities unless policy promotes change. In achieving energy justice, communities on the front lines of climate change must play a central role in the renewable energy transition while centering safety, access, and affordability.^241^

## 7 TRACEABLE ACCOUNTS

Traceable accounts examine each key finding in depth. They provide citations that support each assertion and present the authors’ assessment of confidence in each finding.

### 7.1 Key Finding 1

**Climate change is already constraining some sources of energy supply and stressing transmission and distribution infrastructure through extreme heat, changes in precipitation, and increasing storm intensity**. Risks to the energy system, and to other sectors that rely on the energy system, will increase as the climate continues to change. Ongoing assessment of climate impacts, responsive investments, and changes to system design and operation will help ensure the continued safe and reliable operation of the energy system.

#### 7.1.1 Description of evidence

Temperature extremes, increased precipitation, and high winds have direct impacts on electricity generation and delivery, and they are likely to be more pronounced under a changing climate.^38^ Many studies, particularly those conducted after recent hurricanes and tropical storms, identify the need for hardening and adaptive strategies for the sector. Electricity supply from fossil‐based generation decreases with increasing ambient air temperature and as cooling water temperatures increase.^83^, ^85^, ^86^, ^87^, ^88^ A study conducted in various generation plants across the country found a significant correlation between generator failures and extreme temperatures (both cold and hot).^89^ Increases in ambient air temperature are also responsible for the reduced efficiency of solar panels.^90^, ^91^, ^92^, ^93^ For wind technologies, increasing air temperatures may lead to slight declines in air density, decreasing power output.^94^, ^95^ Extremely high (or low) temperatures may damage or reduce the life span of rotating components of the turbines and blades at wind farms.^94^ Increasing air temperatures can also reduce the electrical output of turbines that drive electric generators. Battery‐storage technologies are sensitive to operating temperatures.^96^ Warmer temperatures can reduce battery capacity, efficiency, and lifetime.^97^ While not well studied yet, extreme heat associated with increasing heat waves could require energy storage systems to have internal cooling to prevent increased risks of fires and associated outages at large‐scale battery facilities.

Electrical transmission and distribution systems are sensitive to rising ambient air temperatures.^1^ Transformers, a critical component in distribution and transmission substations, are sensitive to temperatures above their design standard.^123^ High ambient temperatures could lead to planned power interruptions that protect the equipment to prevent damage that would cause longer outages. Extreme heat can cause considerable increases in load, which may exceed the existing installed distribution capacity, require the derating of infrastructure (diminishing its ability to transmit power), increase component failure rates, and limit maintenance or capital work due to workforce restrictions.

Heavy precipitation can lead to flooding that can damage key electricity supply control and operations equipment due to the impacts of scour, corrosion, and ground instability.^83^, ^86^ Transmission line access roads could become inaccessible or washed out, and fixing downed substations and distribution lines could pose access challenges for utility crews. Heavy precipitation and flooding also poses a risk of erosion and landslides that directly affect infrastructure through asset damage, or indirectly affect it by impeding access. Low‐lying generation facilities may be especially vulnerable to heavy precipitation and subsequent runoff. Extreme events (intense rainfall and short‐term drought) may affect infrastructure and operations and maintenance on smaller hydroelectric generation facilities. Increases and decreases in precipitation affect river flows and water levels that govern electricity production by hydroelectric generation.^100^, ^101^ Flooding of electric generation facilities from sea level rise can damage cooling water intake structures, fuel transfer facilities, and electrical substations, which can adversely impact routine operations.^110^ Erosion of underground infrastructure also leads to disruption in generation and delivery.^24^, ^83^ As extreme precipitation increases in frequency and intensity due to climate change,^242^ power generation facilities may need to become resilient to flash flooding to prevent outages. Heavy precipitation in urban areas can flood electrical equipment manholes and cause possible outages. The greatest concern in the future will be flooding and washout of transmission facilities (underground and overhead), including access roads, and icing on overhead lines. Adaptive strategies for system hardening and redundancy will be necessary to ensure a safe and reliable electric grid.

Changes in wind speeds due to climate change are uncertain, but tropical and extra‐tropical cyclones are projected to become more intense.^38^ High winds from storms can damage overhead lines^86^ when debris and trees are blown against the lines, utility poles are blown over, and transmission towers buckle. High winds from hurricanes will adversely impact energy delivery infrastructure, mostly in coastal areas of the state.

Extremely cold temperatures can affect the natural gas supply system by freezing fluid handling equipment, transmitters, sensing lines, valves, and inlet air systems; however, widespread natural gas supply disruptions in New York due to extreme cold temperatures have been infrequent.^116^ Heavy precipitation can lead to water infiltration into underground gas pipelines and tunnels, which can cause service interruptions.^118^ Increased variability in winter temperatures^133^ could pose additional risks to the natural gas delivery system. Freeze‐thaw cycles can result in frost heaves, causing pipeline movement or undermining that could increase pipe stress and fracture.^133^

Risks to liquid fuels distribution infrastructure were revealed following Superstorm Sandy, when gasoline could not be dispensed after power failures disabled pumps and other equipment at filling stations.^243^ Flooding from coastal storms and sea level rise also poses a major risk to fuel terminals located on the coasts, due to physical surge impacts and saltwater impacts upon a range of critical equipment.^24^, ^25^, ^110^ Flooding can cause soil erosion, which can undermine pipeline systems for liquid fuels delivery, as well as fuel storage tanks and foundations.

The energy sector's vulnerabilities to climate change impacts can cause disruptions throughout the state's economy.^154^, ^155^ Effects of the extended power failure caused by Superstorm Sandy in 2012 illustrate these cascading impacts. For example, equipment was disabled or damaged in tunnels where pumps failed and in buildings where electrically powered devices would have protected against inundation.^156^ Because gasoline could not be dispensed after power failures disabled pumps and other equipment at filling stations, gasoline was rationed, and long lines at filling stations formed.^25^ Electrification of the buildings and transportation sectors is likely to make systems more interdependent, altering how energy sector exposure to hazards can translate into vulnerabilities in other sectors.^159^, ^160^

The safe and reliable operation of the electric energy system is the primary obligation of the NYISO, which constantly assesses the system's operation and the impacts of climate change.^13^, ^195^, ^244^

#### 7.1.2 New information and remaining uncertainties

The economy‐wide transformation to a carbon‐free energy system has major implications for the resilience and reliability of the energy sector in terms of how end use patterns and the supply‐side mix will change. However, the pace of that transition and the mix of energy resources that will support the electric grid remain unknown. Various studies conducted in New York, such as the Scoping Plan published in 2022 as well as other state and private‐public studies,^16^, ^39^, ^40^, ^148^ provide examples of how to achieve both the economy‐wide greenhouse gas reduction targets and the clean electricity goals.

More information is needed to understand how temperature swings (or “extreme” short‐term variability in temperatures) affect natural gas delivery infrastructure, such as compressor stations. More research is needed to better understand how prolonged cold snaps or polar vortex‐like events, especially in the near‐term, could impact energy delivery infrastructure. There is also a lack of information about how climate conditions could affect potential future energy delivery infrastructure that evolves or develops during the timeframe of this assessment, such as hydrogen fueling stations or pipelines.

#### 7.1.3 Assessment of confidence based on evidence

There is **high** confidence that climate hazards affect the generation and delivery of energy now and will continue to do so in the future, given the many post‐event system impact assessments and forecast modeling of energy infrastructure. There is also **high** confidence that the state's energy system will change over the next 20 years as it adapts to new technologies, policy advances, and climatic changes. However, there is **medium** confidence in the rate of change to a fully renewable energy system and in the technology resource mix for energy generation.

### 7.2 Key Finding 2

**Patterns of energy demand are shifting due to climate change and are expected to continue evolving over the coming decades**. Altered patterns of energy demand can strain energy supply and delivery (especially during peak periods) and may lead to infrastructure failure and energy price increases. Ensuring a reliable energy supply and delivery will require diverse solutions such as investments in new energy infrastructure, hardening of current energy infrastructure, new business models, and demand‐side management programs.

#### 7.2.1 Description of evidence

The evidence that climate change is altering patterns of energy demand through changing temperatures comes from studies of near‐term climate scenario futures for New York State and New York City, including the Con Edison Climate Change Vulnerability Study and the NYISO Climate Change Phase I Study. Both studies project increased energy demands due to increases in average and peak temperatures from projected climate change impacts.^128^, ^145^ Various other sources show evidence of increased energy use pattern changes associated with increasing temperatures.^112^, ^141^, ^142^, ^143^ One metastudy^112^ reviewed studies focused on the potential impact of climate change on the bulk power system and found that electric demand would increase through the end of this century. A NYSERDA study on the impact of climate change on the energy system, using different methodologies from the NYISO report, also found that higher temperatures will increase cooling demand and decrease heating demand over time.^90^ Heat waves, which are expected to become longer, more frequent, and more intense across New York State,^38^ increase electricity demand (this demand is driven primarily by cooling needs for building residents and occupants as well as for critical equipment). Historically, the “degree‐days” methodology is used for estimating the heating and cooling energy demand of buildings.^140^ The greater the number of heating and cooling days, the greater the potential demand for energy. Accordingly, many of these same studies show a decrease in energy demand due to a decline in heating degree days associated with the impacts of climate change.

Con Edison has projected that its natural gas and steam sectors could both experience substantial decreases in winter energy sales for heating degree days: a 33% decrease by 2050, and a 49% decrease by 2080. The same study also suggests a potential increase in natural gas and steam use for cooling in the summer.^117^ These figures are driven solely by climate change, and not by any other changes in demand due to electrification or other factors. A decline in heating degree days will reduce heating fuel demand.^153^ This reduction will apply to fuel oil and any other fuels used for heating, as well as to district steam systems. A NYSERDA study on the impact of climate change on the energy system found that warming temperatures would reduce the demand for fuels for heating over time.^90^

Newer technologies will be deployed to respond to changes in energy demand while enhancing resilience. These include energy and distribution management systems, control and data acquisition systems for power lines, advanced metering infrastructure, line sensors and smart relays, outage management systems, enhanced automated mobile work management systems,^196^ microgrids, battery storage, redundant communications systems,^122^ and autonomous energy grids.^215^

#### 7.2.2 New information and remaining uncertainties

The effects of acute extreme events (e.g., increasing storm intensity and sea level rise, increased precipitation, and extreme precipitation) on energy demand are less clear and less studied than the effects of temperature. While climate projections for New York State show a general warming trend that will lead to more cooling degree days and fewer heating degree days annually, uncertainty remains regarding the impact of climate change on severe winter weather and peak day demand, especially in the near term; cold days will still occur, and some winters will be colder than others.^151^, ^152^ Nonclimate factors such as electrification will also affect demand. To the extent that the state sees widespread electrification of heating, electric demand could be increasingly sensitive to heating degree days, with demand potentially spiking during periods of extreme cold. In general, despite overall warming trends, there continues to be a need to plan for extreme cold events.

The impact of climate change on gas demand over the course of the 21st century statewide will require additional research and monitoring. It is currently necessary to make certain assumptions about the composition of the energy system in the years ahead to determine the impact of climate change on the demand for natural gas. If the use of natural gas diminishes over time, both in space heating and in power generation, the need for gas supplies from out of state will also diminish, and any potential impacts of climate change on gas supply infrastructure will be reduced. If the natural gas supply system remains similar to its current construct, it is unclear whether the reduction in heating degree days will offset the increase in cooling degree days such that New York's current gas system will be adequate to meet future demand in a changed climate.

#### 7.2.3 Assessment of confidence based on evidence

There is **high** confidence that climate change affects energy demand, in particular through changes in heating and cooling degree days as well as increased frequency and duration of heat waves. There is less confidence (**low** to **medium**) in understanding exactly how other types of climate risk (e.g., extreme events) will affect demand.

### 7.3 Key Finding 3

**As New York State's energy system becomes more electrified and more reliant on emission‐free electricity supply sources, new approaches will be needed to adapt to climate change and ensure the system is flexible, safe, resilient, and cost‐effective**. This challenge is heightened by uncertainties on both the demand and supply sides of the electricity system, including renewables’ sensitivity to climate change and the need for new technologies, infrastructure, and operations. Solutions such as demand‐side behavior changes, operational adjustments, investments in system capabilities and capacities, modified business models, and new technologies that are attuned to the changing energy system can moderate the effects of uncertainty due to climate change.

#### 7.3.1 Description of evidence

The electricity supply in New York is expected to expand through the inclusion of nonemitting resources such as wind and solar, offshore wind, and battery storage. The Climate Act mandates additions of nonemitting resources to achieve a 70% renewable portfolio standard by 2030 and a 100% zero‐emission electric grid by 2040, as well as specific resource quantities for offshore wind and distributed solar—9000 megawatts of offshore wind by 2035 and 6000 megawatts of distributed solar.^7^ Over the coming years, the transmission system will expand to accommodate the addition of renewable energy and increased electrification and to address existing constraints that currently affect power flows within the state.^16^ Baseline electricity energy demand is projected to increase by up to 31% between 2021 and 2050.^146^ Meeting this demand, complying with current policy, and achieving a cost‐effective decarbonized electric grid will require approximately 104,000 gigawatt hours of annual renewable energy with 12 gigawatts of energy storage by 2040, and 198,000 gigawatt hours of annual renewable energy with more than 17 gigawatts of storage by 2050. The addition of these resources would offer critical benefits in terms of grid reliability and integration of renewable generation.

Given these projections, climate adaptation for current and future energy supply and transmission components is critical.^50^ Adaptation measures are outlined in Section 5. Given changes in the energy system, energy supply adaptation should include increasing integration across technical, economic, regulatory, and social dimensions in order to increase reliability and performance, reduce cost, and minimize environmental impacts.^194^

#### 7.3.2 New information and remaining uncertainties

There is considerable future uncertainty in the New York State electric power grid. The system is set to change dramatically over the next 20 years, including the addition of new and innovative dispatchable emissions‐free resource technologies. By 2040, the total installed generation capacity that will be needed to reach policy objectives is between 111 and 124 gigawatts, including approximately 95 gigawatts of new emissions‐free resource generation.^13^ Unfortunately, while renewable generation has increased in New York State with decentralization, it has not kept pace with retiring fossil‐fueled resources. Also, fully achieving the emission‐free grid target by 2040 will require not only more renewable resources, but also transmission of greater amounts of clean power.^13^

Distributed renewable energy systems face several challenges such as the intermittency of renewables, uncertainties with weather and load forecasts, and lack of comprehensive control systems.^42^ Renewable power intermittency imposes even larger uncertainties at micro‐scales, as both under and over generation of power can have a considerable negative impact on reliability.^43^ Moreover, many of the technologies necessary for reliable 100% renewable electrical grids, such as thermal energy storage,^10^ long‐duration energy storage,^11^ and hydrogen storage,^12^ are not commercially available at scale.

While climate change will have an impact on the energy system, the exact timing and magnitude of future exposure is uncertain. A flexible and adaptive approach is necessary to manage risks. Adapting the grid will require new techniques including asset mapping and processing of physical events data, creation of vulnerability curves and risk heat maps, evaluation of detailed grid‐impact models, identification of resilience measures and investment optimization, and implementation of emissions reduction interventions.^209^

Demand‐side management,^222^, ^223^ modified business models, operational adjustments, investments in system capabilities and capacities, and new technologies^14^, ^15^, ^112^ will also need to be attuned to the changing energy system. However, these strategies, models, operational adjustments, and investments have yet to be developed.

#### 7.3.3 Assessment of confidence based on evidence

There is **high** confidence that New York State's energy system will change rapidly over the next 20 years in adapting to new technologies, policy advances, and climate change. There is also **high** confidence that new approaches, technologies, and investments will be needed to provide the energy necessary for reliable, safe, and clean energy.

### 7.4 Key Finding 4

**Climate change could result in unequal impacts across communities due to existing inequalities and burdens in New York State's energy system, especially as the system evolves**. People of color and low‐income households are already more likely than other communities to experience challenges cooling their homes in hot weather and are more vulnerable to power outages during extreme weather events, for example. Responding to climate change gives New York an opportunity to work toward a more just and resilient energy system in which all racial, ethnic, Indigenous, and socioeconomic groups have equitable benefits of clean and resilient energy infrastructure, affordable energy, and associated jobs. Actions such as meaningful community involvement in decision‐making processes can help overcome local barriers and disparities while developing equitable policy choices in the face of a changing climate and an evolving energy system.

#### 7.4.1 Description of evidence

Evidence reveals that both the benefits and burdens of energy systems, as well as the adverse impacts of climate change, are experienced differently across communities, and that people of color and low‐income households experience a greater share of environmental burdens than other communities^52^ while benefiting less than other groups.^53^, ^54^, ^55^, ^56^, ^57^

Poverty and race are critical to energy burdens. Approximately 14% of all New York State residents live in poverty, with higher shares across the state's cities. In Buffalo, Rochester, and Syracuse, for instance, 3 out of every 10 residents live in poverty.^245^ Both urban and rural low‐income households spend roughly three times as much of their income on energy compared to high‐income households.^55^, ^170^ Black and Hispanic households bear disproportionately larger energy burdens and pay a larger share of income for energy services than most other groups.^55^, ^73^, ^185^ Indigenous Peoples have the lowest incomes among population groups in New York State.^245^ Generally, households in Indigenous communities have an average energy burden that is 45% higher than non‐Hispanic white households nationwide.^73^, ^171^

Research has found that low‐income households and those facing energy insecurity often face acute vulnerability to climate impacts within the energy sector. These vulnerabilities include economic challenges posed by the expense of heating and cooling homes during temperature extremes, which can result in increased morbidity and mortality as well as food insecurity^183^ and heightened vulnerability during and after extreme weather events.^184^ Low‐income households and communities of color are more vulnerable to power outages than their neighbors in New York City during the warm months of the year^185^; such power outages could become more common in a changing climate. These communities also face risks of utility disconnections and disaster‐related discontinuation of energy services.^176^ Preliminary evidence from a New York City‐based study shows that power outages pose substantial threats to communities that are located in urban heat islands and disproportionately low‐income, especially communities of color, and that air conditioning equity issues could worsen with the advance of climate change.^186^

Not all groups are experiencing the benefits of the current energy system equally either. For example, energy injustices often manifest in low access to clean energy jobs. One study suggested that the “transition to a clean energy economy could help address economic inclusion challenges from the national to the local level. However, the current roster of workers in related occupations is far from inclusive—suggesting the existence of distinct barriers to access that require additional attention and action.”^60^ This study finds that “the clean energy economy workforce is older, dominated by male workers, and lacks racial diversity when compared to all occupations nationally.”^60^ The U.S. Bureau of Labor Statistics projects that solar PV installers and wind turbine service technicians will rank among the fastest‐growing occupations in the United States over the next decade.^61^ In New York State, these and other jobs in the clean energy sector provide more job opportunities than fossil fuel industries^62^ but are often unavailable to certain communities.

Energy injustices can be addressed through workforce and economic diversification programs, energy assistance and weatherization, expansion of energy technology access, collective action initiatives, and new business development.^227^

Notwithstanding the potential to provide climate and environmental benefits, renewable energy transitions are neither inherently just nor democratic. Policies that support marginalized populations must, therefore, be at the vanguard of renewable energy and climate policy^164^ in order for New York State to meet statutory obligations related to the Climate Act. In the absence of targeted energy justice policy and practice, with vulnerable households and communities as a focal point, the potential benefits of a renewable energy transition (including related jobs, which are expected to be among the fastest growing occupations across the nation over the next decade)^61^ will continue to be inaccessible to historically overburdened populations.^57^, ^240^ Energy governance and related decision‐making—like other aspects of public planning—has historically not been inclusive of all communities.^64^ Mitigating the effects of income inequality and energy burdens will require more meaningful involvement and participation by members of frontline communities. Reduction of harms must address the political marginalization of groups as a cause of social vulnerability.^241^

#### 7.4.2 New information and remaining uncertainties

Uncertainties loom over multiple factors related to the accessibility of energy resources. These factors include the costs of energy and related technologies, the availability of subsidies and other policy supports that can aid household and municipal adoption of energy technologies, investment in weatherization, and climate adaptation planning. Migration both into and within New York State is also uncertain and likely to influence energy‐related outcomes. Cities in Central and Western New York, for instance, are projected to become climate hubs that see their populations grow; other regions, particularly along the state's coast, could experience climate flight.

#### 7.4.3 Assessment of confidence based on evidence

The available evidence supports with **very high** confidence that pervasive socioeconomic inequality has major implications for the distribution of energy system goods and harms, the ability to participate in planning and decision‐making about the future of energy in the state, and vulnerability to climate change. For New York State to meet its renewable energy goals—which have centered on environmental and energy justice—it will be necessary to increase energy affordability and access to energy technologies and jobs. Failure to do so will negatively impact the state's ability to meet renewable energy standards codified by law.

## AUTHOR CONTRIBUTIONS

P.J.M.: Drafting, revising, and editing the manuscript; manuscript compilation and review; general supervision. S.M: Drafting, revising, and editing the manuscript; manuscript compilation and review; general supervision. S.D.: Drafting and revising the manuscript. P.C.: Drafting and revising the manuscript. J.F.: Drafting and revising the manuscript. M.G.: Drafting and revising the manuscript. J.G.: Drafting and revising the manuscript. J.P.: Drafting and revising the manuscript. P.S.: Drafting and revising the manuscript. W.V.S.: Drafting and revising the manuscript. L.T.: Drafting and revising the manuscript. K.W.: Drafting and revising the manuscript.

## COMPETING INTERESTS

The authors declare no competing interests.

### PEER REVIEW

The peer review history for this article is available at: https://publons.com/publon/10.1111/nyas.15191

## References

[1] Rosenzweig, C. , Solecki, W. , DeGaetano, A. , O'Grady, M. , Hassol, S. , & Grabhorn, P. (2011). Responding to climate change in New York State: The ClimAID integrated assessment for effective climate change adaptation. New York State Energy Research and Development Authority. https://www.nyserda.ny.gov/About/Publications/Energy‐Analysis‐Reports‐and‐Studies/Environmental‐Research‐and‐Development‐Technical‐Reports/Response‐to‐Climate‐Change‐in‐New‐York

[2] U.S. Energy Information Administration . (2022). Profile analysis. New York State Profile and Energy Estimates. https://www.eia.gov/state/analysis.php?sid=NY

[3] New York State Energy Research and Development Authority . (2023). Patterns and trends: New York State energy profile . https://www.nyserda.ny.gov/About/Publications/Energy‐Analysis‐Reports‐and‐Studies/Patterns‐and‐Trends

[4] U.S. Energy Information Administration . (n.d.). New York Independent System Operator (NYIS) electricity overview. Hourly Electric Grid Monitor. https://www.eia.gov/electricity/gridmonitor/dashboard/electric_overview/balancing_authority/NYIS

[5] Industrial Economics, Incorporated . (2023). The benefits, costs, and economic impacts of undergrounding New York's electric grid. Prepared for New York State Department of Public Service and New York State Energy Research and Development Authority. https://dps.ny.gov/system/files/documents/2023/09/final‐report‐ny‐undergrounding‐2023‐06‐27.pdf

[6] New York Independent System Operator . (2023). Power trends 2023: A balanced approach to a clean and reliable grid . https://www.nyiso.com/documents/20142/2223020/2023‐Power‐Trends.pdf

[7] Climate Leadership and Community Protection Act, S6599, New York State Senate, 2019–2020 Legislative Session . (2019). https://www.nysenate.gov/legislation/bills/2019/S6599

[8] Hochul, K. (2022). State of the state 2022: A new era for New York . https://www.governor.ny.gov/sites/default/files/2022‐01/2022StateoftheStateBook.pdf

[9] New York Power Authority & Canal Corporation . (2021). 2021 sustainability report . https://www.nypa.gov/‐/media/nypa/documents/document‐library/esg‐sustainability/nypa‐sustainability‐report‐2021.pdf

[10] Alva, G. , Lin, Y. , & Fang, G. (2018). An overview of thermal energy storage systems. Energy, 144, 341–378. 10.1016/j.energy.2017.12.037

[11] Chen, H. , Cong, T. N. , Yang, W. , Tan, C. , Li, Y. , & Ding, Y. (2009). Progress in electrical energy storage system: A critical review. Progress in Natural Science, 19(3), 291–312. 10.1016/j.pnsc.2008.07.014

[12] Zhang, F. , Zhao, P. , Niu, M. , & Maddy, J. (2016). The survey of key technologies in hydrogen energy storage. International Journal of Hydrogen Energy, 41(33), 14535–14552. 10.1016/j.ijhydene.2016.05.293

[13] New York Independent System Operator . (2022). 2021–2040 system & resource outlook. Presentation Before the Environmental Advisory Council. https://www.nyiso.com/documents/20142/33223817/EAC‐20221005‐System‐Resource‐Outlook‐presentation.pdf

[14] Nik, V. M. , Perera, A. T. D. , & Chen, D. (2021). Towards climate resilient urban energy systems: A review. National Science Review, 8(3), nwaa134. 10.1093/nsr/nwaa134 34691589 PMC8288381

[15] Nik, V. M. , & Perera, A. T. D. (2020). The importance of developing climate‐resilient pathways for energy transition and climate change adaptation. One Earth, 3(4), 423–424. 10.1016/j.oneear.2020.09.013

[16] New York State Department of Public Service & New York State Energy Research and Development Authority . (2021). Initial report on the New York power grid study . https://www.nyserda.ny.gov/About/Publications/Research‐and‐Development‐Technical‐Reports/Electric‐Power‐Transmission‐and‐Distribution‐Reports/Electric‐Power‐Transmission‐and‐Distribution‐Reports—Archive/New‐York‐Power‐Grid‐Study

[17] New York State Department of Public Service . (2022). Electric and gas utility service territory by county . https://dps.ny.gov/system/files/documents/2022/10/nys‐electric‐and‐gas‐utilities‐by‐county.pdf

[18] U.S. Department of Energy . (2021). State of New York: Energy sector risk profile. Office of Cybersecurity, Energy Security, and Emergency Response. https://www.energy.gov/sites/default/files/2021‐09/New%20York%20Energy%20Sector%20Risk%20Profile.pdf

[19] New York State Department of Public Service . (n.d.). Completed annual reports of regulated utilities . https://dps.ny.gov/completed‐annual‐reports‐regulated‐utilities

[20] U.S. Department of Transportation . (n.d.). Gas distribution, gas gathering, gas transmission, hazardous liquids, liquefied natural gas (LNG), and underground natural gas storage (UNGS) annual report data. Pipeline and Hazardous Materials Safety Administration. https://www.phmsa.dot.gov/data‐and‐statistics/pipeline/gas‐distribution‐gas‐gathering‐gas‐transmission‐hazardous‐liquids

[21] New York State Energy Planning Board . (2015). Volume 2: Sources. In *2015 New York State energy plan*. https://energyplan.ny.gov/‐/media/Project/EnergyPlan/files/2014stateenergyplan‐documents/2015‐nysep‐vol2‐sources.pdf

[22] Proceeding on motion of the commission assessing implementation of and compliance with the requirements and targets of the Climate Leadership and Community Protection Act, 22‐M‐0149 . (2022). https://documents.dps.ny.gov/public/MatterManagement/CaseMaster.aspx?Mattercaseno=22‐00570

[23] Proceeding on motion of the commission in regard to gas planning procedures, 20‐G‐0131 . (2020). https://documents.dps.ny.gov/public/MatterManagement/CaseMaster.aspx?MatterCaseNo=20‐g‐0131

[24] ICF International . (2014). New York State petroleum terminal resiliency assessment (NYSERDA Contract 30186). New York State Energy Research and Development Authority. https://www.nyserda.ny.gov/‐/media/Project/Nyserda/Files/EDPPP/Energy‐Prices/Energy‐Statistics/NYS‐terminal‐resiliency‐assessment‐final‐report.pdf

[25] Hallman, R. M. , & Wei, K. (2016). Improving regional situational awareness during fuel emergencies in the New York tri‐state area: Lessons from Superstorm Sandy. Columbia University School of International and Public Affairs, Center on Global Energy Policy. https://www.energypolicy.columbia.edu/publications/improving‐regional‐situational‐awareness‐during‐fuel‐emergencies‐new‐york‐tri‐state‐areas‐lessons

[26] An Act to Amend the Environmental Conservation Law, in Relation to Bioheating Fuel Requirements, A07114, New York State Assembly, 2023–2024 Regular Sessions . (2023). https://www.nyassembly.gov/leg/?default_fld=&leg_video=&bn=A07114&term=&Summary=Y

[27] Assembly Bill 7114, New York State Assembly, 2023–2024 Legislative Session . (2023). https://legiscan.com/NY/bill/A07114/2023

[28] Young, M. (2021). How the New York City steam system works . Untapped New York. https://untappedcities.com/2021/07/09/new‐york‐city‐steam‐system/

[29] Consolidated Edison Co. of New York, Inc . (2021). Long‐range plan: Our district steam system . https://www.coned.com/‐/media/files/coned/documents/our‐energy‐future/our‐energy‐projects/steam‐long‐range‐plan.pdf

[30] Oliker, I. (2022). Lessons from Buffalo: Evaluating decades of district heating. HPAC Engineering, https://www.hpac.com/heating/article/21212992/lessons‐from‐buffalo‐evaluating‐decades‐of‐district‐heating

[31] Jamestown Board of Public Utilities . (n.d.). District heat . https://www.jamestownbpu.com/260/District‐Heat

[32] Rochester District Heating Cooperative . (n.d.). District thermal energy . https://rdhc.org/district‐thermal‐energy/

[33] Oliker , I. , Morris, P. , Totorici, III, R. , & Jerabek, E. (2021). Development and operation of a district energy system in Schenectady, New York. Engineered Systems, 38(2). https://digitaledition.esmagazine.com/february‐2021/oliker‐feature/

[34] New York State Energy Research and Development Authority . (n.d.). Hydrogen . https://www.nyserda.ny.gov/All‐Programs/Hydrogen

[35] New York State Energy Research and Development Authority . (2022). Patterns and trends: New York State energy profiles, 2004–2018 [Final report]. https://www.nyserda.ny.gov/‐/media/Project/Nyserda/Files/Publications/Energy‐Analysis/2004‐2018‐PattensAndTrends‐EEA.pdf

[36] U.S. Energy Information Administration . (2022). New York State energy profile . https://www.eia.gov/state/print.php?sid=NY

[37] U.S. Energy Information Administration . (2021). U.S. energy atlas . https://atlas.eia.gov/

[38] Lamie , C. , Bader, D. , Graziano, K. , Horton, R. , John, K. , O'Hern, N. , Spungin, S. , & Stevens, A. (2024). New York State Climate Impacts Assessment Chapter 02: New York State's Changing Climate. Annals of the New York Academy of Sciences, 1542, 91–145. 10.1111/nyas.15240 PMC11668500 39652367

[39] Energy and Environmental Economics, Inc . (2020). Pathways to deep decarbonization in New York State . https://www.nyserda.ny.gov/‐/media/Project/Nyserda/Files/EDPPP/Energy‐Prices/Energy‐Statistics/2020‐06‐24‐NYS‐Decarbonization‐Pathways‐Report.pdf

[40] New York City Mayor's Office of Sustainability . (2021). Pathways to carbon‐neutral NYC: Modernize, reimagine, reach . https://www.nyc.gov/assets/sustainability/downloads/pdf/publications/Carbon‐Neutral‐NYC.pdf

[41] New York State Energy Research and Development Authority & New York State Department of Environmental Conservation . (2021). Appendix G: Integration analysis technical supplement. In *New York State Climate Action Council draft scoping plan*. https://climate.ny.gov/‐/media/project/climate/files/Draft‐Scoping‐Plan‐Appendix‐G‐Integration‐Analysis‐Technical‐Supplement.pdf

[42] Safaei Pirooz, A. A. , Sanjari, M. J. , Kim, Y.‐J. , Moore, S. , Turner, R. , Weaver, W. W. , Srinivasan, D. , Guerrero, J. M. , & Shahidehpour, M. (2023). Adaptation of high spatio‐temporal resolution weather/load forecast in real‐world distributed energy‐system operation. Energies, 16(8), 3477. 10.3390/en16083477

[43] Sechilariu, M. , Wang, B. , & Locment, F. (2013). Building integrated photovoltaic system with energy storage and smart grid communication. IEEE Transactions on Industrial Electronics, 60(4), 1607–1618. 10.1109/TIE.2012.2222852

[44] New York Independent System Operator . (2022). 2021–2040 system & resource outlook . https://www.nyiso.com/documents/20142/32663964/2021‐2040_System_Resource_Outlook_Report_DRAFT_v15_ESPWG_Clean.pdf

[45] Hibbard, P. J. , Wu, C. , Krovetz, H. , Farrell, T. , & Landry, J. (2020). Climate change impact and resilience study—Phase II: An assessment of climate change impacts on power system reliability in New York State [Final report]. https://www.nyiso.com/documents/20142/16884550/NYISO‐Climate‐Impact‐Study‐Phase‐2‐Report.pdf

[46] New York Independent System Operator . (2023). Comments of the New York Independent System Operator, Inc., on the order initiating process regarding zero emissions target . https://www.nyiso.com/documents/20142/35671756/20230816‐NYISO‐Cmmnts‐Zero‐Emissions‐Target‐Cmplt.pdf

[47] Order initiating process regarding zero emissions target, 15‐E‐0302 . (2023). https://documents.dps.ny.gov/public/Common/ViewDoc.aspx?DocRefId=00E12F88‐0000‐C914‐BA3F‐E14BF4BA3762

[48] New York State Energy Research and Development Authority . (2023). Seven states in NE Regional Clean Hydrogen Hub announce DOE proposal for funding and designation as a national hub . https://www.nyserda.ny.gov/en/About/Newsroom/2023‐Announcements/2023‐4‐7‐Seven‐States‐in‐Northeast‐Regional‐Clean‐Hydrogen‐Hub

[49] Proceeding on motion of the commission to implement transmission planning pursuant to the Accelerated Renewable Energy Growth and Community Benefit Act, 20‐E‐0197 . (2021). https://documents.dps.ny.gov/public/MatterManagement/MatterFilingItem.aspx?FilingSeq=259215&MatterSeq=62480

[50] New York State Energy Research and Development Authority . (2021). New York power grid study . https://www.nyserda.ny.gov/About/Publications/Energy‐Analysis‐Reports‐and‐Studies/Electric‐Power‐Transmission‐and‐Distribution‐Reports/Electric‐Power‐Transmission‐and‐Distribution‐Reports—Archive/New‐York‐Power‐Grid‐Study

[51] Brun, K. (2018). The U.S. natural gas compression infrastructure: Opportunities for efficiency improvements. University Turbine Systems Research Symposium. https://netl.doe.gov/sites/default/files/netl‐file/Brun.pdf

[52] Banzhaf, S. , Ma, L. , & Timmins, C. (2019). Environmental justice: The economics of race, place, and pollution. Journal of Economic Perspectives, 33(1), 185–208. 10.1257/jep.33.1.185 30707005

[53] Mohai, P. , Pellow, D. , & Roberts, J. T. (2009). Environmental justice. Annual Review of Environment and Resources, 34(1), 405–430. 10.1146/annurev-environ-082508-094348

[54] Brown, M. A. , Soni, A. , Lapsa, M. V. , Southworth, K. , & Cox, M. (2020). High energy burden and low‐income energy affordability: Conclusions from a literature review. Progress in Energy, 2(4), 042003. 10.1088/2516-1083/abb954

[55] Drehobl, A. , & Ross, L. (2016). Lifting the high energy burden in America's largest cities: How energy efficiency can improve low income and underserved communities. American Council for an Energy‐Efficient Economy. https://www.aceee.org/research‐report/u1602

[56] Jenkins, K. , McCauley, D. , Heffron, R. , Stephan, H. , & Rehner, R. (2016). Energy justice: A conceptual review. Energy Research & Social Science, 11, 174–182. 10.1016/j.erss.2015.10.004

[57] Carley, S. , & Konisky, D. M. (2020). The justice and equity implications of the clean energy transition. Nature Energy, 5(8), 569–577. 10.1038/s41560-020-0641-6

[58] Initiative for Energy Justice . (2019). Section 1—Defining energy justice: Connections to environmental justice, climate justice, and the just transition. The Energy Justice Workbook. https://iejusa.org/section‐1‐defining‐energy‐justice/

[59] Dooley, K. , Holz, C. , Kartha, S. , Klinsky, S. , Roberts, J. T. , Shue, H. , Winkler, H. , Athanasiou, T. , Caney, S. , Cripps, E. , Dubash, N. K. , Hall, G. , Harris, P. G. , Lahn, B. , Moellendorf, D. , Müller, B. , Sagar, A. , & Singer, P. (2021). Ethical choices behind quantifications of fair contributions under the Paris Agreement. Nature Climate Change, 11(4), 300–305. 10.1038/s41558-021-01015-8

[60] Muro, M. , Tomer, A. , Shivaram, R. , & Kane, J. W. (2019). Advancing inclusion through clean energy jobs. Brookings Institution. https://www.brookings.edu/articles/advancing‐inclusion‐through‐clean‐energy‐jobs/

[61] U.S. Bureau of Labor Statistics . (2022). Fastest growing occupations. Occupational Outlook Handbook. https://www.bls.gov/ooh/fastest‐growing.htm

[62] BW Research Partnership . (2023). 2021 jobs study—March 2023: Vintage update. New York State Climate Action Council, Just Transition Working Group. https://climate.ny.gov/‐/media/Project/Climate/Files/JTWG‐Jobs‐Report‐Update.pdf

[63] Sovacool, B. K. , & Dworkin, M. H. (2015). Energy justice: Conceptual insights and practical applications. Applied Energy, 142, 435–444. 10.1016/j.apenergy.2015.01.002

[64] Mills, S. B. , Bessette, D. , & Smith, H. (2019). Exploring landowners’ post‐construction changes in perceptions of wind energy in Michigan. Land Use Policy, 82, 754–762. 10.1016/j.landusepol.2019.01.010

[65] U.S. Census Bureau . (2020). Profile of general population and housing characteristics (New York) . 2020 Decennial Census, Redistricting Data. https://data.census.gov/table/DECENNIALDP2020.DP1?g=040XX00US36,36$0500000&d=DECDemographicProfile

[66] New York State GIS Resources . (n.d.). Civil boundaries: Indian territories [Shapefile]. https://gis.ny.gov/civil‐boundaries

[67] Diaz‐Gonzalez, M. (2020). The complicated history of the Kinzua Dam and how it changed life for the Seneca people. Environmental Health News, https://www.ehn.org/seneca‐nation‐kinzua‐dam‐2644943791.html

[68] Szczepaniec, K. (2018). Indigenous people of Western New York. Partnership for the Public Good. https://ppgbuffalo.org/files/documents/data‐demographics‐history/indigenous_people_in_wny_final.pdf

[69] Berton, P. (1992). Niagara: A history of the falls. McClelland & Stewart.

[70] U.S. Energy Information Administration . (2000). Energy consumption and renewable energy development potential on Indian lands . Office of Coal, Nuclear, Electric and Alternate Fuels. https://www.energy.gov/sites/prod/files/2017/06/f34/EIA2000.pdf

[71] U.S. Department of Energy . (2015). Tribal energy system vulnerabilities to climate change and extreme weather. Office of Indian Energy. https://www.energy.gov/indianenergy/articles/tribal‐energy‐system‐vulnerabilities‐climate‐change‐and‐extreme‐weather

[72] Stone, L. (2014). Native energy: Rural electrification on Tribal lands. RMI. https://rmi.org/blog_2014_06_24_native_energy_rural_electrification_on_tribal_lands/

[73] Drehobl, A. , Ross, L. , & Ayala, R. (2020). How high are household energy burdens? American Council for an Energy‐Efficient Economy. https://www.aceee.org/sites/default/files/pdfs/u2006.pdf

[74] Shinnecock Indian Nation . (2013). Shinnecock Indian Nation climate change adaptation plan . https://www.epa.gov/sites/default/files/2016‐09/documents/shinnecock_nation_ccadaptation_plan_9.27.13.pdf

[75] Peconic Estuary Program & Shinnecock Indian Nation . (2019). Shinnecock Indian Nation climate vulnerability assessment and action plan . https://www.peconicestuary.org/wp‐content/uploads/2019/10/Shinnecock‐Indian‐Nation‐Climate‐Vulnerability‐Assessment‐and‐Action‐Plan.pdf

[76] Saint Regis Mohawk Tribe . (2013). Climate change adaptation plan for Akwesasne . https://www.cakex.org/sites/default/files/documents/ClimateChange.pdf

[77] Morales, K. (2021). Seneca Nation of Indians: Renewable energy for a changing climate. Climate Change Program, Institute for Tribal Environmental Professionals, Northern Arizona University. https://www7.nau.edu/itep/main/tcc/Tribes/ne_seneca

[78] Wall, D. (2011). Tuscarora: Drawing on traditional teaching to confront a changing climate. Institute for Tribal Environmental Professionals, Northern Arizona University. http://www7.nau.edu/itep/main/tcc/Tribes/ne_tuscarora

[79] New York State . (2023). Disadvantaged communities criteria. Climate Act Website. https://climate.ny.gov/resources/disadvantaged‐communities‐criteria/

[80] New York State Department of Environmental Conservation & New York State Energy Research and Development Authority . (2023). New York State's disadvantaged communities criteria . https://climate.ny.gov/‐/media/Project/Climate/Files/Disadvantaged‐Communities‐Criteria/LMI‐daccriteria‐fs‐1‐v2_acc.pdf

[81] Proceeding on motion of the commission concerning electric utility climate vulnerability studies and plans, 22‐E‐0222. (2022). https://climate.law.columbia.edu/sites/default/files/content/docs/PSC%20Climate%20Vulnerability%20Proceeding.pdf

[82] New York State Public Service Commission . (2022). PSC directs utilities to conduct climate vulnerability studies . https://dps.ny.gov/system/files/documents/2022/10/psc‐directs‐utilities‐to‐conduct‐climate‐vulnerability‐studies.pdf

[83] National Grid . (2021). National Grid TCFD physical climate change risk modelling (Final Report v5.00).

[84] Petrakopoulou, F. , Robinson, A. , & Olmeda‐Delgado, M. (2020). Impact of climate change on fossil fuel power‐plant efficiency and water use. Journal of Cleaner Production, 273, 122816. 10.1016/j.jclepro.2020.122816

[85] Miara, A. , & Vörösmarty, C. J. (2013). A dynamic model to assess tradeoffs in power production and riverine ecosystem protection. Environmental Science: Processes & Impacts, 15(6), 1095–1292. 10.1039/c3em00196b 23636670

[86] Electric Power Research Institute . (2022). A starting point for physical climate risk assessment and mitigation: Future resilience and adaptation planning . https://www.epri.com/research/products/000000003002024895

[87] Electric Power Research Institute . (2015). Power plant cooling system overview: Guidance for researchers and technology developers . https://www.epri.com/research/products/000000003002007194

[88] Jaglom, W. S. , McFarland, J. R. , Colley, M. F. , Mack, C. B. , Venkatesh, B. , Miller, R. L. , Haydel, J. , Schultz, P. A. , Perkins, B. , Casola, J. H. , Martinich, J. A. , Cross, P. , Kolian, M. J. , & Kayin, S. (2014). Assessment of projected temperature impacts from climate change on the U.S. electric power sector using the Integrated Planning Model®. Energy Policy, 73, 524–539. 10.1016/j.enpol.2014.04.032

[89] Murphy, S. , Sowell, F. , & Apt, J. (2019). A time‐dependent model of generator failures and recoveries captures correlated events and quantifies temperature dependence. Applied Energy, 253, 113513. 10.1016/j.apenergy.2019.113513

[90] Energy and Environmental Economics . (2023). Impacts of climate change on the New York energy system. New York State Energy and Research Development Authority. https://www.nyserda.ny.gov/About/Publications

[91] Dubey, S. , Sarvaiya, J. N. , & Seshadri, B. (2013). Temperature dependent photovoltaic (PV) efficiency and its effect on PV production in the world—A review. Energy Procedia, 33, 311–321. 10.1016/j.egypro.2013.05.072

[92] Segbefia, O. K. , & Sætre, T. O. (2022). Investigation of the temperature sensitivity of 20‐years old field‐aged photovoltaic panels affected by potential induced degradation. Energies, 15(11), 3865. 10.3390/en15113865

[93] Tobnaghi, D. M. , Madatov, R. , & Naderi, D. (2013). The effect of temperature on electrical parameters of solar cells. International Journal of Advanced Research in Electrical, Electronics and Instrumentation Engineering, 2(12), 6404–6407.

[94] Pryor, S. C. , & Barthelmie, R. J. (2013). Assessing the vulnerability of wind energy to climate change and extreme events. Climatic Change, 121(1), 79–91. 10.1007/s10584-013-0889-y

[95] Pryor, S. C. , & Barthelmie, R. J. (2010). Climate change impacts on wind energy: A review. Renewable and Sustainable Energy Reviews, 14(1), 430–437. 10.1016/j.rser.2009.07.028

[96] U.S. Chemical Storage . (2020). Lithium ion battery storage requirements . https://www.uschemicalstorage.com/lithium‐ion‐battery‐storage‐requirements/

[97] Jeevarajan, J. A. , Joshi, T. , Parhizi, M. , Rauhala, T. , & Juarez‐Robles, D. (2022). Battery hazards for large energy storage systems. ACS Energy Letters, 7(8), 2725–2733. 10.1021/acsenergylett.2c01400

[98] Pullen, K. R. (2022). Flywheel energy storage. In T. M. Letcher (Ed.), Storing energy (pp. 207–242). Elsevier. 10.1016/B978-0-12-824510-1.00035-0

[99] Trane . (n.d.). Thermal energy storage . https://www.trane.com/commercial/north‐america/us/en/products‐systems/energy‐storage‐solutions.html

[100] Arent, D. J. , Tol, R. S. J. , Faust, E. , Hella, J. P. , Kumar, S. , Strzepek, K. M. , Tóth, F. L. , & Yan, D. (2014). Chapter 10: Key economic sectors and services. In C. B. Field , V. R. Barros , D. J. Dokken , K. J. Mach , M. D. Mastrandrea , T. E. Bilir , M. Chatterjee , K. L. Ebi , Y. O. Estrada , R. C. Genova , B. Girma , E. S. Kissel , A. N. Levy , S. MacCracken , P. R. Mastrandrea , & L. L. White (Eds.), Climate change 2014: Impacts, adaptation, and vulnerability. Part A: Global and sectoral aspects. Contribution of Working Group II to the Fifth Assessment Report of the Intergovernmental Panel on Climate Change (pp. 659–708). Cambridge University Press. https://www.ipcc.ch/site/assets/uploads/2018/02/WGIIAR5‐Chap10_FINAL.pdf

[101] Johnston, P. C. , Gomez, J. F. , & Laplante, B. (2012). Climate risk and adaptation in the electric power sector. Asian Development Bank. https://www.adb.org/publications/climate‐risk‐and‐adaptation‐electric‐power‐sector

[102] Leonard, K. , Shaw, S. B. , Francis, A. , Hermann, D. , Josset, L. , May, C. L. , Wright, B. , Yokota, K. , & Stevens, A. (2024). New York State Climate Impacts Assessment Chapter 10: Water Resources. Annals of the New York Academy of Sciences, 1542, 561–619. 10.1111/nyas.15197 PMC11668508 39652428

[103] Seglenieks, F. , & Temgoua, A. (2022). Future water levels of the Great Lakes under 1.5°C to 3°C warmer climates. Journal of Great Lakes Research, 48(4), 865–875. 10.1016/j.jglr.2022.05.012

[104] Hudson River–Black River Regulating District . (2023). Hudson River–Black River Regulating District . https://hrbrrd.ny.gov/

[105] New York State . (2023). Upper Hudson River watershed. Hudson River–Black River Regulating District. https://hrbrrd.ny.gov/upper‐hudson‐river‐watershed/

[106] New York State Electric & Gas & Rochester Gas and Electric . (2011). Hurricane Irene and Tropical Storm Lee report . https://documents.dps.ny.gov/public/Common/ViewDoc.aspx?DocRefId=%7B294E4003‐6A70‐4312‐9116‐FD108475EF5D%7D

[107] Filippatos, A. , Dannemann, M. , Nguyen, M. , Brenner, D. , & Gude, M. (2020). Influence of ice accumulation on the structural dynamic behaviour of composite rotors. Applied Sciences, 10(15), 5063. 10.3390/app10155063

[108] Gao, L. , Tao, T. , Liu, Y. , & Hu, H. (2021). A field study of ice accretion and its effects on the power production of utility‐scale wind turbines. Renewable Energy, 167, 917–928. 10.1016/j.renene.2020.12.014

[109] Sweet, W. V. , Hamlington, B. D. , Kopp, R. E. , Weaver, C. P. , Barnard, P. L. , Bekaert, D. , Brooks, W. , Craghan, M. , Dusek, G. , Frederikse, T. , Garner, G. , Genz, A. S. , Krasting, J. P. , Larour, E. , Marcy, D. , Marra, J. J. , Obeysekera, J. , Osler, M. , Pendleton, M. , … Zuzak, C. (2022). Global and regional sea level rise scenarios for the United States: Updated mean projections and extreme water level probabilities along U.S. coastlines. (NOAA Technical Report NOS 01). National Oceanic and Atmospheric Administration. https://oceanservice.noaa.gov/hazards/sealevelrise/sealevelrise‐tech‐report.html

[110] Zamuda, C. D. , Bilello, D. E. , Carmack, J. , Davis, X. J. , Efroymson, R. A. , Goff, K. M. , Hong, T. , Karimjee, A. , Loughlin, D. H. , & Voisin, N. (2023). Chapter 5: Energy supply, delivery, and demand. In A. R. Crimmins , C. W. Avery , D. R. Easterling , K. E. Kunkel , B. C. Stewart , & T. K. Maycock (Eds.), Fifth National Climate Assessment. U.S. Global Change Research Program. 10.7930/NCA5.2023.CH5

[111] Arias, P. , Bellouin, N. , Coppola, E. , Jones, R. , Krinner, G. , Marotzke, J. , Naik, V. , Palmer, M. , Plattner, G.‐K. , Rogelj, J. , Rojas, M. , Sillmann, J. , Storelvmo, T. , Thorne, P. , Trewin, B. , Rao, K. , Adhikary, B. , Allan, R. , Armour, K. , … Zickfeld, K. (2021). Technical summary. In V. Masson‐Delmotte , P. Zhai , A. Pirani , S. L. Connors , C. Péan , Y. Chen , L. Goldfarb , M. Gomis , J. B. Robin Matthews , S. Berger , M. Huang , O. Yelekçi , R. Yu , B. Zhou , E. Lonnoy , T. K. Maycock , T. Waterfield , K. Leitzell , & N. Caud (Eds.), Climate change 2021: The physical science basis. Working Group I contribution to the sixth assessment report of the Intergovernmental Panel on Climate Change (pp. 33–144). Cambridge University Press. 10.1017/9781009157896.002

[112] Craig, M. T. , Cohen, S. , Macknick, J. , Draxl, C. , Guerra, O. J. , Sengupta, M. , Haupt, S. E. , Hodge, B.‐M. , & Brancucci, C. (2018). A review of the potential impacts of climate change on bulk power system planning and operations in the United States. Renewable and Sustainable Energy Reviews, 98, 255–267. 10.1016/j.rser.2018.09.022

[113] Freedman, J. , Manobianco, J. , Kirk‐Davidoff, D. B. , Gothandaraman, A. , Beaucage, P. , Xia, G. , Chen, S. , Covert, J. M. , Dai, A. , & Perez, R. (2019). High‐resolution dynamic downscaling of CMIP5 model data to assess the effects of climate change on renewable energy distribution in New York State. American Geophysical Union Fall Meeting. https://agu.confex.com/agu/fm19/meetingapp.cgi/Paper/515612

[114] Jerez, S. , Tobin, I. , Vautard, R. , Montávez, J. P. , López‐Romero, J. M. , Thais, F. , Bartok, B. , Christensen, O. B. , Colette, A. , Déqué, M. , Nikulin, G. , Kotlarski, S. , van Meijgaard, E. , Teichmann, C. , & Wild, M. (2015). The impact of climate change on photovoltaic power generation in Europe. Nature Communications, 6(1), 10014. 10.1038/ncomms10014 PMC4682048 26658608

[115] Patt, A. , Pfenninger, S. , & Lilliestam, J. (2013). Vulnerability of solar energy infrastructure and output to climate change. Climatic Change, 121(1), 93–102. 10.1007/s10584-013-0887-0

[116] Federal Energy Regulatory Commission & North American Electric Reliability Corporation . (2021). The February 2021 cold weather outages in Texas and the south central United States . https://www.ferc.gov/media/february‐2021‐cold‐weather‐outages‐texas‐and‐south‐central‐united‐states‐ferc‐nerc‐and

[117] Consolidated Edison Co. of New York, Inc . (2019). Climate change vulnerability study . https://cdne‐dcxprod‐sitecore.azureedge.net/‐/media/files/coned/documents/our‐energy‐future/our‐energy‐projects/climate‐change‐resiliency‐plan/climate‐change‐vulnerability‐study‐2019.pdf

[118] Consolidated Edison Co. of New York, Inc. & Orange and Rockland Utilities, Inc . (2013). Post Sandy enhancement plan . https://cdnc‐dcxprod2‐sitecore.azureedge.net/‐/media/files/coned/documents/services‐outages/post‐sandy‐enhancement‐plan.pdf

[119] U.S. Army Corps of Engineers . (2019). Global changes: Incorporating sea level change in civil works programs . https://www.publications.usace.army.mil/Portals/76/Users/182/86/2486/ER_1100‐2‐8162.pdf

[120] U.S. Environmental Protection Agency . (2020). Underground storage tank flood guide . https://www.epa.gov/sites/default/files/2014‐03/documents/ustfloodguide.pdf

[121] Steel Tank Institute/Steel Plate Fabricators Association . (2023). Newer fuels and storage tank corrosion . https://stispfa.org/facts/newer‐fuels‐and‐storage‐tank‐corrosion/

[122] U.S. Department of Energy . (2017). Transforming the nation's electricity system: The second installment of the QER (Quadrennial Energy Review). https://www.energy.gov/sites/prod/files/2017/02/f34/Quadrennial%20Energy%20Review–Second%20Installment%20%28Full%20Report%29.pdf

[123] Institute of Electrical and Electronics Engineers . (2012). IEEE guide for loading mineral‐oil‐immersed transformers and step‐voltage regulators . 10.1109/IEEESTD.2012.6166928

[124] Bartos, M. , Chester, M. , Johnson, N. , Gorman, B. , Eisenberg, D. , Linkov, I. , & Bates, M. (2016). Impacts of rising air temperatures on electric transmission ampacity and peak electricity load in the United States. Environmental Research Letters, 11(11), 114008. 10.1088/1748-9326/11/11/114008

[125] Sathaye, J. A. , Dale, L. L. , Larsen, P. H. , Fitts, G. A. , Koy, K. , Lewis, S. M. , & de Lucena, A. F. P. (2013). Estimating impacts of warming temperatures on California's electricity system. Global Environmental Change, 23(2), 499–511. 10.1016/j.gloenvcha.2012.12.005

[126] New York Power Pool Tie‐Line Ratings Task Force . (1995). Final report on tie‐line ratings . https://www.nyiso.com/documents/20142/1402024/nypp_tieline_ratings_report.pdf

[127] Ajenikoko, G. , & Adeleke, B. S. (2017). Effect of temperature change on the resistance of transmission line losses in electrical power network. International Journal of Renewable Energy Technology Research, 6(1), 1–8. http://ijretr.org/IJRETR_Vol.%206,%20No.%201,%20January%202017/EFFECT%20OF%20TEMPERATURE.pdf

[128] Consolidated Edison Co. of New York, Inc . (2023). Con Edison climate change vulnerability study . https://cdne‐dcxprod‐sitecore.azureedge.net/‐/media/files/coned/documents/our‐energy‐future/our‐energy‐projects/climate‐change‐resiliency‐plan/climate‐change‐vulnerability‐study.pdf

[129] Consolidated Edison Co. of New York, Inc . (2019). Climate change vulnerability study: Appendix 1—Temperature . https://cdnc‐dcxprod2‐sitecore.azureedge.net/‐/media/files/coned/documents/our‐energy‐future/our‐energy‐projects/climate‐change‐resiliency‐plan/climate‐change‐vulnerability‐study‐appendix.pdf

[130] Battaglia, S. M. , & Changnon, D. (2016). Frost quake events and changing wintertime air mass frequencies in Southeastern Canada. Department of Geography, Northern Illinois University. https://www.researchgate.net/publication/313444438_Frost_Quake_Events_and_Changing_Wintertime_Air_Mass_Frequencies_in_Southeastern_Canada

[131] T&D World . (2018). Flooding and underground cables: Myth or reality? https://www.tdworld.com/intelligent‐undergrounding/article/20971838/flooding‐and‐underground‐cables‐myth‐or‐reality

[132] National Fuel Gas Company . (2022). 2022 climate report . https://www.nationalfuel.com/wp‐content/uploads/documents/NFG‐2022‐Climate‐Report‐FINAL.pdf

[133] Rosenfeld, M. J. (2015). Cold weather can play havoc on natural gas systems. Pipeline & Gas Journal, 242(1). https://pgjonline.com/magazine/2015/january‐2015‐vol‐242‐no‐1/features/cold‐weather‐can‐play‐havoc‐on‐natural‐gas‐systems

[134] Bruzgul, J. , Kay, R. , Rodehorst, B. , Petrow, A. , Hendrickson, T. , Bruguera, M. , Petak, K. , Moreno, D. , Manik, J. , & Revell, D. (2018). Potential climate change impacts and adaptation actions for gas assets in the San Diego Gas and Electric Company service area. California Energy Commission. https://www.energy.ca.gov/sites/default/files/2019‐11/Energy_CCCA4‐CEC‐2018‐009_ADA.pdf

[135] U.S. Department of Transportation . (2023). Cast and wrought iron inventory. Pipeline and Hazardous Materials Safety Administration. https://www.phmsa.dot.gov/data‐and‐statistics/pipeline‐replacement/cast‐and‐wrought‐iron‐inventory

[136] Northeast Gas Association . (2014). Flood planning for natural gas utilities: Lessons learned and industry practices . https://www.northeastgas.org/pdf/flood_planning.pdf

[137] New York State Department of Public Service . (2012). Utility performance report following Hurricane Irene and Tropical Storm Lee . https://documents.dps.ny.gov/public/Common/ViewDoc.aspx?DocRefId=%7BAD5B750D‐A5DC‐4ABB‐972F‐EB0557269D9F%7D

[138] Harms, B. (2006). Water hammer in steam systems: Cause and effect. Plant Engineering. https://www.plantengineering.com/articles/water‐hammer‐in‐steam‐systems‐cause‐and‐effect/

[139] U.S. Department of Energy . (1995). Averting water hammers and other steam/condensate system incidents . https://www.swagelok.com/downloads/webcatalogs/en/Energy‐Advisors‐Water‐Hammer‐Article.pdf

[140] Thom, H. C. S. (1954). The rational relationship between heating degree days and temperature. Monthly Weather Review, 82(1), 1–6. 10.1175/1520-0493(1954)082<0001:TRRBHD>2.0.CO;2

[141] Romitti, Y. , & Wing, I. S. (2022). Heterogeneous climate change impacts on electricity demand in world cities circa mid‐century. Scientific Reports, 12(1), 4280. 10.1038/s41598-022-07922-w 35277550 PMC8917203

[142] van Ruijven, B. J. , De Cian, E. , & Sue Wing, I. (2019). Amplification of future energy demand growth due to climate change. Nature Communications, 10(1), 2762. 10.1038/s41467-019-10399-3 PMC6591298 31235700

[143] Dirks, J. A. , Gorrissen, W. J. , Hathaway, J. H. , Skorski, D. C. , Scott, M. J. , Pulsipher, T. C. , Huang, M. , Liu, Y. , & Rice, J. S. (2015). Impacts of climate change on energy consumption and peak demand in buildings: A detailed regional approach. Energy, 79, 20–32. 10.1016/j.energy.2014.08.081

[144] New York Independent System Operator . (2021). 2021–2030 comprehensive reliability plan . https://www.nyiso.com/documents/20142/2248481/2021‐2030‐Comprehensive‐Reliability‐Plan.pdf

[145] Itron, Inc . (2019). New York ISO climate change impact study phase 1: Long‐term load impact. New York Independent System Operator. https://www.nyiso.com/documents/20142/10773574/NYISO‐Climate‐Impact‐Study‐Phase1‐Report.pdf

[146] New York Independent System Operator . (2022). 2022 load & capacity data . https://www.nyiso.com/documents/20142/2226333/2022‐Gold‐Book‐Final‐Public.pdf

[147] New York Independent System Operator . (2023). Baseline forecast tables: Tables I‐1b,c,d . 2023 Gold Book Resources. https://www.nyiso.com/gold‐book‐resources

[148] New York State Climate Action Council . (2023). New York's scoping plan. Climate Act Website. https://climate.ny.gov/resources/scoping‐plan/

[149] New York Independent System Operator . (2023). IA tech supplement annex 2—Key drivers outputs—Electric load and peak by scenario tab . 2023 Gold Book Resources. https://www.nyiso.com/gold‐book‐resources

[150] Salamanca, F. , Georgescu, M. , Mahalov, A. , Moustaoui, M. , & Wang, M. (2014). Anthropogenic heating of the urban environment due to air conditioning. Journal of Geophysical Research: Atmospheres, 119(10), 5949–5965. 10.1002/2013JD021225

[151] Cohen, J. , Agel, L. , Barlow, M. , Garfinkel, C. I. , & White, I. (2021). Linking Arctic variability and change with extreme winter weather in the United States. Science, 373(6559), 1116–1121. 10.1126/science.abi9167 34516838

[152] Blackport, R. , Fyfe, J. C. , & Screen, J. A. (2022). Arctic change reduces risk of cold extremes. Science, 375(6582), 729–729. 10.1126/science.abn2414 35175808

[153] Petri, Y. , & Caldeira, K. (2015). Impacts of global warming on residential heating and cooling degree‐days in the United States. Scientific Reports, 5(1), 12427. 10.1038/srep12427 26238673 PMC4523835

[154] Markolf, S. A. , Chester, M. V. , Eisenberg, D. A. , Iwaniec, D. M. , Davidson, C. I. , Zimmerman, R. , Miller, T. R. , Ruddell, B. L. , & Chang, H. (2018). Interdependent infrastructure as linked social, ecological, and technological systems (SETSs) to address lock‐in and enhance resilience. Earth's Future, 6(12), 1638–1659. 10.1029/2018EF000926

[155] Markolf, S. A. , Hoehne, C. , Fraser, A. , Chester, M. V. , & Underwood, B. S. (2019). Transportation resilience to climate change and extreme weather events—Beyond risk and robustness. Transport Policy, 74, 174–186. 10.1016/j.tranpol.2018.11.003

[156] Haraguchi, M. , & Kim, S. (2016). Critical infrastructure interdependence in New York City during Hurricane Sandy. International Journal of Disaster Resilience in the Built Environment, 7(2), 133–143. 10.1108/IJDRBE-03-2015-0015

[157] Oikonomou, K. , Mongird, K. , Rice, J. S. , & Homer, J. S. (2021). Resilience of interdependent water and power systems: A literature review and conceptual modeling framework. Water, 13(20), 2846. 10.3390/w13202846

[158] Hotchkiss, E. (2021). Resilience in an age of increasing electrification. Current Sustainable/Renewable Energy Reports, 8, 174–179. 10.1007/s40518-021-00176-6

[159] Wei, W. , & Wang, J. (2020). Modeling and optimization of interdependent energy infrastructures. Springer Nature Switzerland AG. 10.1007/978-3-030-25958-7

[160] Amini, M. H. , Boroojeni, K. G. , Iyengar, S. S. , Pardalos, P. M. , Blaabjerg, F. , & Madni, A. M. (2019). Sustainable interdependent networks II: From smart power grids to intelligent transportation networks. Springer Nature Switzerland AG. 10.1007/978-3-319-98923-5

[161] Aller, D. , Chatrchyan, A. M. , Calixto, A. , Cummings, J. , Ortiz‐Bobea, A. , Peck, G. , Schouten, J. , Weikert, B. , Wolters, E. , & Stevens, A. (2024). New York State Climate Impacts Assessment Chapter 03: Agriculture. Annals of the New York Academy of Sciences, 1542, 146–213. 10.1111/nyas.15192 PMC11668505 39652373

[162] Electric Power Research Institute . (n.d.). Climate READi . https://www.epri.com/research/sectors/readi

[163] Guruswamy, L. (2011). Energy poverty. Annual Review of Environment and Resources, 36(1), 139–161. 10.1146/annurev-environ-040610-090118

[164] Teron, L. , & Ekoh, S. S. (2018). Energy democracy and the city: Evaluating the practice and potential of municipal sustainability planning. Frontiers in Communication, 3, 8. 10.3389/fcomm.2018.00008

[165] Reames, T. G. , Reiner, M. A. , & Stacey, M. B. (2018). An incandescent truth: Disparities in energy‐efficient lighting availability and prices in an urban U.S. county. Applied Energy, 218, 95–103. 10.1016/j.apenergy.2018.02.143

[166] Jessel, S. , Sawyer, S. , & Hernández, D. (2019). Energy, poverty, and health in climate change: A comprehensive review of an emerging literature. Frontiers in Public Health, 7, 357. 10.3389/fpubh.2019.00357 31921733 PMC6920209

[167] Hernández, D. (2016). Understanding “energy insecurity” and why it matters to health. Social Science & Medicine, 167, 1–10. 10.1016/j.socscimed.2016.08.029 27592003 PMC5114037

[168] New York City Mayor's Office of Sustainability & Mayor's Office for Economic Opportunity . (2019). Understanding and alleviating energy cost burden in New York City . https://www.nyc.gov/assets/sustainability/downloads/pdf/publications/EnergyCost.pdf

[169] U.S. Census Bureau . (2023). Household pulse survey: Measuring social and economic impacts during the coronavirus pandemic . https://www.census.gov/programs‐surveys/household‐pulse‐survey.html

[170] Ross, L. , Drehobl, A. , & Stickles, B. (2018). The high cost of energy in rural America: Household energy burdens and opportunities for energy efficiency. American Council for an Energy‐Efficient Economy. https://www.aceee.org/sites/default/files/publications/researchreports/u1806.pdf

[171] Garza, L. Y. , Anderson, C. , Caloras, A. , & Wazowicz, M. (2022). First to reside, last to benefit: A study of midwestern Tribal efficiency. Midwest Energy Efficiency Alliance. https://www.mwalliance.org/sites/default/files/meea‐research/first_to_reside_last_to_benefit‐_a_study_of_midwestern_tribal_efficiency_0.pdf

[172] U.S. Bureau of Labor Statistics . (2022). New York: Quintiles of income before taxes. Consumer Expenditure Surveys. https://www.bls.gov/cex/tables/geographic/mean/cu‐state‐ny‐income‐quintiles‐before‐taxes‐2‐year‐average‐2020.htm

[173] U.S. Department of Energy . (2019). Low‐Income Energy Affordability Data (LEAD) tool. State and Local Solution Center. https://www.energy.gov/scep/slsc/low‐income‐energy‐affordability‐data‐lead‐tool

[174] Ma, O. , Laymon, K. , Day, M. , Oliveira, R. , Weers, J. , & Vimont, A. (2019). Low‐Income Energy Affordability Data (LEAD) tool methodology. National Renewable Energy Laboratory. https://www.nrel.gov/docs/fy19osti/74249.pdf

[175] Berry, C. , Hronis, C. , & Woodward, M. (2018). Who's energy insecure? You might be surprised. ACEEE Summer Study on Energy Efficiency in Buildings.

[176] Lewis, J. , Hernández, D. , & Geronimus, A. T. (2020). Energy efficiency as energy justice: Addressing racial inequities through investments in people and places. Energy Efficiency, 13(3), 419–432. 10.1007/s12053-019-09820-z PMC7966972 33737861

[177] Hernández, D. (2013). Energy insecurity: A framework for understanding energy, the built environment, and health among vulnerable populations in the context of climate change. American Journal of Public Health, 103(4), e32–e34. 10.2105/AJPH.2012.301179 23409876 PMC3673265

[178] U.S. Census Bureau . (2023). Week 63 household pulse survey: October 18–October 30 . https://www.census.gov/data/tables/2023/demo/hhp/hhp63.html

[179] Bird, S. , & Hernández, D. (2012). Policy options for the split incentive: Increasing energy efficiency for low‐income renters. Energy Policy, 48, 506–514. 10.1016/j.enpol.2012.05.053 27053828 PMC4819331

[180] U.S. Energy Information Administration . (2015). 2015 RECS survey data. Residential Energy Consumption Survey (RECS). https://www.eia.gov/consumption/residential/data/2015/

[181] Verclas, K. , & Hsieh, E. (2018). From utility disconnection to universal access. Electricity Journal, 31(6), 1–8. 10.1016/j.tej.2018.06.006

[182] Goff, C. , Amarakoon, S. , Curtis, D. , & Stevens, A. (2024). New York State Climate Impacts Assessment Chapter 01: Introduction. Annals of the New York Academy of Sciences, 1542, 67–90. 10.1111/nyas.15202 PMC11668495 39652326

[183] Bhattacharya, J. , DeLeire, T. , Haider, S. , & Currie, J. (2003). Heat or eat? Cold‐weather shocks and nutrition in poor American families. American Journal of Public Health, 93(7), 1149–1154. 10.2105/AJPH.93.7.1149 12835201 PMC1447925

[184] Pastor, M. , Bullard, R. , Boyce, J. K. , Fothergill, A. , Morello‐Frosch, R. , & Wright, B. (2006). Environment, disaster and race after Katrina. Race, Poverty & the Environment, 13(1), 21–26. http://www.jstor.org/stable/41495680

[185] Marcotullio, P. J. , Braçe, O. , Lane, K. , Olson, C. E. , Tipaldo, J. , Ventrella, J. , Yoon, L. , Knowlton, K. , Anand, G. , & Matte, T. (2023). Local power outages, heat, and community characteristics in New York City. Sustainable Cities and Society, 99, 104932. 10.1016/j.scs.2023.104932

[186] Alper, G. (2021). Do heat vulnerable neighborhoods in New York City experience disproportionate power outages? City University of New York Hunter College. https://academicworks.cuny.edu/cgi/viewcontent.cgi?article=1805&context=hc_sas_etds

[187] Sotolongo, M. , Kuhl, L. , & Baker, S. H. (2021). Using environmental justice to inform disaster recovery: Vulnerability and electricity restoration in Puerto Rico. Environmental Science & Policy, 122, 59–71. 10.1016/j.envsci.2021.04.004

[188] Hernández, D. , & Siegel, E. (2019). Energy insecurity and its ill health effects: A community perspective on the energy‐health nexus in New York City. Energy Research & Social Science, 47, 78–83. 10.1016/j.erss.2018.08.011 32280598 PMC7147484

[189] Nord, M. , & Kantor, L. S. (2006). Seasonal variation in food insecurity is associated with heating and cooling costs among low‐income elderly Americans. Journal of Nutrition, 136(11), 2939–2944. 10.1093/jn/136.11.2939 17056826

[190] Hernández, D. , & Bird, S. (2010). Energy burden and the need for integrated low‐income housing and energy policy. Poverty & Public Policy, 2(4), 5–25. 10.2202/1944-2858.1095 27053989 PMC4819257

[191] U.S. Environmental Protection Agency . (2023). Climate change and human health: Who's most at risk? Climate Change Impacts. https://www.epa.gov/climateimpacts/climate‐change‐and‐human‐health‐whos‐most‐risk

[192] Lebel, E. D. , Finnegan, C. J. , Ouyang, Z. , & Jackson, R. B. (2022). Methane and NO* _x_ * emissions from natural gas stoves, cooktops, and ovens in residential homes. Environmental Science & Technology, 56(4), 2529–2539. 10.1021/acs.est.1c04707 35081712

[193] U.S. Environmental Protection Agency . (2022). Climate change and the health of workers . Climate Change Impacts. https://www.epa.gov/climateimpacts/climate‐change‐and‐health‐workers

[194] Kroposki, B. , Garrett, B. , MacMillan, S. , Rice, B. , Komomua, C. , O'Malley, M. , & Zimmerle, D. (2012). Energy systems integration: A convergence of ideas. National Renewable Energy Laboratory. 10.2172/1046325

[195] New York Independent System Operator . (2022). Power trends 2022: The path to a reliable, greener grid for New York . https://www.nyiso.com/documents/20142/2223020/2022‐Power‐Trends‐Report.pdf

[196] The GridWise Alliance . (2013). Improving electric grid reliability and resilience: Lessons learned from Superstorm Sandy and other extreme events . https://www.energy.gov/sites/prod/files/2015/03/f20/GridWise%20Improving%20Electric%20Grid%20Reliability%20and%20Resilience%20Report%20June%202013.pdf

[197] Freedman, J. M. , Perez, R. , Brotzge, J. A. , & Thorncroft, C. D. (2019). Toward 100 percent renewable energy in New York. University at Albany–State University of New York. https://static1.squarespace.com/static/5ac18fcc5cfd7975ce841e89/t/5e0a5f2ffbd38910d3ca00c4/1577738236085/NYRenewables20191218.pdf

[198] New York State Reliability Council . (2022). Development of NYSRC rules for mitigating extreme system conditions . https://www.nysrc.org/PDF/Documents/Extreme%20Conditions%20White%20Paper%20‐%20EC%20Approved%20%207‐8‐22.pdf

[199] Mauch, B. , Millar, D. , & Dorris, G. (2022). Resource adequacy modeling for a high renewable future. (NRRI Insights). National Regulatory Research Institute. https://pubs.naruc.org/pub/DC366C78‐1866‐DAAC‐99FB‐4C0759DB57C5

[200] New York State Reliability Council . (2022). Reliability rules & compliance manual for planning and operating the New York State power system . https://www.nysrc.org/PDF/Reliability%20Rules%20Manuals/RRC%20Manual%20V46%20final.pdf

[201] American Gas Association . (2014). Natural gas pipeline systems: Delivering resiliency . https://www.energy.gov/sites/prod/files/2015/04/f21/AGA%20QER%20Comments%20‐%20System%20Resiliency.pdf

[202] Phillips, T. , McJunkin, T. , Rieger, C. , Gardner, J. , & Mehrpouyan, H. (2020). A framework for evaluating the resilience contribution of solar PV and battery storage on the grid. 2020 Resilience Week (RWS). 10.1109/RWS50334.2020.9241296

[203] U.S. Department of Energy . (2015). Climate change and the U.S. energy sector: Regional vulnerabilities and resilience solutions. Office of Energy Policy and Systems Analysis. https://www.energy.gov/sites/prod/files/2015/10/f27/Regional_Climate_Vulnerabilities_and_Resilience_Solutions_0.pdf

[204] U.S. Department of Energy . (2013). U.S. energy sector vulnerabilities to climate change and extreme weather . https://www.energy.gov/articles/us‐energy‐sector‐vulnerabilities‐climate‐change‐and‐extreme‐weather

[205] UL Solutions . (n.d.). PV module certification . https://www.ul.com/services/pv‐module‐certification

[206] Shu, J. , Qu, J. J. , Motha, R. , Xu, J. C. , & Dong, D. F. (2018). Impacts of climate change on hydropower development and sustainability: A review. IOP Conference Series: Earth and Environmental Science, 163, 012126. 10.1088/1755-1315/163/1/012126

[207] New York Power Authority . (2014). N.Y. Power Authority post‐Sandy measures strengthening electricity infrastructure and resilience against future extreme weather events . https://www.nypa.gov/news/press‐releases/2014/20141030‐nypa‐strengthening‐electricity‐infrastructure

[208] New York State Energy Research and Development Authority . (2021). Offshore wind climate adaptation and resilience study . https://www.nyserda.ny.gov/‐/media/Project/Nyserda/Files/Programs/Offshore‐Wind/Offshore‐Wind‐Climate‐Adaptation‐and‐Resilience‐Study.pdf

[209] D'Aprile, P. , Geissmann, T. , López, F. P. , González, J. R. , & Tai, H. (2021). How to increase grid resilience through targeted investments. McKinsey & Company. https://www.mckinsey.com/~/media/mckinsey/industries/electric%20power%20and%20natural%20gas/our%20insights/how%20to%20increase%20grid%20resilience%20through%20targeted%20investments/how‐to‐increase‐grid‐resilience‐through‐targeted‐investments‐vf.pdf

[210] Assembly Bill 3360, New York State Assembly, 2021–2022 Legislative Session . (2021). https://www.nysenate.gov/legislation/bills/2021/A3360

[211] New York Senate Bill S4824A, New York State Senate, 2021–2022 Legislative Session . (2021). https://www.nysenate.gov/legislation/bills/2021/S4824

[212] New York State Department of Public Service . (n.d.). 22‐E‐0222 . https://documents.dps.ny.gov/public/MatterManagement/CaseMaster.aspx?MatterSeq=67333&MNO=22‐E‐0222

[213] Federal Energy Regulatory Commission . (2023). AD21‐13‐000 . https://elibrary.ferc.gov/eLibrary/docketsheet?docket=ad21‐13‐000

[214] North American Transmission Forum . (2023). Strengthen your transmission resilience. Transmission Resilience Maturity Model. https://trmm.labworks.org/

[215] Dyson, M. , & Li, B. X. (2020). Reimagining grid resilience: A framework for addressing catastrophic threats to the U.S. electricity grid in an era of transformational change. Rocky Mountain Institute. http://www.rmi.org/insight/reimagining‐grid‐resilience

[216] New York City Mayor's Office of Resiliency . (2020). Climate resiliency design guidelines . https://www.nyc.gov/assets/sustainability/downloads/pdf/publications/CRDG‐4‐1‐May‐2022.pdf

[217] NYS 2100 Commission . (2013). Recommendations to improve the strength and resilience of the Empire State's infrastructure .

[218] Environmental Defense Fund . (2013). Sandy success stories . https://www.edf.org/sites/default/files/sites/default/files/content/SandySuccessStories_June2013.pdf

[219] White, D. (2017). System hardening with 69‐kV spacer cable. *T&D World*. https://www.tdworld.com/grid‐innovations/transmission/article/20969152/system‐hardening‐with‐69kv‐spacer‐cable

[220] Mauldin, P. (2014). Storm hardening the grid. *T&D World*. https://www.tdworld.com/grid‐innovations/distribution/article/20964810/storm‐hardening‐the‐grid

[221] Gorman, W. , Barbose, G. , Carvallo, J. P. , Baik, S. , Miller, C. , White, P. , & Praprost, M. (2022). Evaluating the capabilities of behind‐the‐meter solar‐plus‐storage for providing backup power during long‐duration power interruptions. Lawrence Berkeley National Laboratory. https://eta‐publications.lbl.gov/sites/default/files/pvess_report_final.pdf

[222] Strbac, G. (2008). Demand side management: Benefits and challenges. Energy Policy, 36(12), 4419–4426. 10.1016/j.enpol.2008.09.030

[223] Creutzig, F. , Fernandez, B. , Haberl, H. , Khosla, R. , Mulugetta, Y. , & Seto, K. C. (2016). Beyond technology: Demand‐side solutions for climate change mitigation. Annual Review of Environment and Resources, 41(1), 173–198. 10.1146/annurev-environ-110615-085428

[224] Voisin, N. , Tidwell, V. , Kintner‐Meyer, M. , & Boltz, F. (2019). Planning for sustained water‐electricity resilience over the U.S.: Persistence of current water‐electricity operations and long‐term transformative plans. Water Security, 7, 100035. 10.1016/j.wasec.2019.100035

[225] National Academies of Sciences, Engineering, and Medicine . (2017). Chapter 6: Restoring grid function after a major disruption. In Enhancing the resilience of the nation's electricity system (pp. 110–133). National Academies Press. 10.17226/24836

[226] Federal Energy Regulatory Commission & North American Electric Reliability Corporation . (2016). Report on the FERC‐NERC‐Regional entity joint review of restoration and recovery plans . https://www.ferc.gov/sites/default/files/2020‐04/01‐29‐16‐FERC‐NERC‐Report.pdf

[227] Carley, S. , Engle, C. , Konisky, D. , & Sullivan, S. (2019). Supporting frontline and vulnerable communities in a Green New Deal. Public Administration Review (Blog), https://www.publicadministrationreview.com/2019/07/16/gnd17/

[228] Urban Jobs Task Force, Inc. & Legal Services of Central New York, Inc . (2019). Building equity in the construction trades: A racial equity impact statement . https://www.ujtfs.org/reis

[229] Frick, N. M. , Carvallo, J. P. , & Schwartz, L. C. (2021). Quantifying grid reliability and resilience impacts of energy efficiency: Examples and opportunities. Lawrence Berkeley National Laboratory. 10.2172/1834369

[230] Ribeiro, D. , Mackres, E. , Baatz, B. , Cluett, R. , Jarrett, M. , Kelly, M. , & Vaidyanathan, S. (2015). Enhancing community resilience through energy efficiency. American Council for an Energy‐Efficient Economy. https://www.aceee.org/research‐report/u1508

[231] Gamarro, H. , Ortiz, L. , & González, J. E. (2020). Adapting to extreme heat: Social, atmospheric, and infrastructure impacts of air‐conditioning in megacities—The case of New York City. ASME Journal of Engineering for Sustainable Buildings and Cities, 1(3), 031005. 10.1115/1.4048175

[232] City of New York . (2016). Cool Neighborhoods NYC: A comprehensive approach to keep communities safe in extreme heat . https://www.nyc.gov/assets/orr/pdf/Cool_Neighborhoods_NYC_Report.pdf

[233] City of Rochester, Office of Energy & Sustainability . (2019). Climate change resilience plan . https://www.cityofrochester.gov/WorkArea/DownloadAsset.aspx?id=21474842589#:~:text=The%20Rochester%20Climate%20Change%20Resilience,the%20impacts%20of%20climate%20change

[234] Kretschmer, F. , Hrdy, B. , Neugebauer, G. , & Stoeglehner, G. (2021). Wastewater treatment plants as local thermal power stations—Modifying internal heat supply for covering external heat demand. Processes, 9(11), 1981. 10.3390/pr9111981

[235] New York State Energy Research and Development Authority . (n.d.). Heating & cooling systems . https://www.nyserda.ny.gov/Residents‐and‐Homeowners/Heat‐and‐Cool‐Your‐Home/Heating‐Systems

[236] Hsiang, S. , Kopp, R. , Jina, A. , Rising, J. , Delgado, M. , Mohan, S. , Rasmussen, D. J. , Muir‐Wood, R. , Wilson, P. , Oppenheimer, M. , Larsen, K. , & Houser, T. (2017). Estimating economic damage from climate change in the United States. Science, 356(6345), 1362–1369. 10.1126/science.aal4369 28663496

[237] Rajkovich, N. B. , Brown, C. , Azaroff, I. , Backus, E. , Clarke, S. , Enriquez, J. , Greenaway, B. , Holtan, M. T. , Lewis, J. , Ornektekin, O. , Schoeman, L. , & Stevens, A. (2024). New York State Climate Impacts Assessment Chapter 04: Buildings. Annals of the New York Academy of Sciences, 1542, 214–252. 10.1111/nyas.15200 PMC11668501 39652388

[238] Electric Power Research Institute . (2022). Energy storage roadmap: 2022 update . https://www.epri.com/research/products/3002024676

[239] New York State Department of Public Service & New York State Energy Research and Development Authority . (2022). New York's 6 GW energy storage roadmap: Policy options for continued growth in energy storage . https://www.nyserda.ny.gov/‐/media/Project/Nyserda/Files/Programs/Energy‐Storage/ny‐6‐gw‐energy‐storage‐roadmap.pdf

[240] E2, Alliance to Save Energy, American Association of Blacks in Energy, Energy Efficiency for All, Black Owners of Solar Services, & BW Research Partnership . (2021). Help wanted: Diversity in clean energy . https://e2.org/wp‐content/uploads/2021/09/E2‐ASE‐AABE‐EEFA‐BOSS‐Diversity‐Report‐2021.pdf

[241] Adger, W. N. (2006). Vulnerability. Global Environmental Change, 16(3), 268–281. 10.1016/j.gloenvcha.2006.02.006

[242] New York State Department of Environmental Conservation . (n.d.). Climate change effects and impacts . https://dec.ny.gov/environmental‐protection/climate‐change/effects‐impactshttps://www.dec.ny.gov/energy/94702.html

[243] Kunz, M. , Mühr, B. , Kunz‐Plapp, T. , Daniell, J. E. , Khazai, B. , Wenzel, F. , Vannieuwenhuyse, M. , Comes, T. , Elmer, F. , Schröter, K. , Fohringer, J. , Münzberg, T. , Lucas, C. , & Zschau, J. (2013). Investigation of Superstorm Sandy 2012 in a multi‐disciplinary approach. Natural Hazards and Earth System Sciences, 13(10), 2579–2598. 10.5194/nhess-13-2579-2013

[244] New York Independent System Operator . (2023). 2023 load & capacity data . https://www.nyiso.com/documents/20142/2226333/2023‐Gold‐Book‐Public.pdf

[245] U.S. Census Bureau . (n.d.). Hamilton County, New York; Rochester City, New York; Syracuse City, New York; New York; New York City, New York. QuickFacts. https://www.census.gov/quickfacts/fact/table/hamiltoncountynewyork,rochestercitynewyork,syracusecitynewyork,NY,newyorkcitynewyork/PST045221

